# Design, synthesis, and activity evaluation of 2-iminobenzimidazoles as c-Myc inhibitors for treating multiple myeloma

**DOI:** 10.1016/j.heliyon.2024.e28411

**Published:** 2024-03-26

**Authors:** Shihao Li, Yinchuan Wang, Jiacheng Yin, Kaihang Li, Linlin Liu, Jian Gao

**Affiliations:** a Jiangsu Key Laboratory of New Drug Research and Clinical Pharmacy, Xuzhou Medical University, Xuzhou, Jiangsu 221004, PR China; b College of Medical Imaging, Xuzhou Medical University, Xuzhou, Jiangsu 221004, PR China; c School of Medicine, Anhui University of Science and Technology, Huainan, PR China

**Keywords:** Multiple myeloma, c-Myc, 2-Iminobenzimidazoles, Molecular docking

## Abstract

Multiple myeloma (MM) is a plasma cell malignancy that remains incurable and poses a significant threat to global public health. The multifunctional transcription factor c-Myc plays a crucial role in various cellular processes and is closely associated with MM progression. As part of the basic-helix-loop-helix-leucine zipper (bHLHZip) family, c-Myc forms heterodimers with its obligate partner Max, binds to the Enhancer-box (E-box) of DNA, and ultimately co-regulates gene expression. Therefore, impeding the capacity for heterodimerization to bind to DNA represents a favored strategy in thwarting c-Myc transcription. In this study, we first synthesized a series of novel 2-iminobenzimidazole derivatives and further estimated their potential anti-MM activity. Notably, among all the derivatives, **5b** and **5d** demonstrated remarkable inhibitory activity against RPMI-8226 and U266 cells, with IC_50_ values of 0.85 μM and 0.97 μM for compound **5b**, and 0.96 μM and 0.89 μM for compound **5d**. Western blot and dual-luciferase reporter assays demonstrated that compounds **5b** and **5d** effectively suppressed both c-Myc protein expression and transcriptional activity of the c-Myc promoter in RPMI-8226 and U266 cells. Furthermore, these compounds induced apoptosis and G1 cell cycle arrest in the aforementioned MM cells. Molecular docking studies revealed that **5b** and **5d** exhibited strong binding affinity to the interface between c-Myc/Max and E-box of DNA. Taken together, our findings suggest that further investigations are warranted for potential therapeutic applications of **5b** and **5d** for c-Myc-related diseases.

## Graphical abstract

2-iminobenzimidazoles as potential c-Myc inhibitors for treating multiple myeloma.

**Figure:**
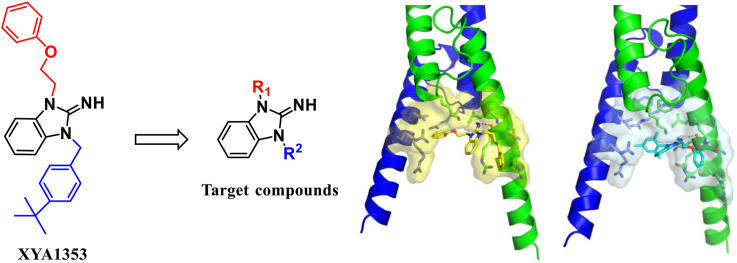

## 1 Introduction

Multiple myeloma (MM), the second most common blood cancer, is a plasma cell malignancy frequently accompanied by hypercalcemia, immune dysfunction, and renal failure [[1], [2], [3]]. Despite the employment of various therapeutic methods, such as immunomodulatory agents, proteasome inhibitors, and autologous stem cell transplantation (ASCT), MM remains an incurable disease with nearly 160,000 new cases and 106,000 deaths annually [4,5]. Unfortunately, relapse and drug resistance continue to pose significant challenges in the management of MM [6,7]. Therefore, it is imperative to discover novel drugs that exhibit superior selectivity and potency in the treatment of MM.

c-Myc, a crucial transcription factor and member of the MYC family, is closely linked to oncogenesis and implicated in various cellular processes [[8], [9], [10]]. Increasing evidence suggests that it functions as a primary oncogene in MM [11]. Given its crucial role in regulating cellular function, c-Myc is a natural target for drug development. However, due to its short half-life of only 20–30 min and lack of a specific drug-binding pocket, direct interaction with drugs remains challenging [12,13]. In the basic helix-loop-helix leucine zipper (bHLHZip) family, c-Myc interacts with Max (Myc associated factor X), another bHLHZip protein that serves as the obligatory partner of c-Myc, to form the c-Myc/Max heterodimerization. This process is regulated by the coiling of their respective bHLHZip domains [[14], [15], [16]]. Upon the formation of the c-Myc/Max heterodimer, it recognizes and binds to the E-box, a DNA sequence 5′-CACGTG-3′, ultimately leading to transcriptional activation [17,18]. Therefore, c-Myc is widely regarded as one of the most valuable targets in cancer research.

A plethora of strategies, both direct and indirect, have been employed to target c-Myc by exploiting its multiple regulatory mechanisms, encompassing c-Myc transcription and mRNA stability, c-Myc protein stability and degradation, as well as c-Myc binding to its interactome Max [19]. In 2003, Yin et al. [99] discovered three compounds (10058-F4, 10074-G5, and 10074-A4) that block the c-Myc/Max interaction, specifically inhibit Myc transcriptional activity and reduce cell growth in Myc-transformed rat fibroblasts [20]. However, these chemicals have limited clinical applicability due to their low potency, lack of selectivity, and poor pharmacokinetic behavior *in vivo* [21,22]. Despite significant efforts to optimize the two compounds, namely their derivatives JY-3-094 and 3jc48-3, resulting in varying degrees of improved potency, animal models have yet to demonstrate satisfactory tumor reduction performance [[23], [24], [25]]. Inhibiting c-Myc transcriptional activity can be also achieved by blocking the direct binding of c-Myc/Max to DNA using natural compounds like celastrol and celastrol-inspired triterpenoids [26], synthetic mimetics such as JKY-2-169 [27], or small molecule inhibitors including MYRA-A [28], NSC308848 [29], and KSI-3716 [30]. To date, the US Food and Drug Administration (FDA) has not approved any c-Myc drugs, thus emphasizing the pressing need to cultivate increasingly innovative inhibitors targeting c-Myc.

**Fig. 1:**
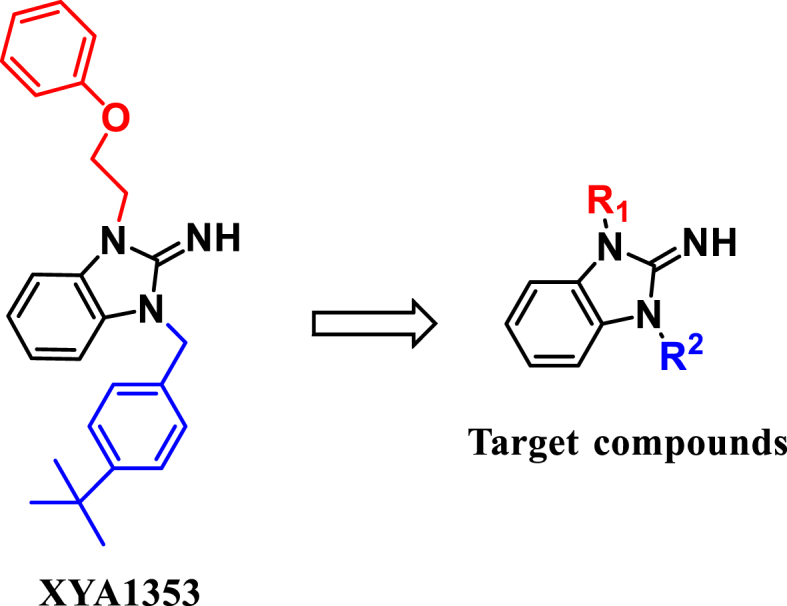
Chemical structures of XYA1353 and target compounds.

Virtually, we have been dedicating ourselves to seeking and discovering newer and more potential c-Myc inhibitors [[31], [32], [33], [34], [35]]. Recently, we discovered a new 2-iminobenzimidazole compound, XYA1353 (Fig. 1), which exhibits anti-MM activity both *in vitro* and *in vivo* by disrupting the canonical NF-κB signaling pathway through reducing expression of P65/P50 and phosphorylation levels of *p*-IκBα [36]. To optimize the lead compound XYA1353, a range of novel 2-iminobenzimidazole derivatives (Fig. 1) were designed and further elevated their anti-MM activity. We discovered compounds **5b** and **5d** as the most potent anti-MM agents, with their IC_50_ values being below approximately 1 μM. Moreover, these compounds demonstrated promise as inhibitors of c-Myc by effectively reducing the expression of c-Myc at both the mRNA and protein levels, as well as inhibiting the transcriptional activity of the c-Myc promoter.

## 2 Materials and methods

### 2.1 Chemistry

All starting regents were commercially available and solvents were used without any purification process unless noted. Each step purification of synthesized compounds was carried out with silica gel (purchased from Qingdao Ocean Chemical Co. Ltd) column chromatography. ^1^H and ^13^C NMR spectra were determined on a JEOL spectrometer in CDCl_3_ or DMSO‑*d*_6_. HRMS was analyzed via the Agilent 6550 Q-TOF instrument. Target compounds’ melting points were attained by using a YRT-3 apparatus.

### 2.2 Synthesis and characterization of **5a-5d**

#### 2.2.1 Synthesis of **1** (2-nitro-N-(2-phenoxyethyl)aniline)

A mixture containing 10 mmol of 2-nitroaniline and 20 mmol of NaH in 10 mL of DMF was chilled to 0°C. Subsequently, the mixture was treated with 13 mmol of 2-phenoxyethyl bromide, followed by heating the resulting combination at a temperature of 120 °C for 1 h. Upon cooling the solution to room temperature, it was poured into water and extracted with EA. The organic phase was washed with brine, dried over anhydrous Na_2_SO_4_, and subsequently concentrated under vacuum. Compound **1** was obtained after purification of the residue through column chromatography using a mixture of PE and EA in a ratio of 30:1 (v/v).

#### 2.2.2 Synthesis of **2** (N1-(2-phenoxyethyl)benzene-1,2-diamine)

The water solution (10 ml) of NH_4_Cl (35 ml) was gradually mixed with the THF (40 ml) solution of compound 1 (7 mmol), subsequently, the iron powder (35 mmol) was added and the mixture was stirred at 70 °C for 10h. After monitoring by TLC, the mixture above was filtered and the filtrate was collected, dried over anhydrous Na_2_SO_4,_ and concentrated to get crude product, which was purified by column chromatography using PE/EA = 5/1 (v/v) to afford compound **2**.

#### 2.2.3 Synthesis of **3a-3d**

Intermediate 2 (3.94 mmol) was with various substituted benzyl bromides (5.12 mmol) and K_2_CO_3_ (5.12 mmol) in DMF solution at 120 °C for 2 h. After monitoring by TLC, the reaction solution was cooled, poured into water, and extracted with EA. The organic phase was collected, washed with brine, and dried over anhydrous Na_2_SO_4_. By concentration under vacuum, the residue was purified via column chromatography using PE/EA = 10/1 (v/v) to give compounds **3a-3d**.

#### 2.2.4 Synthesis of **4a-4d**

The methanol solution of compound **3a-3d** (1.94 mmol) was treated with a dropwise addition of BrCN solution (2.33 mmol) in dichloromethane (8 mL). Following stirring at room temperature for 10 h, the solvent was evaporated and the resulting mixture was then adjusted to a pH value of 8–9 using saturated NaHCO_3_ aqueous solution before undergoing extraction with dichloromethane. The combined organic phase was subsequently washed with brine, dried using anhydrous Na_2_SO_4_, and concentrated under vacuum to obtain compounds **4a-4d**.

#### 2.2.5 Synthesis of **5a-5d**

The ethyl ether solution of compounds **4a-4d** (1.70 mmol) was combined with an aqueous solution of KOH (2.57 mmol), and the resulting mixture was stirred for 0.5 h. Upon completion of the reaction, the mixture was quenched with brine, extracted with ethyl ether, dried over anhydrous Na_2_SO_4_, and evaporated to afford final compounds **5a-5d**.

1-(4-isopropylbenzyl)-3-(2-phenoxyethyl)-1,3-dihydro-2H-benzo[*d*]imidazole-2-imine (**5a**). White solid, yield: 97%; M.p. 126.4–128.1 °C. ^1^H NMR (400 MHz, DMSO‑*d*_6_): *δ* 7.29–7.17 (m, 4H), 7.14 (d, *J* = 8.1 Hz, 2H), 7.05 (d, *J* = 7.4 Hz, 1H), 6.94–6.79 (m, 6H), 5.00 (s, 2H), 4.27–4.16 (m, 4H), 2.81 (dt, *J* = 13.8, 6.9 Hz, 1H), 1.19–1.08 (m, 6H). ^13^C NMR (100 MHz, DMSO‑*d*_6_): *δ* ppm 158.22, 153.30, 147.34, 134.82, 131.95, 131.36, 129.55, 127.34, 126.41, 120.78, 120.03, 119.92, 114.41, 106.70, 106.39, 65.24, 43.53, 40.68, 33.13, 23.90. ESI-HRMS [M+H]^+^ *m*/*z*:386.2222, calcd for C_25_H_27_N_3_O, 386.2227.

1-(2-phenoxyethyl)-3-(4-phenylbutyl)-1,3-dihydro-2H-benzo[*d*]imidazole-2-imine (**5b**). White solid, yield: 90%; M.p. 114.1–116.0 °C. ^1^H NMR (400 MHz, DMSO‑*d*_6_): *δ* 7.28–7.19 (m, 4H), 7.19–7.10 (m, 3H), 7.07–6.97 (m, 1H), 6.94–6.82 (m, 6H), 4.22–4.12 (m, 4H), 3.81 (t, *J* = 6.5 Hz, 2H), 2.59 (t, *J* = 7.1 Hz, 2H), 1.65–1.54 (m, 4H). ^13^C NMR (100 MHz, DMSO‑*d*_6_): *δ* ppm 158.18, 153.05, 142.05, 131.86, 131.47, 129.51, 128.35, 128.32, 128.26, 125.70, 120.74, 119.85, 119.71, 114.36, 106.56, 105.95, 65.21, 40.55, 40.33, 34.85, 28.23, 27.07. ESI-HRMS [M+H]^+^ *m*/*z*:386.2221 calcd for C_25_H_27_N_3_O, 386.2227.

1-(4-fluorobenzyl)-3-(2-phenoxyethyl)-1,3-dihydro-2H-benzo[*d*]imidazole-2-imine (**5c**). White solid, yield: 91%; M.p. 145.1–146.9 °C. ^1^H NMR (400 MHz, DMSO‑*d*_6_): *δ* 7.36–7.27 (m, 2H), 7.25–7.17 (m, 2H), 7.08 (t, *J* = 8.9 Hz, 2H), 7.01 (s, 1H), 6.88 (d, *J* = 7.3 Hz, 1H), 6.86–6.77 (m, 5H), 5.00 (s, 2H), 4.23–4.13 (m, 4H). ^13^C NMR (100 MHz, DMSO‑*d*_6_): *δ* ppm 162.58, 160.16, 158.20, 153.21, 133.69, 131.97, 131.18, 129.54, 129.45, 129.37, 120.78, 120.10, 119.94, 115.38, 115.16, 114.39, 106.73, 106.33, 65.25, 43.08, 40.68. ESI-HRMS [M+H]^+^ *m*/*z*:362.1668, calcd for C_22_H_20_FN_3_O, 362.1663.

1-(2-phenoxyethyl)-3-(2,4,6-trimethylbenzyl)-1,3-dihydro-2H-benzo[*d*]imidazole-2-imine (**5d**). Yellow solid, yield: 88%; M.p. 125.4–127.0 °C. ^1^H NMR (400 MHz, DMSO‑*d*_6_): *δ* 7.35–7.21 (m, 3H), 7.01 (t, *J* = 7.6 Hz, 1H), 6.95–6.76 (m, 6H), 6.32 (d, *J* = 7.9 Hz, 1H), 5.16 (s, 2H), 4.44 (t, *J* = 5.1 Hz, 2H), 4.25 (t, *J* = 5.2 Hz, 2H), 2.28–2.09 (m, 9H). ^13^C NMR (100 MHz, DMSO‑*d*_6_): *δ* ppm 158.03, 151.76, 137.10, 137.09, 131.03, 130.25, 129.56, 129.47, 128.14, 121.36, 121.25, 120.91, 114.31, 108.59, 108.25, 99.51, 65.03, 48.63, 41.74, 41.35, 20.54, 19.79. ESI-HRMS [M+H]^+^ *m*/*z*:386.2227, calcd for C_25_H_27_N_3_O, 386.2227.

### 2.3 Synthesis and characterization of compounds **8a-9b**

#### 2.3.1 Synthesis of **6a** and **6b**

The solution of 2-aminobenzimidazole (15 mmol) in DMF (15 mL) was treated with NaH (19 mmol) and either 2-phenoxyethyl bromide or 2-(bromomethyl)-1,3,5-trimethylbenzene (15 mmol) at 0 °C, then the ice water bath was removed and the reaction solution was stirred at room temperature for another 2h. By monitoring with TLC, the reaction solution was poured into ice water and extracted with EA, followed by collecting the organic phase and washing with brine. After concentration under vacuum, the residue was recrystallized in anhydrous ethanol to yield **6a** and **6b**.

1-(2-phenoxyethyl)-1H-benzo[*d*]imidazole-2-amine (**6a**). White solid, yield: 32%; M.p. 228.1–229.5 °C. ^1^H NMR (400 MHz, DMSO‑*d*_6_): *δ* 7.30–7.18 (m, 3H), 7.11 (dd, *J* = 7.7, 0.9 Hz, 1H), 6.97–6.80 (m, 5H), 6.51–6.37 (m, 2H), 4.38 (t, *J* = 5.5 Hz, 2H), 4.19 (t, *J* = 5.5 Hz, 2H). ^13^C NMR (100 MHz, DMSO‑*d*_6_): *δ* ppm 158.08, 154.98, 142.74, 134.64, 129.57, 120.86, 120.39, 118.09, 114.73, 114.35, 107.98, 65.87, 41.22. ESI-HRMS [M+H]^+^ *m*/*z*:254.1285, calcd for C_15_H_15_N_3_O, 254.1288.

1-(2,4,6-trimethylbenzyl)-1H-benzo[*d*]imidazole-2-amine (**6b**). White solid, yield: 30%; M.p. 269.1–271.0 °C. ^1^H NMR (400 MHz, DMSO‑*d*_6_): *δ* 7.10 (d, *J* = 7.8 Hz, 1H), 6.93–6.78 (m, 3H), 6.71–6.53 (m, 3H), 6.24 (d, *J* = 7.9 Hz, 1H), 5.13 (s, 2H), 2.22 (s, 3H), 2.20–2.11 (m, 6H). ^13^C NMR (100 MHz, DMSO‑*d*_6_): *δ* ppm 155.22, 142.88, 137.02, 136.80, 133.71, 129.32, 128.77, 120.03, 117.82, 114.80, 108.06, 41.81, 20.52, 19.61. ESI-HRMS [M+H]^+^ *m*/*z*:266.1645, calcd for C_17_H_19_N_3_, 266.1652.

#### 2.3.2 Synthesis of **7**

0.4 mmol of various substituted benzyl bromides were added to a solution of compound **6a** or **6b** (0.4 mmol) in acetonitrile. The mixture was stirred at 70 °C for 24 h, and the solvent was then evaporated. The resulting residue was recrystallized with acetone to yield compound **7**.

#### 2.3.3 Synthesis of **8a-9b**

Intermediate **7** was subjected to a reaction with a water solution of KOH in ethyl ether at room temperature for half an hour. After completion of the reaction, the mixture was combined with brine, extracted using diethyl ether, dried using anhydrous Na_2_SO_4,_ and finally concentrated under the vacuum to obtain compound **8a-9b**.

1-(2,4,6-trimethylbenzyl)-1H-benzo[*d*]imidazole-2-amine (**8a**). White solid, yield: 98%; M.p. 273.0–275.0 °C. ^1^H NMR (400 MHz, DMSO‑*d*_6_): *δ* 7.86 (dd, *J* = 9.1, 4.0 Hz, 2H), 7.81–7.73 (m, 2H), 7.50–7.42 (m, 3H), 7.29–7.22 (m, 2H), 7.18 (d, *J* = 7.6 Hz, 1H), 6.99–6.93 (m, 2H), 6.93–6.85 (m, 4H), 5.30 (s, 2H), 4.33 (t, *J* = 5.2 Hz, 2H), 4.26 (t, *J* = 5.1 Hz, 2H). ^13^C NMR (100 MHz, DMSO‑*d*_6_): *δ* ppm 158.18, 152.93, 134.65, 132.76, 132.28, 131.74, 131.10, 129.59, 128.34, 127.62, 126.41, 126.03, 125.69, 125.58, 120.85, 120.66, 120.52, 114.40, 107.46, 107.08, 65.22, 44.41, 41.00. ESI-HRMS [M+H]^+^ *m*/*z*:394.1908, calcd for C_26_H_23_N_3_O, 394.1914.

1-(2-phenoxyethyl)-3-(3-phenoxypropyl)-1,3-dihydro-2H-benzo[*d*]imidazole-2-imine (**8b**). White solid, yield: 95%; M.p. 110.2–112.2 °C. ^1^H NMR (400 MHz, DMSO‑*d*_6_): *δ* 7.30–7.16 (m, 4H), 7.10 (d, *J* = 6.8 Hz, 1H), 6.96 (td, *J* = 7.3, 1.0 Hz, 1H), 6.93–6.80 (m, 8H), 4.28–4.12 (m, 4H), 4.05–3.88 (m, 4H), 2.06 (t, *J* = 6.4 Hz, 2H). ^13^C NMR (100 MHz, DMSO‑*d*_6_): *δ* ppm 158.36, 158.35, 158.14, 158.13, 158.10, 152.74, 131.70, 131.29, 129.58, 129.54, 129.51, 120.81, 120.61, 120.34, 120.28, 114.44, 114.37, 107.16, 106.37, 65.22, 64.59, 40.84, 37.90, 27.25. ESI-HRMS [M+H]^+^ *m*/*z*:388.2016 calcd for C_24_H_25_N_3_O_2_, 388.2020.

1-([1,1′-biphenyl]-4-ylmethyl)-3-(2-phenoxyethyl)-1,3-dihydro-2H-benzo[*d*]imidazole-2-imine (**8c**). White solid, yield: 86%; M.p. 214.1–215.9 °C. ^1^H NMR (400 MHz, DMSO‑*d*_6_): *δ* 7.59 (dd, *J* = 11.7, 7.9 Hz, 4H), 7.43 (t, *J* = 7.5 Hz, 2H), 7.40–7.29 (m, 3H), 7.26 (dd, *J* = 11.7, 4.2 Hz, 2H), 7.07 (d, *J* = 7.9 Hz, 1H), 6.98–6.78 (m, 6H), 5.10 (s, 2H), 4.31–4.14 (m, 4H). ^13^C NMR (100 MHz, DMSO‑*d*_6_): *δ* ppm 158.21, 153.30, 139.85, 139.14, 136.67, 132.01, 131.34, 129.54, 128.93, 127.91, 127.42, 126.83, 126.62, 120.77, 120.07, 119.93, 114.40, 106.72, 106.38, 65.26, 43.49, 40.70. ESI-HRMS [M+H]^+^ *m*/*z*:420.2067, calcd for C_28_H_25_N_3_O, 420.2070.

4-((2-imino-3-(2-phenoxyethyl)-2,3-dihydro-1H-benzo[*d*]imidazole-1-yl)methyl)benzonitrile (**8d**). White solid, yield: 88%; M.p. 135.2–137.0 °C. ^1^H NMR (400 MHz, DMSO‑*d*_6_): *δ* 7.82–7.69 (m, 2H), 7.44 (d, *J* = 8.2 Hz, 2H), 7.31–7.20 (m, 2H), 7.11 (d, *J* = 7.7 Hz, 1H), 6.98–6.90 (m, 2H), 6.90–6.83 (m, 4H), 5.18 (s, 2H), 4.30–4.18 (m, 4H). ^13^C NMR (100 MHz, DMSO‑*d*_6_): *δ* ppm 158.18, 152.99, 143.20, 132.50, 131.90, 131.00, 129.55, 128.11, 120.81, 120.50, 120.24, 118.78, 114.39, 110.06, 107.11, 106.50, 65.25, 43.65, 40.85. ESI-HRMS [M+H]^+^ *m*/*z*:369.1704, calcd for C_23_H_20_N_4_O, 369.1710, found.

1-((benzyloxy)methyl)-3-(2-phenoxyethyl)-1,3-dihydro-2H-benzo[*d*]imidazole-2-imine (**8e**). White solid, yield: 81%; M.p. 149.5–151.2 °C. ^1^H NMR (400 MHz, DMSO‑*d*_6_): *δ* 7.30 (dd, *J* = 7.0, 1.6 Hz, 1H), 7.28–7.19 (m, 6H), 7.07 (d, *J* = 7.3 Hz, 1H), 7.04–7.00 (m, 1H), 6.96 (td, *J* = 7.5, 1.1 Hz, 1H), 6.92–6.82 (m, 4H), 5.39 (s, 2H), 4.55 (s, 2H), 4.25–4.14 (m, 4H). ^13^C NMR (100 MHz, DMSO‑*d*_6_): *δ* ppm 158.18, 153.07, 137.91, 132.07, 130.88, 129.53, 128.22, 127.48, 120.84, 120.77, 120.09, 114.37, 106.94, 70.75, 69.63, 65.19, 40.58. ESI-HRMS [M+H]^+^ *m*/*z*:374.1865, calcd for C_23_H_23_N_3_O_2_, 374.1863.

1-(2-phenoxyethyl)-3-(quinolin-8-ylmethyl)-1,3-dihydro-2H-benzo[*d*]imidazole-2-imine (**8f**). White solid, yield: 91%; M.p. 130.2–132.0 °C. ^1^H NMR (400 MHz, DMSO‑*d*_6_): *δ* 9.04 (dd, *J* = 4.3, 1.8 Hz, 1H), 8.46 (dd, *J* = 8.3, 1.8 Hz, 1H), 7.96 (d, *J* = 7.1 Hz, 1H), 7.66 (dd, *J* = 8.3, 4.2 Hz, 1H), 7.53–7.42 (m, 2H), 7.32 (d, *J* = 6.5 Hz, 1H), 7.29–7.22 (m, 2H), 7.22–7.12 (m, 2H), 7.06 (t, *J* = 7.2 Hz, 1H), 6.92 (t, *J* = 7.2 Hz, 1H), 6.84 (d, *J* = 7.8 Hz, 2H), 5.95–5.84 (m, 2H), 4.54 (t, *J* = 5.3 Hz, 2H), 4.32 (t, *J* = 5.4 Hz, 2H). ^13^C NMR (100 MHz, DMSO‑*d*_6_): *δ* ppm 158.06, 152.09, 150.16, 145.34, 136.78, 133.22, 131.16, 130.68, 129.61, 128.05, 127.94, 126.90, 126.36, 121.99, 121.86, 121.82, 120.97, 114.38, 108.94, 108.43, 65.16, 41.79, 41.72. ESI-HRMS [M+H]^+^ *m*/*z*:395.1865, calcd for C_25_H_22_N_4_O, 395.1866.

1-(anthracen-9-ylmethyl)-3-(2-phenoxyethyl)-1,3-dihydro-2H-benzo[*d*]imidazole-2-imine (**8g**). Yellow solid, yield: 92%; M.p. 186.1–188.0 °C. ^1^H NMR (400 MHz, DMSO‑*d*_6_): *δ* 8.77–8.59 (m, 3H), 8.19–8.04 (m, 2H), 7.60–7.47 (m, 4H), 7.26 (dd, *J* = 8.5, 7.4 Hz, 2H), 7.02–6.84 (m, 4H), 6.70 (t, *J* = 7.6 Hz, 1H), 6.39 (t, *J* = 7.7 Hz, 1H), 6.15–6.03 (m, 2H), 5.94 (d, *J* = 7.8 Hz, 1H), 4.32 (t, *J* = 5.0 Hz, 2H), 4.23 (t, *J* = 5.1 Hz, 2H). ^13^C NMR (100 MHz, DMSO‑*d*_6_): *δ* ppm 158.15, 155.09, 151.93, 131.05, 130.74, 130.35, 130.21, 129.85, 129.76, 129.72, 129.41, 128.74, 127.42, 126.25, 125.61, 125.55, 123.84, 121.66, 121.56, 121.17, 121.04, 120.65, 118.43, 114.50, 109.18, 108.92, 108.24, 66.14, 65.49, 48.79, 41.91, 41.46, 14.56. ESI-HRMS [M+H]^+^ *m*/*z*:444.2070, calcd for C_30_H_25_N_3_O, 444.2070.

1-(2-phenoxyethyl)-3-(4-(trifluoromethyl)benzyl)-1,3-dihydro-2H-benzo[*d*]imidazole-2-imine(**8h**). White solid, yield: 90%; M.p. 105.1–107.0 °C. ^1^H NMR (400 MHz, DMSO‑*d*_6_): *δ* 8.24 (s, 1H), 8.12 (dd, *J* = 8.1, 1.9 Hz, 1H), 7.72 (d, *J* = 7.7 Hz, 1H), 7.60 (t, *J* = 7.9 Hz, 1H), 7.24 (dd, *J* = 8.3, 7.5 Hz, 2H), 7.13 (d, *J* = 7.5 Hz, 1H), 7.01–6.84 (m, 6H), 5.23 (s, 2H), 4.32–4.18 (m, 4H). ^13^C NMR (100 MHz, DMSO‑*d*_6_): *δ* ppm 158.15, 152.90, 147.89, 139.71, 134.04, 131.80, 130.91, 130.18, 129.57, 122.44, 122.26, 120.85, 120.70, 120.46, 114.38, 107.33, 106.73, 65.25, 43.36, 40.91. ESI-HRMS [M+H]^+^ *m*/*z*:412.1634, calcd for C_23_H_20_F_3_N_3_O, 412.1631.

1-(3-nitrobenzyl)-3-(2-phenoxyethyl)-1,3-dihydro-2H-benzo[*d*]imidazole-2-imine (**8i**). Yellow solid, yield: 91%; M.p. 107.1–109.0 °C. ^1^H NMR (400 MHz, DMSO‑*d*_6_): *δ* 7.74 (s, 1H), 7.61 (d, *J* = 7.1 Hz, 1H), 7.57–7.42 (m, 2H), 7.29–7.19 (m, 2H), 7.07 (d, *J* = 7.6 Hz, 1H), 6.93–6.82 (m, 6H), 5.16 (s, 2H), 4.23 (s, 4H). ^13^C NMR (100 MHz, DMSO‑*d*_6_): *δ* ppm 158.20, 153.23, 139.10, 131.96, 131.33, 131.15, 129.67, 129.63, 129.52, 129.36, 129.04, 128.73, 128.24, 125.53, 124.10, 124.06, 124.03, 123.99, 122.82, 120.78, 120.26, 120.12, 120.05, 114.37, 106.85, 106.27, 65.26, 43.39, 40.69. ESI-HRMS [M+H]^+^ *m*/*z*:389.1609, calcd for C_22_H_20_N_4_O_3_, 389.1608.

4'-((2-imino-3-(2-phenoxyethyl)-2,3-dihydro-1H-benzo[*d*]imidazole-1-yl) methyl)-[1,1′-biphenyl]-2-carbonitrile (**8j**). White solid, yield: 88%; M.p. 251.1–253.0 °C. ^1^H NMR (400 MHz, DMSO‑*d*_6_): *δ* 7.94 (d, *J* = 8.2 Hz, 1H), 7.81–7.72 (m, 1H), 7.60–7.51 (m, 4H), 7.42 (d, *J* = 8.2 Hz, 2H), 7.34–7.21 (m, 3H), 7.14 (d, *J* = 7.4 Hz, 1H), 7.10–6.98 (m, 2H), 6.94–6.85 (m, 3H), 5.30 (s, 2H), 4.40 (t, *J* = 5.3 Hz, 2H), 4.28 (t, *J* = 5.3 Hz, 2H). ^13^C NMR (100 MHz, DMSO‑*d*_6_): *δ* ppm 158.07, 152.12, 144.08, 137.02, 133.92, 133.62, 131.25, 130.61, 130.13, 129.59, 129.03, 128.32, 127.42, 121.58, 121.46, 120.92, 118.59, 114.37, 110.12, 108.50, 107.99, 65.11, 44.13, 41.49. ESI-HRMS [M+H]^+^ *m*/*z*:445.2016, calcd for C_29_H_24_N_4_O, 445.2023.

1-(2-(2-ethoxyphenoxy)ethyl)-3-(2-phenoxyethyl)-1,3-dihydro-2H-benzo[*d*]imidazole-2-imine(**8k**). White solid, yield: 84%; M.p. 170.1–171.9 °C. ^1^H NMR (400 MHz, DMSO‑*d*_6_): *δ* 7.23 (dd, *J* = 8.5, 7.4 Hz, 2H), 7.18 (dd, *J* = 6.0, 2.7 Hz, 1H), 7.10 (dd, *J* = 6.2, 2.5 Hz, 1H), 6.99–6.77 (m, 9H), 4.30–4.12 (m, 8H), 3.89 (q, *J* = 7.0 Hz, 2H), 1.25 (t, *J* = 7.0 Hz, 3H). ^13^C NMR (100 MHz, DMSO‑*d*_6_): *δ* ppm 158.11, 152.77, 148.16, 147.95, 131.84, 131.66, 129.52, 121.29, 120.79, 120.72, 120.24, 120.21, 114.36, 113.41, 113.28, 107.48, 106.83, 66.49, 65.24, 63.60, 41.29, 40.80, 14.78. ESI-HRMS [M+H]^+^ *m*/*z*428.2122 calcd for C_25_H_27_N_3_O_3_, 418.2125.

1-phenethyl-3-(2-phenoxyethyl)-1,3-dihydro-2H-benzo[*d*]imidazole-2-imine (**8l**). White solid, yield: 81%; M.p. 117.0–119.0 °C. ^1^H NMR (400 MHz, DMSO‑*d*_6_): *δ* 7.33–7.18 (m, 7H), 7.18–7.13 (m, 2H), 7.10–7.00 (m, 2H), 6.92 (t, *J* = 7.4 Hz, 1H), 6.88–6.79 (m, 2H), 4.37 (t, *J* = 5.3 Hz, 2H), 4.25–4.15 (m, 4H), 2.92 (t, *J* = 8 Hz, 2H). ^13^C NMR (100 MHz, DMSO‑*d*_6_): *δ* ppm 158.02, 151.26, 137.92, 130.91, 130.36, 129.60, 129.07, 128.33, 126.52, 121.53, 121.48, 120.94, 114.34, 108.61, 108.11, 65.08, 42.93, 41.38, 33.14. ESI-HRMS [M+H]^+^ *m*/*z*:358.1918, calcd for C_23_H_23_N_3_O, 358.1914.

1-(3-(benzyloxy)benzyl)-3-(2-phenoxyethyl)-1,3-dihydro-2H-benzo[*d*]imidazole-2-imine (**8m**). White solid, yield: 30.0%. M.p. 145.0–147.0 °C. ^1^H NMR (400 MHz, DMSO‑*d*_6_): *δ* 7.41–7.35 (m, 4H), 7.34–7.30 (m, 1H), 7.27–7.18 (m, 4H), 7.06–6.95 (m, 4H), 6.94–6.89 (m, 2H), 6.88–6.82 (m, 3H), 5.14 (s, 2H), 5.03 (s, 2H), 4.36 (t, *J* = 5.2 Hz, 2H), 4.26 (t, *J* = 5.3 Hz, 2H). ^13^C NMR (100 MHz, DMSO‑*d*_6_): *δ* ppm 158.50, 158.07, 152.35, 138.20, 136.87, 131.31, 130.73, 129.79, 129.55, 128.46, 127.90, 127.83, 121.19, 121.09, 120.88, 119.52, 114.37, 114.13, 113.36, 108.05, 107.69, 69.17, 65.15, 48.16, 44.28, 41.29. ESI-HRMS [M+H]^+^ *m*/*z*:450.2184, calcd for C_29_H_27_N_3_O_2_, 450.2176.

1-(4-methoxybenzyl)-3-(2-phenoxyethyl)-1,3-dihydro-2H-benzo[*d*]imidazole-2-imine (**8n**). White solid, yield: 85%; M.p. 151.4–152.9 °C. ^1^H NMR (400 MHz, DMSO‑*d*_6_): *δ* 7.32–7.21 (m, 5H), 7.12 (d, *J* = 7.5 Hz, 1H), 7.06 (t, *J* = 7.4 Hz, 1H), 7.00 (t, *J* = 7.5 Hz, 1H), 6.91 (t, *J* = 7.3 Hz, 1H), 6.88–6.79 (m, 4H), 5.15 (s, 2H), 4.39 (t, *J* = 5.2 Hz, 2H), 4.25 (t, *J* = 5.2 Hz, 2H), 3.69 (s, 3H). ^13^C NMR (100 MHz, DMSO‑*d*_6_): *δ* ppm 158.67, 158.05, 152.05, 131.21, 130.54, 129.55, 128.86, 128.82, 128.79, 128.78, 128.29, 121.35, 121.29, 120.89, 114.36, 113.98, 108.36, 108.07, 65.16, 55.07, 43.98, 41.40. ESI-HRMS [M+H]^+^ *m*/*z*:374.1869, calcd for C_23_H_23_N_3_O_2_, 374.1863.

1-cinnamyl-3-(2-phenoxyethyl)-1,3-dihydro-2H-benzo[*d*]imidazole-2-imine (**8o**). White solid, yield: 83%; M.p. 160.1–162.0 °C. ^1^H NMR (400 MHz, DMSO‑*d*_6_): *δ* 7.34 (d, *J* = 7.3 Hz, 2H), 7.31–7.19 (m, 5H), 7.16 (d, *J* = 7.1 Hz, 1H), 7.08–7.03 (m, 1H), 7.01–6.93 (m, 2H), 6.93–6.80 (m, 3H), 6.55 (d, *J* = 15.9 Hz, 1H), 6.36–6.22 (m, 1H), 4.74–4.60 (m, 2H), 4.29 (t, *J* = 4.9 Hz, 2H), 4.23 (t, *J* = 4.8 Hz, 2H). ^13^C NMR (100 MHz, DMSO‑*d*_6_): *δ* ppm 158.24, 158.22, 152.59, 136.09, 131.81, 131.79, 131.05, 129.66, 128.77, 127.89, 126.42, 123.96, 120.93, 120.72, 120.66, 114.45, 107.57, 107.17, 65.36, 42.90, 41.07. ESI-HRMS [M+H]^+^ *m*/*z*:370.1915, calcd for C_24_H_23_N_3_O, 370.1914.

1-(3,5-di-*tert*-butylbenzyl)-3-(2-phenoxyethyl)-1,3-dihydro-2H-benzo[*d*]imidazole-2-imine (**8p**). White solid, yield: 91%; M.p. 105.1–107.0 °C. ^1^H NMR (400 MHz, DMSO‑*d*_6_): *δ* 7.23 (t, *J* = 8.0 Hz, 3H), 7.14 (dd, *J* = 11.5, 4.5 Hz, 3H), 7.01 (d, *J* = 7.0 Hz, 1H), 6.97–6.87 (m, 3H), 6.83 (d, *J* = 7.9 Hz, 2H), 5.07 (s, 2H), 4.29 (t, *J* = 5.2 Hz, 2H), 4.22 (t, *J* = 5.1 Hz, 2H), 1.17 (s, 18H). ^13^C NMR (100 MHz, DMSO‑*d*_6_): *δ* ppm 158.16, 153.00, 150.38, 136.24, 131.74, 131.25, 129.52, 121.58, 120.79, 120.74, 120.44, 120.30, 114.32, 107.23, 107.00, 65.25, 44.53, 40.82, 34.40, 31.19. ESI-HRMS [M+H]^+^ *m*/*z*:456.3010, calcd for C_30_H_37_N_3_O, 456.3009.

1-(2-phenoxyethyl)-3-(quinolin-2-ylmethyl)-1,3-dihydro-2H-benzo[*d*]imidazole-2-imine (**8q**). White solid, yield: 87%; M.p. 136.1–138.0 °C. ^1^H NMR (400 MHz, DMSO‑*d*_6_): *δ* 8.26 (d, *J* = 8.6 Hz, 1H), 7.94 (dd, *J* = 13.0, 7.7 Hz, 2H), 7.80–7.69 (m, 1H), 7.64–7.51 (m, 1H), 7.33–7.20 (m, 3H), 7.10 (d, *J* = 7.6 Hz, 1H), 6.98–6.75 (m, 6H), 5.32 (s, 2H), 4.33–4.19 (m, 4H). ^13^C NMR (100 MHz, DMSO‑*d*_6_): *δ* ppm 158.20, 157.50, 153.28, 146.99, 137.10, 132.04, 131.48, 129.82, 129.51, 128.50, 127.87, 126.97, 126.43, 120.76, 120.28, 120.06, 119.34, 114.39, 106.88, 106.45, 65.26, 46.95, 40.79. ESI-HRMS [M+H]^+^ *m*/*z*:395.1869 calcd for C_25_H_22_N_4_O, 395.1866.

5-((2-imino-3-(2-phenoxyethyl)-2,3-dihydro-1H-benzo[*d*]imidazole-1-yl)methyl)picolinonitrile (**8r**). White solid, yield: 90%; M.p. 184.2–186.2 °C. ^1^H NMR (400 MHz, DMSO‑*d*_6_): *δ* 8.84–8.68 (m, 1H), 8.00 (d, *J* = 7.9 Hz, 1H), 7.87 (dd, *J* = 8.1, 2.2 Hz, 1H), 7.40–7.33 (m, 1H), 7.31–7.22 (m, 2H), 7.21 (d, *J* = 7.4 Hz, 1H), 7.13 (dd, *J* = 10.9, 4.2 Hz, 1H), 7.05 (dd, *J* = 7.7, 7.1 Hz, 1H), 6.92 (t, *J* = 7.3 Hz, 1H), 6.84 (d, *J* = 7.9 Hz, 2H), 5.41 (s, 2H), 4.42 (t, *J* = 5.2 Hz, 2H), 4.27 (t, *J* = 5.2 Hz, 2H). ^13^C NMR (100 MHz, DMSO‑*d*_6_): *δ* ppm 158.01, 151.81, 150.38, 136.72, 136.44, 131.66, 131.24, 130.16, 129.58, 129.00, 122.02, 121.75, 120.94, 117.46, 114.33, 108.91, 108.12, 65.16, 42.35, 41.68. ESI-HRMS [M+H]^+^ *m*/*z*:370.1660, calcd for C_22_H_19_N_5_O, 370.1662.

1-(3-phenoxypropyl)-3-(2,4,6-trimethylbenzyl)-1,3-dihydro-2H-benzo[*d*]imidazole-2-imine (**8s**). White solid, yield: 91%; M.p. 184.5–186.4 °C. ^1^H NMR (400 MHz, DMSO‑*d*_6_): *δ* 7.27 (dd, *J* = 8.5, 7.4 Hz, 1H), 7.08 (d, *J* = 7.8 Hz, 1H), 6.94–6.85 (m, 4H), 6.85–6.72 (m, 2H), 6.67–6.54 (m, 1H), 6.49 (s, 2H), 6.23 (d, *J* = 7.8 Hz, 1H), 5.12 (s, 2H), 3.97 (dd, *J* = 14.4, 6.6 Hz, 2H), 2.22 (s, 3H), 2.20 (d, *J* = 2.4 Hz, 4H), 2.16 (s, 6H). ^13^C NMR (100 MHz, DMSO‑*d*_6_): *δ* ppm 158.26, 155.22, 151.75, 142.86, 137.06, 137.02, 136.80, 133.70, 130.83, 130.39, 129.46, 129.42, 129.32, 128.77, 128.23, 121.10, 120.62, 120.03, 117.83, 114.80, 114.39, 108.06, 107.63, 64.41, 41.83, 41.59, 38.45, 27.13, 20.53, 19.77, 19.62. ESI-HRMS [M+H]^+^ *m*/*z*:400.2383, calcd for C_26_H_29_N_3_O, 400.2383.

1-(quinolin-8-ylmethyl)-3-(2,4,6-trimethylbenzyl)-1,3-dihydro-2H-benzo[*d*]imidazole-2-imine (**8t**). White solid, yield: 80%; M.p. 130.1–132.0 °C. ^1^H NMR (400 MHz, DMSO‑*d*_6_): *δ* 9.12–9.00 (m, 1H), 8.43 (d, *J* = 8.2 Hz, 1H), 7.91 (d, *J* = 8.1 Hz, 1H), 7.64 (dd, *J* = 8.3, 4.2 Hz, 1H), 7.51 (t, *J* = 7.5 Hz, 1H), 7.28 (d, *J* = 7.0 Hz, 1H), 6.87 (s, 2H), 6.83–6.42 (m, 3H), 6.22 (d, *J* = 7.8 Hz, 1H), 5.72 (s, 2H), 5.05 (s, 2H), 2.27 (s, 6H), 2.22 (s, 3H). ^13^C NMR (100 MHz, DMSO‑*d*_6_): *δ* ppm 153.51, 150.08, 145.55, 137.08, 136.65, 136.55, 134.63, 131.67, 131.43, 129.36, 128.00, 127.43, 126.52, 126.34, 121.81, 119.95, 119.79, 106.60, 106.32, 40.75, 20.59, 20.03. ESI-HRMS [M+H]^+^ *m*/*z*:407.2229, calcd for C_27_H_26_N_4_, 407.2230.

1-(3-nitrobenzyl)-3-(2,4,6-trimethylbenzyl)-1,3-dihydro-2H-benzo[*d*]imidazole-2-imine (**8u**). Yellow solid, yield: 82%; M.p. 193.0–194.8 °C. ^1^H NMR (400 MHz, DMSO‑*d*_6_): *δ* 8.25–8.06 (m, 2H), 7.87–7.59 (m, 2H), 6.95–6.82 (m, 3H), 6.80–6.62 (m, 2H), 6.35–6.08 (m, 1H), 5.20 (s, 2H), 5.04 (s, 2H), 2.27–2.19 (m, 9H). ^13^C NMR (100 MHz, DMSO‑*d*_6_): *δ* ppm 153.11, 147.87, 140.07, 136.95, 136.54, 134.00, 131.25, 130.99, 130.08, 129.33, 122.28, 121.85, 120.17, 119.84, 106.67, 106.27, 64.95, 43.09, 40.70, 20.50, 19.90. ESI- ESI-HRMS [M+H]^+^ *m*/*z*:401.1976, calcd for C_24_H_24_N_4_O_2_, 401.1972.

1-([1,1′-biphenyl]-4-ylmethyl)-3-(2,4,6-trimethylbenzyl)-1,3-dihydro-2H-benzo[*d*]imidazole-2-imine (**8v**). White solid, yield: 84%; M.p. 164.0–166.0 °C. ^1^H NMR (400 MHz, DMSO‑*d*_6_): *δ* 7.65 (t, *J* = 8.0 Hz, 4H), 7.45 (t, *J* = 7.6 Hz, 2H), 7.42–7.31 (m, 3H), 7.24 (d, *J* = 7.8 Hz, 1H), 7.01 (t, *J* = 7.7 Hz, 1H), 6.96–6.84 (m, 3H), 6.37 (d, *J* = 8.0 Hz, 1H), 5.39 (s, 2H), 5.25 (s, 2H), 2.29–2.18 (m, 9H). ^13^C NMR (100 MHz, DMSO‑*d*_6_): *δ* ppm 151.42, 139.62, 137.36, 137.20, 135.12, 130.45, 130.21, 129.57, 129.01, 127.84, 127.80, 127.62, 127.03, 126.71, 122.07, 121.86, 108.95, 44.53, 42.22, 20.60, 19.86. ESI-HRMS [M+H]^+^ *m*/*z*:432.2441, calcd for C_30_H_29_N_3_, 432.2434.

4'-((2-imino-3-(2,4,6-trimethylbenzyl)-2,3-dihydro-1H-benzo[*d*]imidazole-1-yl)methyl)-[1,1′-biphenyl]-2-carbonitrile (**8w**). White solid, yield: 88%; M.p. 135.0–136.8 °C. ^1^H NMR (400 MHz, DMSO‑*d*_6_): *δ* 7.90 (dd, *J* = 7.8, 1.1 Hz, 1H), 7.73 (td, *J* = 7.7, 1.3 Hz, 1H), 7.58–7.48 (m, 4H), 7.40 (d, *J* = 8.2 Hz, 2H), 6.88 (t, *J* = 6.0 Hz, 1H), 6.82 (s, 2H), 6.74 (td, *J* = 7.7, 0.9 Hz, 1H), 6.63 (td, *J* = 7.7, 1.0 Hz, 1H), 6.22 (d, *J* = 7.8 Hz, 1H), 5.14 (s, 2H), 5.01 (s, 2H), 2.23–2.15 (m, 9H). ^13^C NMR (100 MHz, DMSO‑*d*_6_): *δ* ppm 153.71, 144.68, 138.66, 137.52, 137.23, 137.02, 134.39, 134.07, 131.81, 131.75, 130.64, 129.81, 129.76, 129.39, 128.73, 128.00, 120.53, 120.28, 119.11, 110.62, 107.16, 106.93, 65.47, 43.95, 41.22, 21.04, 20.47. ESI-HRMS [M+H]^+^ *m*/*z*:457.2386, calcd for C_31_H_28_N_4_, 457.2387.

1-(3,5-di-*tert*-butylbenzyl)-3-(2,4,6-trimethylbenzyl)-1,3-dihydro-2H-benzo[*d*]imidazole-2-imine (**8x**). White solid, yield: 89%; M.p. 177.0–179.0 °C. ^1^H NMR (400 MHz, DMSO‑*d*_6_): *δ* 7.26 (t, *J* = 1.7 Hz, 1H), 7.23–7.11 (m, 2H), 6.96–6.75 (m, 4H), 6.67 (t, *J* = 7.7 Hz, 1H), 6.23 (d, *J* = 7.6 Hz, 1H), 5.07 (s, 2H), 5.05 (s, 2H), 2.26–2.17 (m, 9H), 1.22 (s, 18H). ^13^C NMR (100 MHz, DMSO‑*d*_6_): *δ* ppm 153.20, 150.38, 136.91, 136.64, 136.55, 131.41, 131.05, 129.37, 129.24, 121.43, 120.74, 119.96, 119.76, 106.58, 44.35, 40.72, 34.46, 31.24, 20.54, 20.04. ESI-HRMS [M+H]^+^ *m*/*z*:468.3369, calcd for C_32_H_41_N_3_, 468.3373.

1-(naphthalen-1-ylmethyl)-3-(2,4,6-trimethylbenzyl)-1,3-dihydro-2H-benzo[*d*]imidazole-2-imine (**8y**). White solid, yield: 90%; M.p. 281.7–283.0 °C. ^1^H NMR (400 MHz, DMSO‑*d*_6_): *δ* 8.26 (d, *J* = 7.9 Hz, 1H), 8.05–7.96 (m, 1H), 7.88 (d, *J* = 8.2 Hz, 1H), 7.69–7.56 (m, 2H), 7.46–7.34 (m, 1H), 7.05–6.91 (m, 4H), 6.90–6.77 (m, 2H), 6.41–6.31 (m, 1H), 5.76 (s, 2H), 5.23 (s, 2H), 2.34–2.21 (m, 9H). ^13^C NMR (100 MHz, DMSO‑*d*_6_): *δ* ppm 151.84, 137.24, 137.20, 133.45, 131.22, 130.93, 130.54, 130.49, 129.56, 128.68, 128.16, 127.96, 126.46, 126.27, 125.40, 123.63, 123.02, 121.61, 121.39, 119.00, 108.49, 108.44, 99.53, 65.01, 43.42, 41.94, 20.61, 19.94. ESI-HRMS [M+H]^+^ *m*/*z*:406.2283, calcd for C_28_H_27_N_3_, 406.2278.

1-cinnamyl-3-(2,4,6-trimethylbenzyl)-1,3-dihydro-2H-benzo[*d*]imidazole-2-imine (**8z**). White solid, yield: 91%; M.p. 260.1–262.0 °C. ^1^H NMR (400 MHz, DMSO‑*d*_6_): *δ* 7.39 (dd, *J* = 7.6, 2.6 Hz, 3H), 7.33 (t, *J* = 7.5 Hz, 2H), 7.25 (t, *J* = 7.2 Hz, 1H), 7.09 (t, *J* = 7.7 Hz, 1H), 6.98–6.88 (m, 3H), 6.61 (d, *J* = 16.0 Hz, 1H), 6.42–6.30 (m, 2H), 5.30 (s, 2H), 4.96 (d, *J* = 5.4 Hz, 2H), 2.27–2.19 (m, 9H). ^13^C NMR (100 MHz, DMSO‑*d*_6_): *δ* ppm 158.51, 150.64, 137.54, 137.42, 137.19, 137.09, 136.83, 135.78, 132.12, 130.20, 129.97, 129.53, 128.70, 128.43, 127.97, 127.88, 127.74, 127.53, 126.35, 122.74, 122.34, 122.21, 109.37, 43.67, 42.56, 20.53, 20.51, 19.77, 19.73. ESI-HRMS [M+H]^+^ *m*/*z*:382.2286 calcd for C_26_H_27_N_3_, 382.2278.

1-(naphthalen-2-ylmethyl)-3-(2,4,6-trimethylbenzyl)-1,3-dihydro-2H-benzo[*d*]imidazole-2-imine (**9a**). White solid, yield: 85%; M.p. 140.5–141.9 °C. ^1^H NMR (400 MHz, DMSO‑*d*_6_): *δ* 7.89 (dd, *J* = 9.0, 4.4 Hz, 2H), 7.85–7.76 (m, 2H), 7.56–7.48 (m, 2H), 7.44 (dd, *J* = 8.5, 1.6 Hz, 1H), 7.03 (d, *J* = 7.7 Hz, 1H), 6.89 (s, 2H), 6.84 (t, *J* = 7.7 Hz, 1H), 6.78–6.70 (m, 1H), 6.30 (d, *J* = 7.8 Hz, 1H), 5.36 (s, 2H), 5.14 (s, 2H), 2.30–2.17 (m, 9H). ^13^C NMR (100 MHz, DMSO‑*d*_6_): *δ* ppm 152.46, 137.02, 136.86, 134.29, 132.72, 132.28, 130.91, 130.75, 129.41, 128.55, 128.32, 127.60, 127.52, 126.46, 126.04, 125.55, 125.35, 120.89, 120.67, 107.65, 107.53, 64.92, 44.52, 41.39, 20.51, 19.86, 15.17. ESI-HRMS [M+H]^+^ *m*/*z*:406.2275, calcd for C_28_H_27_N_3_, 406.2278.

1-(3-(benzyloxy)benzyl)-3-(2,4,6-trimethylbenzyl)-1,3-dihydro-2H-benzo[*d*]imidazole-2-imine (**9b**). White solid, yield: 87%; M.p. 117.9–119.9 °C. ^1^H NMR (400 MHz, DMSO‑*d*_6_): *δ* 7.44–7.29 (m, 5H), 7.24 (t, *J* = 7.9 Hz, 1H), 6.97–6.76 (m, 7H), 6.71 (t, *J* = 7.7 Hz, 1H), 6.27 (d, *J* = 7.7 Hz, 1H), 5.16–4.96 (m, 6H), 2.27–2.13 (m, 9H). ^13^C NMR (100 MHz, DMSO‑*d*_6_): *δ* ppm 158.48, 153.25, 139.11, 136.98, 136.51, 131.29, 131.20, 129.63, 129.31, 128.46, 127.89, 127.77, 119.94, 119.72, 119.57, 114.02, 113.13, 106.57, 106.44, 69.13, 43.71, 40.70, 20.52, 19.95. ESI-HRMS [M+H]^+^ *m*/*z*:462.2536, calcd for C_31_H_31_N_3_O, 462.2540.

### 2.4 Cell culture

In this study, human myeloma cells (RPMI-8226 and U266) and human renal epithelial cells (293T) were obtained from the Jiangsu Key Laboratory of New Drug Research and Clinical Pharmacy. All the cells were cultured in RPMI-1640 medium (KeyGEN BioTECH) containing 10% fetal bovine serum (ZETA LIFE) at 37 °C with 5% CO_2_ in the incubator.

### 2.5 Cell viability assay

Human myeloma cells (RPMI-8226 and U266) and human renal epithelial cells (293T) were seeded in a 96-well plate at a density of 1 × 10^4^ cells per well, respectively. Following incubation with synthesized compounds at varying concentrations, the cell survival rate was determined using a CCK-8 kit (KeyGEN BioTECH, China) by monitoring absorbance at 450 nm according to the manufacturer's instructions.

### 2.6 Western blot and Q-PCR assay

Following the cultivation of RPMI-8226 and U266 cells under specific conditions, treatment with varying concentrations of compounds **5b**, **5d**, and **8g** was administered. The resulting cells were first collected, and treated with a mixture of proteasome inhibitor and RIPA lysis solution to prepare protein samples. The samples were electrophoresed by SDS-PAGE, which was transferred to the PVDF membrane. After the block with 5% skimmed milk, the membrane was treated with corresponding primary as well as secondary antibodies, successively. The final result was recorded with a chemiluminescence system. Total RNA was extracted using Trizol reagent (Takara) and reverse-transcribed to complementary DNA using a Reverse Transcription Kit (Vazyme). Q-PCR assays were performed on a Roche 480 instrument, and the results were analyzed using the 2^-△△CT^ method.

### 2.7 Dual-luciferase reporter assay

After seeding 293T cells in a 6-well plate and cultivating them at 5% CO_2_ and 37 °C overnight, the cells were transfected with luciferase plasmid when their growth density reached 80%. Six hours later, compounds **5b** and **5d** were added to the cells followed by another incubation for 24 h. The luciferase activity was measured using the Dual-Lumi™ II Luciferase Reporter Gene Assay Kit (Beyotime Biotechnology).

### 2.8 Cell apoptosis assay

The RPMI-8226 and U266 cells were seeded in a 12-well plate with 15,000 cells per well. After treatment with compounds **5b** and **5d** for 48 h, the cells were resuspended in PBS and Binding-Buffer before being stained with APC and 7AAD. Apoptotic cells were then quantified using flow cytometry (BD Biosciences) and analyzed using Flowjo-10 software.

### 2.9 Cell arrest assay

After incubating RPMI-8226 and U266 cells (15,000 cells per well) in a 12-well plate and treating them with compounds **5b** and **5d**, the cells were resuspended in PBS, fixed with 75% alcohol, and then resuspended again in 1000 μL of PBS. Subsequently, the cells were treated with Binding Buffer (100 μL), RNase-A (1 μL), and incubated at final concentrations of 100 μg/mL and 50 μg/mL respectively. The cell cycle distribution was analyzed using a flow cytometer (BD Biosciences), and the final results were obtained through MODFIT Software.

### 2.10 Molecular docking studies

The Surflex Dock of Sybyl-X2.1 software was employed to investigate the binding modes between compounds **5b** and **5d** with c-Myc/Max based on the crystal structure of the c-Myc/Max complex (PDB ID: 1NKP). The binding site for ligands was identified as a well-defined pocket formed by specific residues in both c-Myc (Leu917, Phe921, and Lys939) and Max (Arg 212, Arg215, Asp 216, Ile218, Lys 219, Phe222, and Arg 239). Before docking simulations, hydrogen atoms missing from the protein structures were added using the biopolymer module while all water molecules were excluded. The default docking parameters were configured, and the binding affinities of two compounds were assessed based on the docking score expressed in -log (Kd) units.

### 2.11 Drug-likeness and ADME profiling

The resulting compounds **5b** and **5d** underwent In silico drug-likeness and pharmacokinetic evaluation using the online tool "SwissADME" (http://www.swissadme.ch/) to determine their endpoint parameters.

### 2.12 Statistical analysis

The experimental data were presented as mean ± SD, and statistical analysis was performed using GraphPad Prism 5.0 with the student's t-test and ANOVA. A *p*-value <0.05 was considered statistically significant.

## 3 Results and discussion

### 3.1 Synthesis of **5a-d**, **8a-9b** series derivatives

**Scheme 1:**
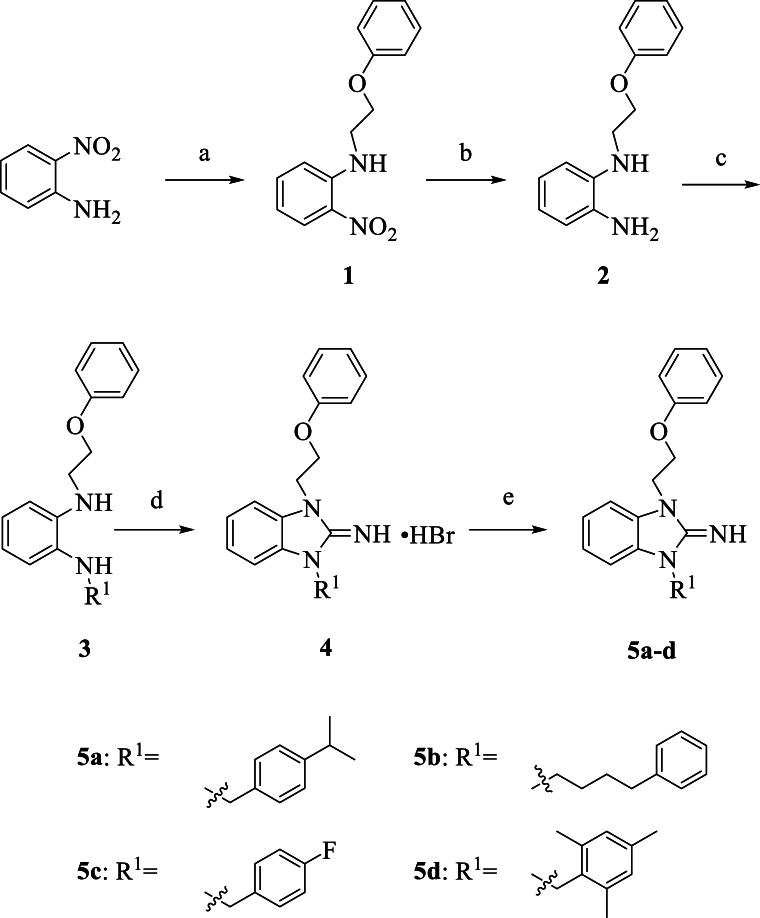
Synthetic process of compounds **5a-d**. Reagents and conditions: (a) 2-bromoethoxy benzene, NaH, DMF, reflux; (b) NH_4_Cl, Fe, THF, 60 °C; (c) various benzyl bromide, K_2_CO_3_, DMF, 120 °C; (d) BrCN, MeOH, CH_2_Cl_2_, rf; (e) KOH, Ethyl ether, rt.

**Scheme 2:**
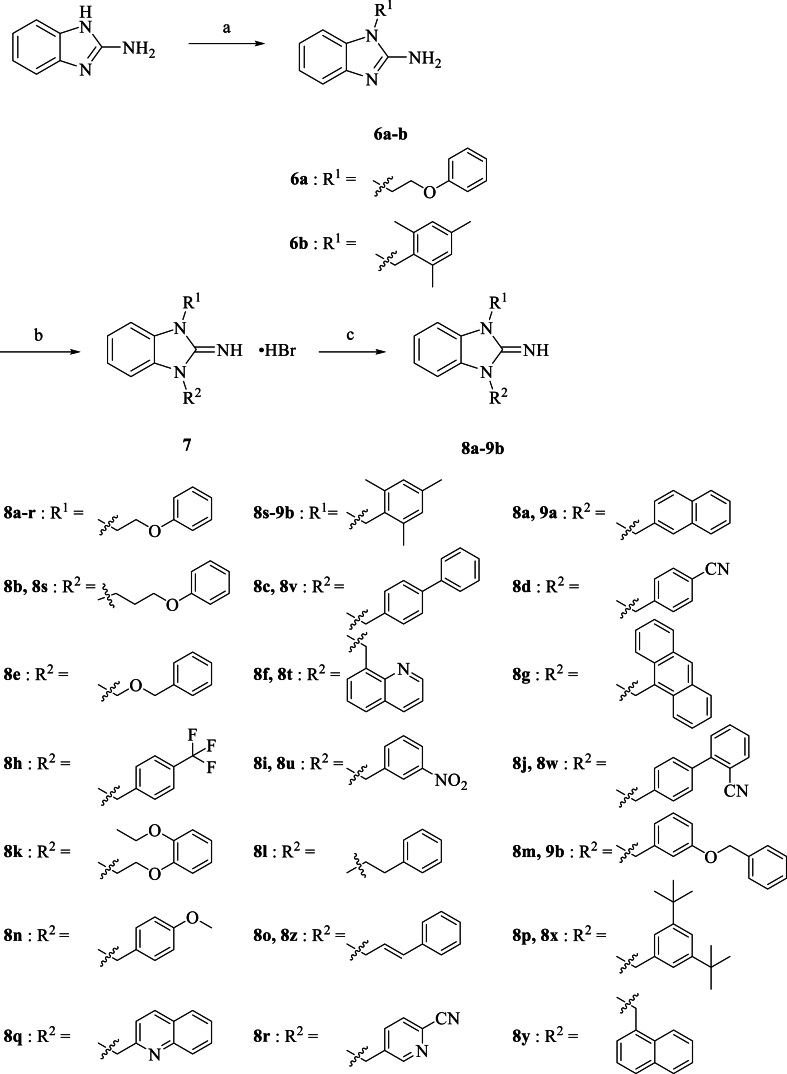
Synthetic process of compounds **8a-9b**. Reagents and conditions: (a) (2-bromoethoxy) benzene/2-(bromomethyl)-1,3,5-trimethylbenzene, NaH, DMF, rt; (b) various benzyl bromide, CH_3_CN, 70 °C; (c) KOH, Ethyl ether, rt.

The synthetic procedures for compounds **5a-d** and **8a-9b** are depicted in Scheme 1 and Scheme 2, respectively. Initially, 2-nitroaniline was reacted with 2-phenoxyethyl bromide under the alkaline condition using NaH via nucleophilic substitution reaction to yield compound **1**, which was subsequently reduced by means of iron powder to produce compound **2**. Compound **3** was obtained by nucleophilic substitution reaction using K_2_CO_3_ of compound **2** with various substituted benzyl bromides, respectively. After treatment of compound **3** with BrCN, the ring closed and formed the key structure of 2-iminobenzimidazoles. Finally, compound **4** was combined with aqueous solution of KOH to give compound **5a-d**.

Afterward, we changed the synthetic routes, and the most of target compounds followed Scheme 2. Firstly, the starting material 2-aminobenzimidazole was reacted with 2-phenoxyethyl bromide or 2-(bromomethyl)-1,3,5-trimethylbenzene via nucleophilic substitution reaction using NaH as catalyst to afford compound **6a-b**. Then, the monosubstituted compound **6a-b** were treated with various substituted benzyl bromides via another nucleophilic substitution reaction in the CH_3_CN solution at 70 °C to form disubstituted compound **7**, respectively, which constructed the core structure of 2-iminobenzimidazoles. Ultimately, compound **7** was stirred with KOH water solution to attain compound **8a-9d**.

### 3.2 Cytotoxicity of the compounds **5a-d** and **8a-9b**

The cytotoxicity of the target compounds against human myeloma cells (RPMI-8226 and U266) and human renal epithelial cells (293T), was assessed using CCK-8 assay. As presented in Table 1, most of the tested compounds demonstrated significant inhibitory effects with IC_50_ values below 5 μM, which further confirms that the core structure of 2-iminobenzimidazole is crucial for exerting anti-MM activity. Moreover, a preliminary analysis of the structure-activity relationship suggested that when R^1^ undergoes single displacement and R^2^ is H, compounds **6a** and **6b** exhibited weaker activity (IC_50_ above 5 μM) compared to other compounds. When R^1^ was ethoxybenzene and R^2^ was varied to **5a-d** and **8a-r**, the IC_50_ values ranged from approximately 1 μM–5 μM. Notably, compounds **5b**, **5d**, and **8g** exhibited remarkable activity with IC_50_ values below 1 μM (R^2^ = propylbenzene for compound 5b; R^2^ = 2-methyl-1,3,5-trimethylbenzene for compound **5d**; and R^2^ = 9-methylanthracene for compound **8g**), which were more potent than the lead compound XYA1353 (IC_50_ about 2 μM [36]).

**Table 1:**
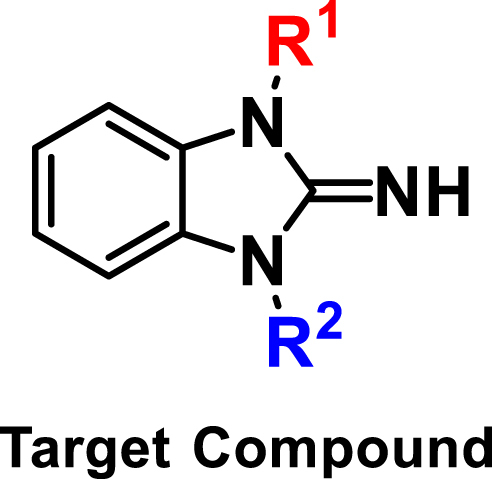
Inhibitory effect of **5a-d** and **8a-9b** on RPMI-8226 and U266 cell lines.

Compounds	R^1^	R^2^	IC_50_(μM)^a^ RPMI-8226	IC_50_ (μM)^a^ U266
**5a**			2.22 ± 0.21	2.45 ± 0.08
**5b**			0.85 ± 0.14	0.97 ± 0.11
**5c**			4.35 ± 0.48	4.15 ± 0.24
**5d**			0.96 ± 0.15	0.89 ± 0.09
**6a**		**H**	5.14 ± 0.24	4.96 ± 0.58
6d		**H**	4.93 ± 0.34	5.24 ± 0.33
**8a**			2.18 ± 0.41	2.44 ± 0.89
**8b**			4.98 ± 0.56	4.26 ± 1.56
**8c**			4.95 ± 0.12	4.70 ± 1.11
**8d**			4.56 ± 0.11	4.23 ± 1.35
**8e**			2.85 ± 0.44	2.65 ± 0.88
**8f**			1.32 ± 0.55	1.45 ± 0.12
**8g**			0.98 ± 0.10	1.01 ± 0.09
**8h**			2.92 ± 0.48	2.66 ± 0.17
**8i**			2.22 ± 0.17	1.98 ± 0.28
**8j**			2.56 ± 0.29	2.77 ± 0.46
**8k**			1.99 ± 0.07	1.76 ± 0.77
**8l**			5.13 ± 0.44	5.44 ± 0.59
**8m**			2.40 ± 0.59	2.67 ± 0.88
**8n**			2.03 ± 0.27	1.88 ± 0.44
**8o**			1.99 ± 0.39	2.66 ± 0.98
**8p**			1.04 ± 0.11	1.44 ± 0.44
**8q**			1.18 ± 0.79	1.05 ± 0.89
**8r**			1.11 ± 0.26	1.55 ± 0.16
**8s**			1.87 ± 0.36	1.66 ± 0.05
**8t**			2.05 ± 0.58	1.99 ± 0.49
**8u**			2.45 ± 0.84	2.63 ± 0.33
**8v**			2.32 ± 0.54	2.20 ± 0.29
**8w**			2.21 ± 0.28	2.46 ± 0.37
**8x**			1.99 ± 0.07	2.23 ± 0.24
**8y**			1.27 ± 0.53	1.34 ± 0.57
**8z**			1.02 ± 0.07	1.24 ± 0.48
**9a**			1.88 ± 0.12	1.59 ± 0.19
**9b**			1.76 ± 0.19	1.98 ± 0.42

a IC_50_, the mean ± SD of 3 independent experiments.

Furthermore, the substitution of R^1^ with 2-methyl-1,3,5-trimethylbenzene and variation of R^2^ resulted in an average IC_50_ value of approximately 2 μM for compound **8s-9b**. This suggests that compounds containing the 2-methyl-1,3,5-trimethylbenzene moiety on R^1^ exhibit superior activity compared to those with ethoxybenzene on R^1^. More importantly, compounds **5b**, **5d,** and **8g** showed negligible intrinsic cytotoxicity (IC_50_ > 90 μM), thus indicating their favorable safety profiles (Table S1). Therefore, compounds **5b**, **5d,** and **8g** (all exhibiting IC_50_ values below 1 μM) were selected for further evaluation of their anti-MM activity.

### 3.3 Compounds **5b** and **5d** exerted inhibitory effects on the expression and transcriptional activity of c-Myc

**Fig. 2:**
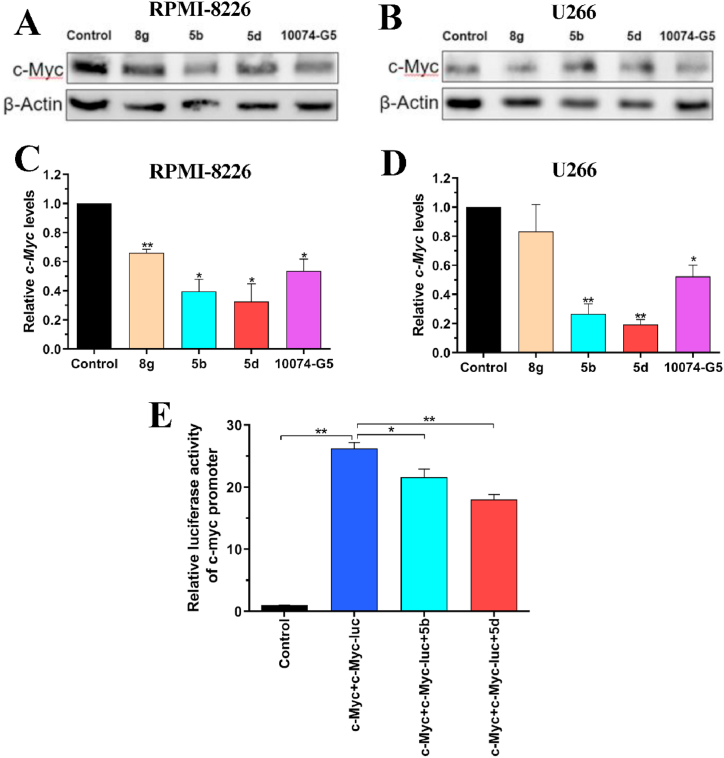
(A–B) Western blot analysis of RPMI-8226 and U266 cells treated with 1 μM **8g**, **5b,** and **5d**. The positive control used was 10074-G5 (The uncropped versions were shown in Fig. S1). (C–D) Q-PCR analysis of c-Myc mRNA levels in RPMI-8226 and U266 cells treated with 1 μM of compounds **8g**, **5b**, and **5d**, as well as with 15 μM of compound 10074-G5. (E) Dual-luciferase reporter assay analysis of c-Myc's promoter activity affected by **5b** and **5d**. Error bars represent mean ± SD (n = 3). *P < 0.05, **P < 0.01.

Compounds **5b**, **5d**, and **8g** were selected for Western blot analysis due to their apparent ability to suppress two MM cells. As one of the earliest inhibitors of c-Myc, 10074-G5 has been extensively utilized in investigating anti-tumor mechanisms and developing its derivatives. In this study, 10074-G5 was employed as a positive control. As depicted in Fig. 2A and B, compared to the control group, treatment with compounds **5b**, **5d**, and 10074-G5 resulted in decreased c-Myc protein expression in RPMI-8226 and U266 cells; however, compound **8g** exhibited poor efficacy against U266 cells. Notably, both cell lines treated with compounds **5b** and **5d** demonstrated a more pronounced reduction in c-Myc protein expression than those treated with the positive control. Meanwhile, our Q-PCR assay revealed a significant reduction in the mRNA level of c-Myc gene upon treatment with compounds **5b** and **5d** in RPMI-8226 and U266 cells. (Fig. 2C and D).

Subsequently, we further evaluated the impact of compounds **5b** and **5d** on c-Myc transcriptional activity based on dual-luciferase reporter assay. It has been reported that c-Myc acts as a positive master regulator by binding to the conserved DNA sequence CACGTG (E-box motif) and further transcriptionally activating downstream target genes. In this study, we constructed a luciferase reporter gene plasmid by cloning multiple copies of the E-box motif. As illustrated in Fig. 2E, c-Myc significantly enhanced the transcriptional activity of its target genes. Conversely, compounds **5b** and **5d** exhibited inhibitory effects on c-Myc-mediated transcriptional activities, with compound **5d** demonstrating superior efficacy. In summary, both compounds selectively targeted the c-Myc protein and exerted potent anti-MM effects.

### 3.4 Molecular modeling analysis

**Fig. 3:**
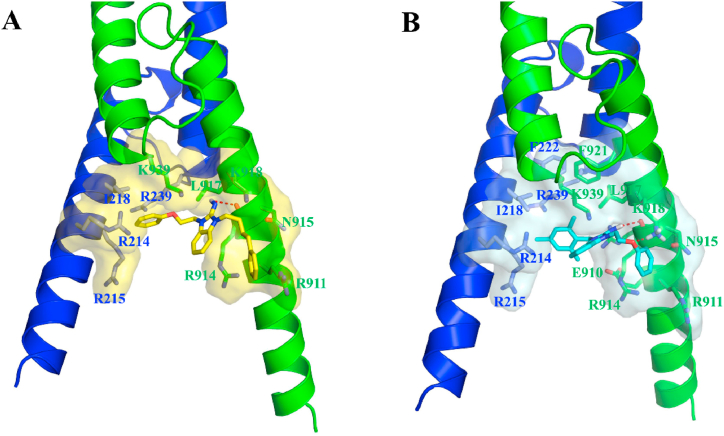
Predicted binding modes of **5b** (A) and **5d** (B) in the interface of c-Myc/Max to E-box of DNA. Proteins c-Myc and Max are represented as a cartoon, colored green and blue, respectively. Compounds **5b** (A) and **5d** (B) are displayed as sticks with yellow and cyan carbon atoms, respectively. Hydrogen bonds are indicated by a red dashed line. (For interpretation of the references to color in this figure legend, the reader is referred to the Web version of this article.)

To predict the potential binding mode of compounds **5b** and **5d** with c-Myc, we proceeded to conduct a molecular docking study utilizing Sybyl-X2.1 software (Tripos Inc., USA). Considering the inhibitory effect of compounds **5b** and **5d** on c-Myc-mediated transcriptional activity, as demonstrated by dual-luciferase reporter assay, these two compounds were subjected to docking analysis at the interface between c-Myc/Max and E-box of DNA. As shown in Fig. 3A and B, both **5b** and **5d** were snugly embedded within the hydrophobic pocket, which is composed of residues Arg 214, Arg215, Ile218, and Phe222 in Max as well as Arg 911, Leu917, Phe921, and Lys939 in c-Myc. Additionally, **5b** formed one hydrogen bond with the main chain of Arg914 while **5d** had two hydrogen bonds with the main chain of Arg914 and the side chain of Lys 918. Thus, the stronger hydrophobic and hydrogen bond interactions established between c-Myc/Max and compounds **5b** and **5d** would disrupt the binding of c-Myc/Max to the E-box of DNA.

### 3.5 Compounds **5b** and **5d** induced apoptosis and G1 cell cycle arrest

**Fig. 4:**
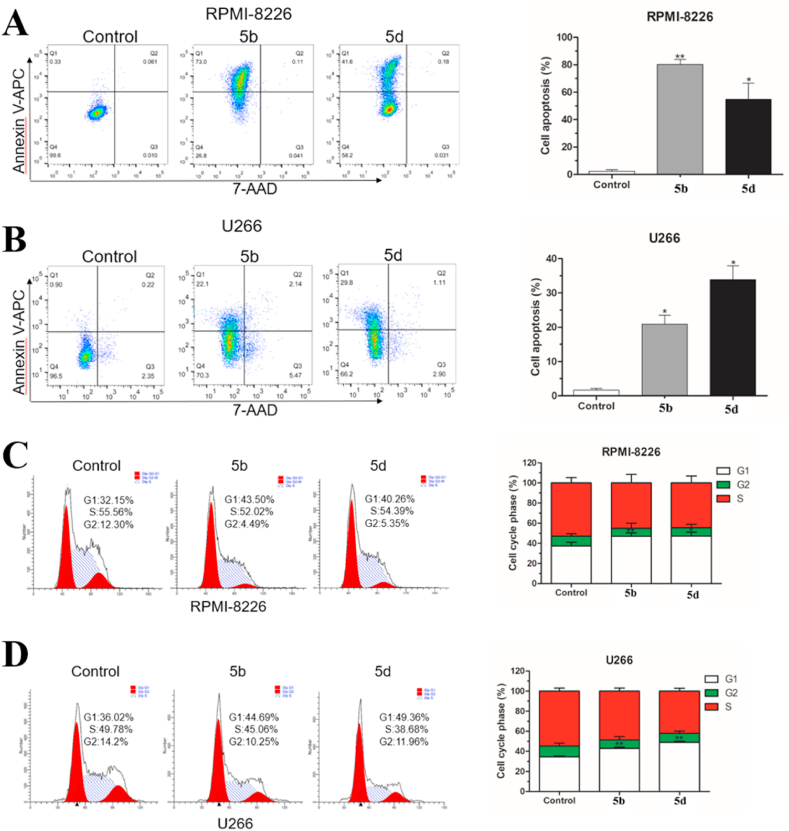
Effects of **5b** and **5d** on apoptosis (A and B) and cell cycle distribution (C and D) in RPMI-8226 and U266 cells. Error bars represent mean ± SD (n = 3). *P < 0.05, **P < 0.01.

In light of the fact that lead compound XYA1353 has been shown to induce apoptosis in MM cells by activating the caspase-dependent endogenous pathway [36], flow cytometry was performed on compounds **5b** and **5d** at a concentration of 1 μM to evaluate their effects on cell apoptosis in RPMI-8226 and U266 cells. The results depicted in Fig. 4A and B indicate that, compared to the control group, compounds **5b** and **5d** significantly induced apoptosis in two MM cell lines. Specifically, RPMI-8226 cells treated with **5b** and **5d** exhibited an apoptotic rate of approximately 80% and 50%, respectively, while U266 cells showed rates exceeding 20% and 35%. Next, we proceeded to evaluate the impact of **5b** and **5d** on cell cycle distribution. Our findings revealed that both compounds significantly increased the percentage of cells in the G1 phase. In detail, RPMI-8226 cells treated with **5b** exhibited a more pronounced increase while U266 cells treated with **5d** displayed a similar trend (as shown in Fig. 4C and D). Similar to the lead compound XYA1353, it will be necessary to assess the impact of compounds **5b** and **5d** on apoptosis-associated markers such as Bak, Bax, and PARP1 in future evaluations.

### 3.6 Drug likeness and ADME analysis

It is well known that knowledge of the physiochemical properties and ADME profiles of hit/lead compounds is valuable in early drug discovery, reducing the risk of failure in later stages of development. The drug likeness and ADME analysis of compounds **5b** and **5d** were carried out by the free web tool “SwissADME” (http://www.swissadme.ch/) and the results were shown in Table 2. The table presents crucial data, including the count of heavy atoms, H-bond donors, and acceptors. Moreover, essential molecular properties such as the number of rotatable bonds, molar refractivity (MR), and Topological Polar Surface Area (TPSA) were computed.

**Table 2:** Calculated physicochemical and ADME properties of compounds **5b** and **5d**.

Property	5b	5d
Molecular weight	385.50 g/mol	385.50 g/mol
Num. heavy atoms	29	29
Num. arom. heavy atoms	21	21
Fraction Csp 3	0.24	0.24
Num. rotatable bonds	9	6
Num. H-bond acceptors	2	2
Num. H-bond donors	1	1
Molar Refractivity	118.71	119.18
TPSA	42.94 Å^2^	42.94 Å^2^
GI absorption	High	High
BBB permeant	Yes	Yes
P-gp substrate	Yes	Yes
CYP1A2 inhibitor	Yes	Yes
CYP2C19 inhibitor	Yes	Yes
CYP2C9 inhibitor	Yes	Yes
CYP2D6 inhibitor	Yes	Yes
CYP3A4 inhibitor	Yes	Yes
Log *K*_p_ (skin permeation)	−5.02 cm/s	−4.91 cm/s
Lipinski	Yes; 1 violation: MLOGP>4.15	Yes; 1 violation: MLOGP>4.15
Ghose	Yes	Yes
Veber	Yes	Yes
Egan	Yes	Yes
Muegge	No; 1 violation: XLOGP3>5	No; 1 violation: XLOGP3>5
Bioavailability Score	0.55	0.55

The physicochemical properties of compounds **5b** and **5d** were identical, except for the difference in the number of rotatable bonds. Additionally, both compounds exhibited high gastrointestinal (GI) absorption (as per the Boiled egg), and they also demonstrated BBB permeability. In terms of drug likeness, compounds **5b** and **5d** were found to be compliant with Lipinski, Ghose, Veber, and Egan criteria, with a bioavailability score of 0.55.

## 4 Conclusion

The present study involves the synthesis and evaluation of a series of novel 2-iminobenzimidazole derivatives as potential anti-multiple myeloma (MM) agents, building upon the lead compound XYA1353. According to the CCK-8 results, compounds **5b**, **5d**, and **8g** demonstrated superior cytotoxicity against RPMI-8226 and U266 cells among the 34 tested compounds. Moreover, upon substitution of ethoxybenzene with 2-methyl-1,3,5-trimethylbenzene on the R^1^ side chain (compound **8s-9b**), their inhibitory activity surpassed that of aforementioned compounds with an average IC_50_ value of approximately 2 μM.

The Western blot and Dual-luciferase reporter assay results demonstrated that compounds **5b** and **5d** exhibited the ability to reduce c-Myc protein expression, which was likely due to their suppressive effects on c-Myc promoter transcriptional activity. Furthermore, compounds **5b** and **5d** were capable of inducing apoptosis and G1 phase cell cycle arrest in RPMI-8226 and U266 cells. Therefore, owing to their groundbreaking chemical structure and extraordinary anti-MM activity, 2-iminobenzimidazoles (such as compounds **5b** and **5d**) can be identified as novel potent c-Myc inhibitors for the treatment of multiple myeloma. Future research should prioritize the investigation of the underlying molecular mechanism against c-Myc-related diseases, both in *vitro* and in *vivo*.

## Funding

This research was funded by the Natural Science Research Project of Anhui Educational Committee (grant number: 2023AH051226); 10.13039/501100003995Anhui Provincial Natural Science Foundation (grant number: 2308085Y11); Scientific Research Foundation for High-level Talents of 10.13039/501100006247Anhui University of Science and Technology (grant number: 2023yjrc02).

## CRediT authorship contribution statement

**Shihao Li:** Writing – review & editing, Writing – original draft, Visualization, Methodology, Data curation. **Yinchuan Wang:** Visualization, Data curation. **Jiacheng Yin:** Formal analysis. **Kaihang Li:** Formal analysis. **Linlin Liu:** Validation, Conceptualization. **Jian Gao:** Writing – review & editing, Validation, Supervision, Funding acquisition, Conceptualization.

## Declaration of competing interest

The authors declare that they have no known competing financial interests or personal relationships that could have appeared to influence the work reported in this paper.
