# Supplementary material for: Design, synthesis, and activity evaluation of 2-iminobenzimidazoles as c-Myc inhibitors for treating multiple myeloma
Source: Heliyon. 2024 Mar 26;10(7):e28411. doi: 10.1016/j.heliyon.2024.e28411 (PMC10999938; doi:10.1016/j.heliyon.2024.e28411)
Supplement: Multimedia component 1 [file mmc1.docx]

**Supplementary Material**

**Design, synthesis, and activity evaluation of** **2-iminobenzimidazoles as c-Myc inhibitors for treating multiple myeloma**

**Shihao Li^1,†^, Yinchuan Wang^1,†^, Jiacheng Yin^1^, Kaihang Li^1^, Linlin Liu^2,^*****, and Jian Gao^1,3,^***

*^1^Jiangsu Key Laboratory of New Drug Research and Clinical Pharmacy, Xuzhou Medical University, Xuzhou, Jiangsu 221004, P. R. China*

*^2^College of Medical Imaging, Xuzhou Medical University, Xuzhou, Jiangsu 221004, P. R. China*

*^3^School of Medicine, Anhui University of Science and Technology, Huainan, China*

^†^These authors contributed equally


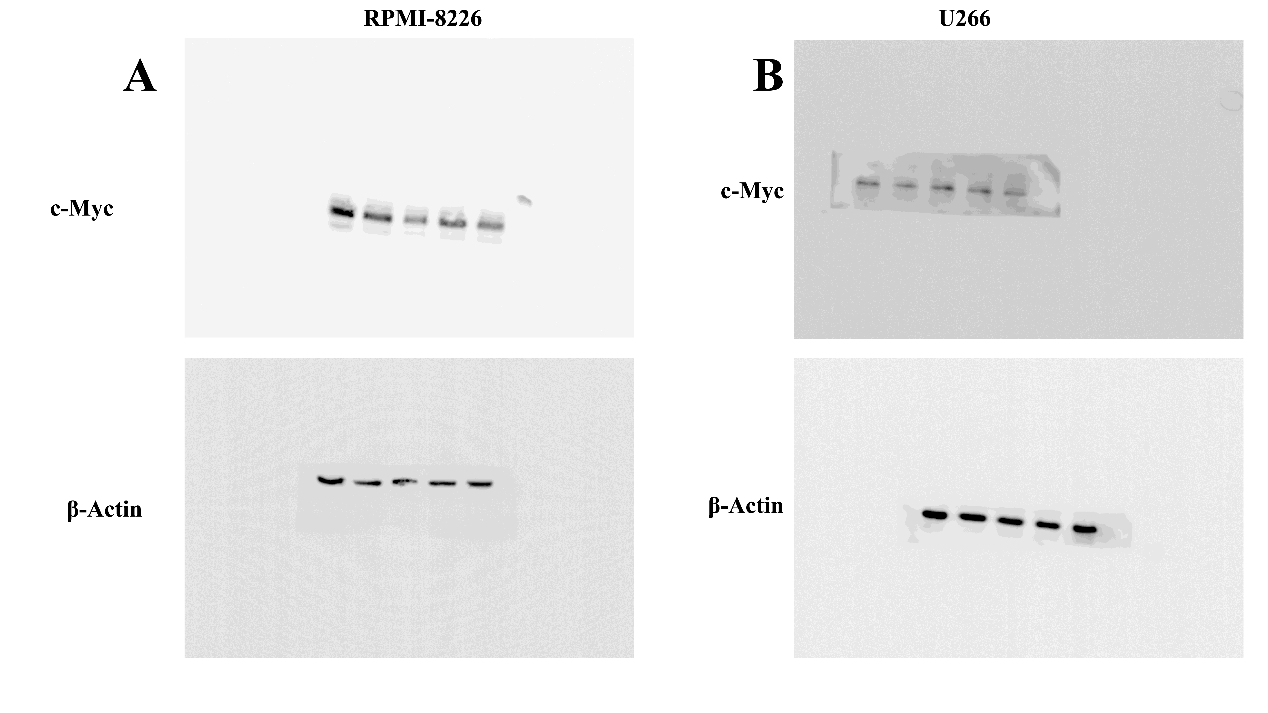


**Fig.** **S1** Western blot analysis of RPMI-8226 and U266 cells treated with 1 μM 8g, 5b, and 5d. The positive control used was 10074-G5 (The uncropped version).

**Table S1.** Inhibitory effect of **5b, 5d,** and **8g** on human renal epithelial cells 293T.

| **Compounds** | **R^1^** | **R^2^** | **IC_50_ (μM)^1^**  HEK 293T | |
| --- | --- | --- | --- | --- |
| **5b** | **** | **** | | 92.14±8.20 |
| **5d** | **** |  | | 95.47±9.68 |
| **8g** | **** |  | | 90.76±8.54 |

^1^ IC_50_, the mean ± SD value of triplicate measurement.

Table of contents

1. NMR spectra of final compounds S3-S36
2. ESI-HRMS (TOF) of final compounds S37-S48

**1. NMR spectra of final compounds**


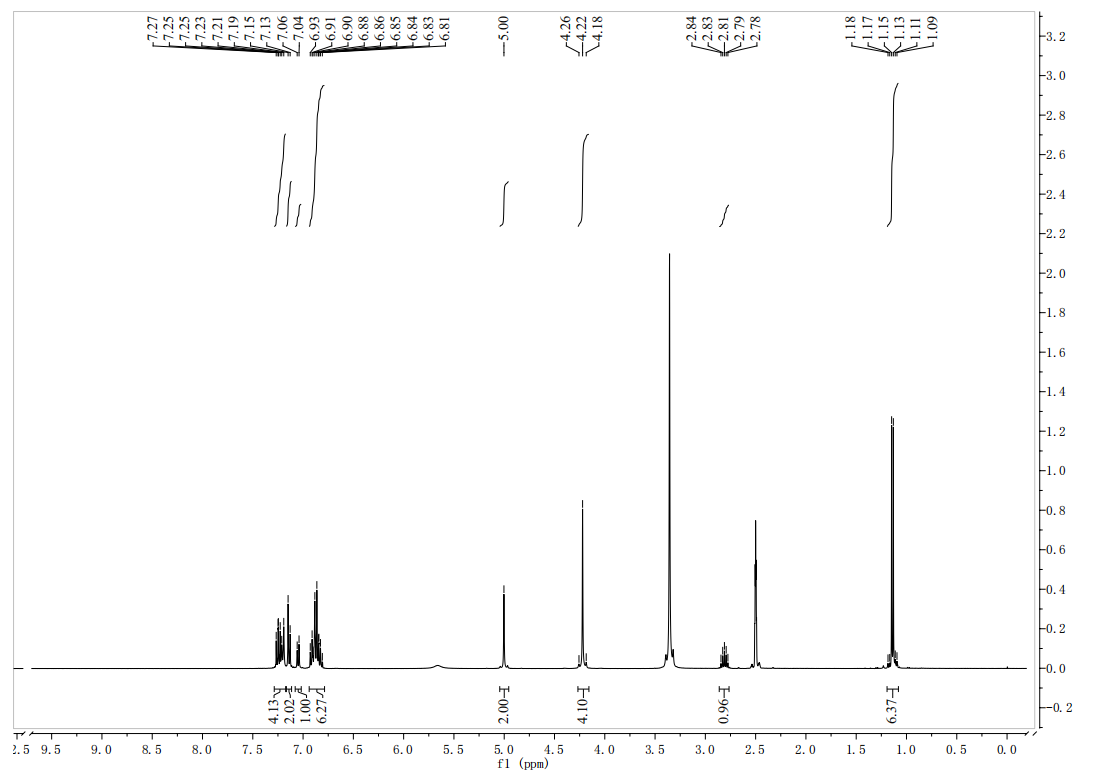

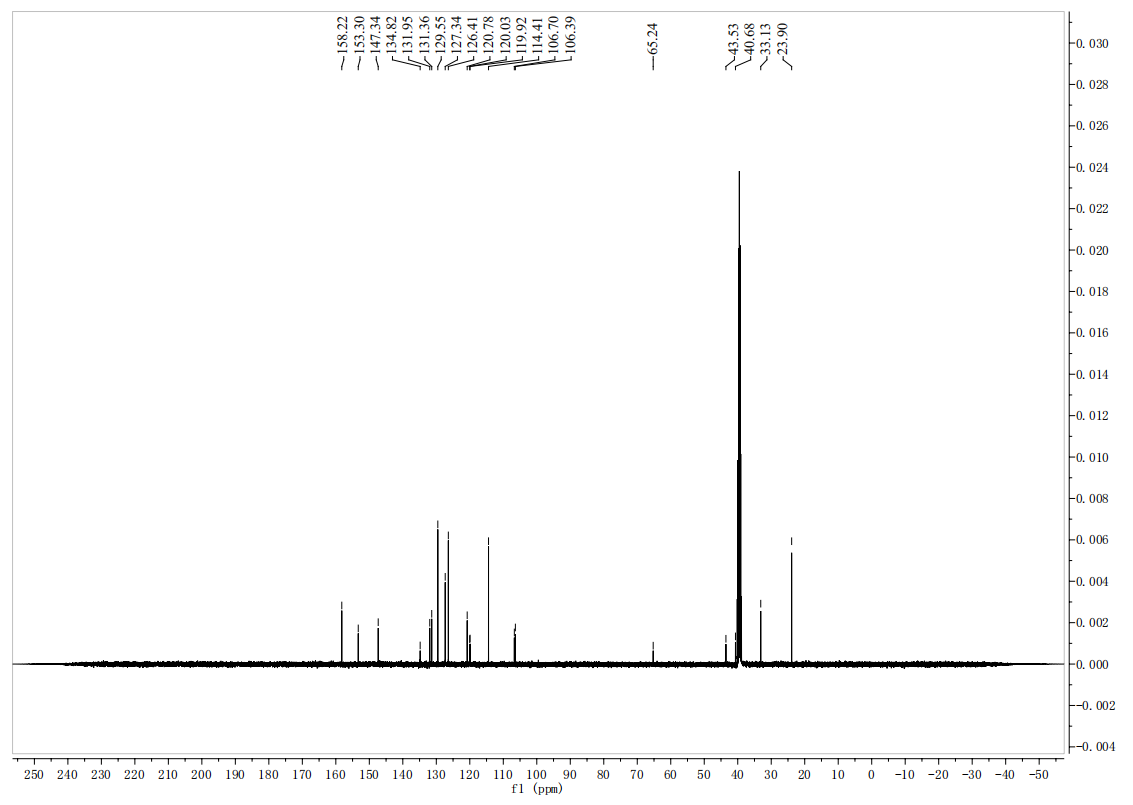


NMR spectra of compound **5a**
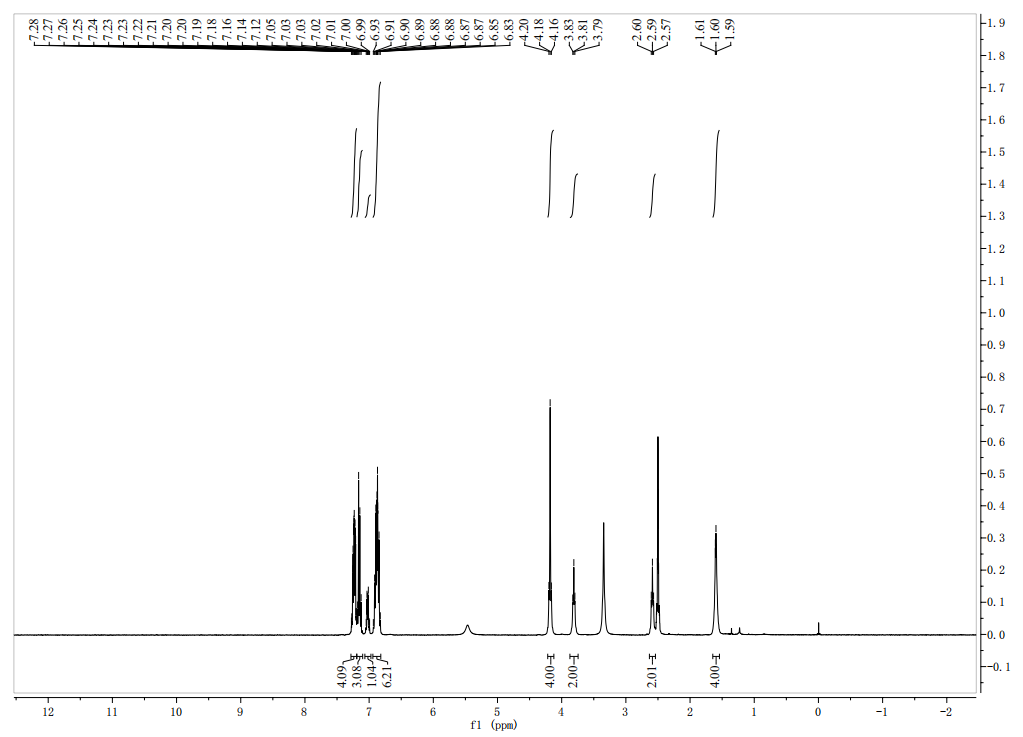

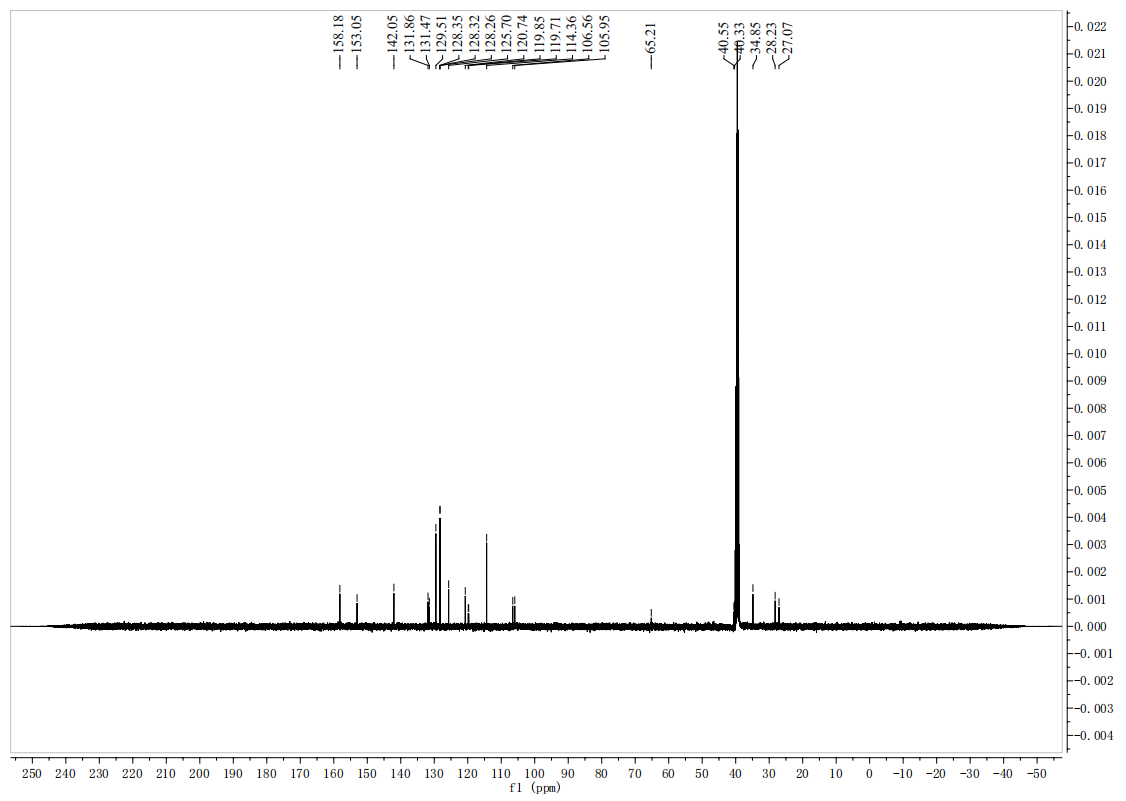


NMR spectra of compound **5b**
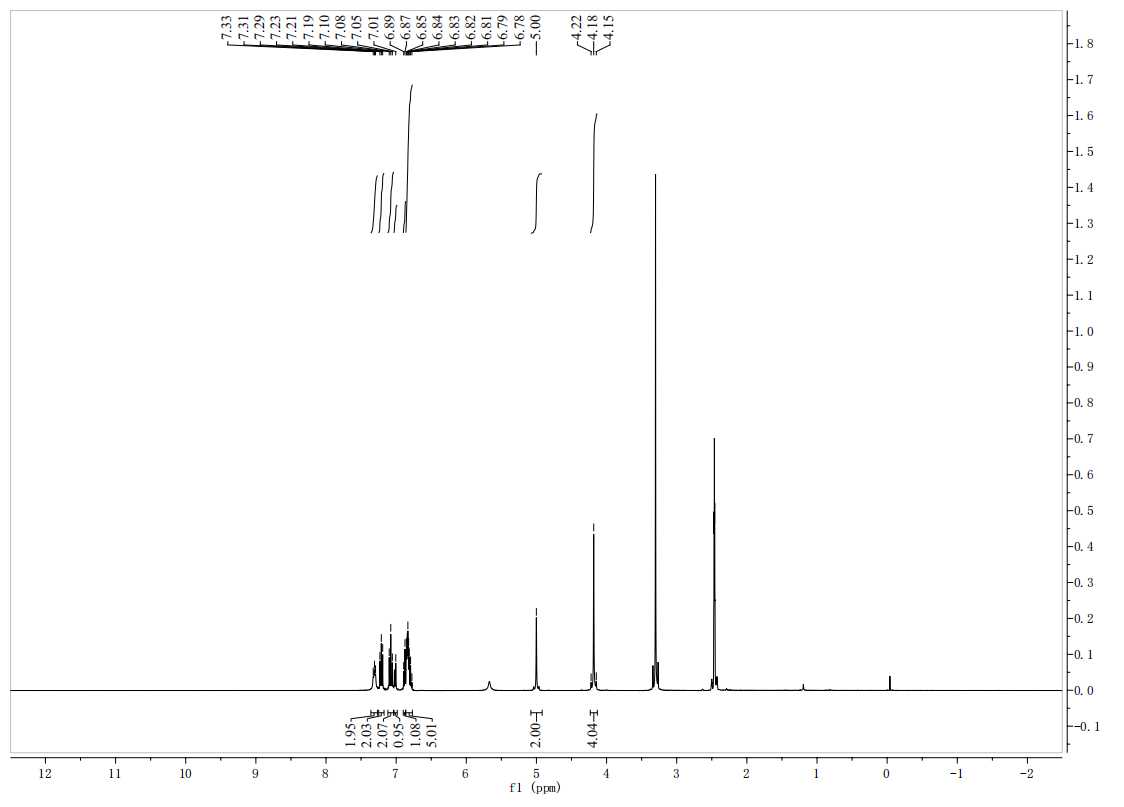

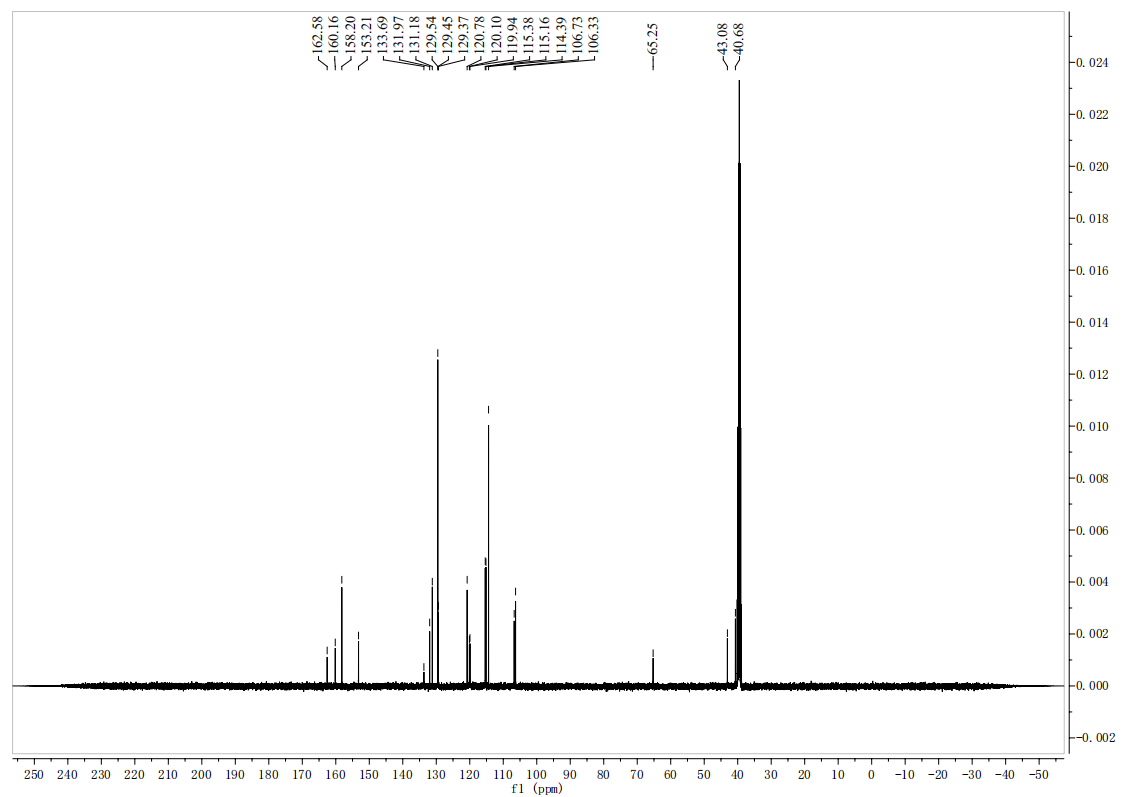


NMR spectra of compound **5c**


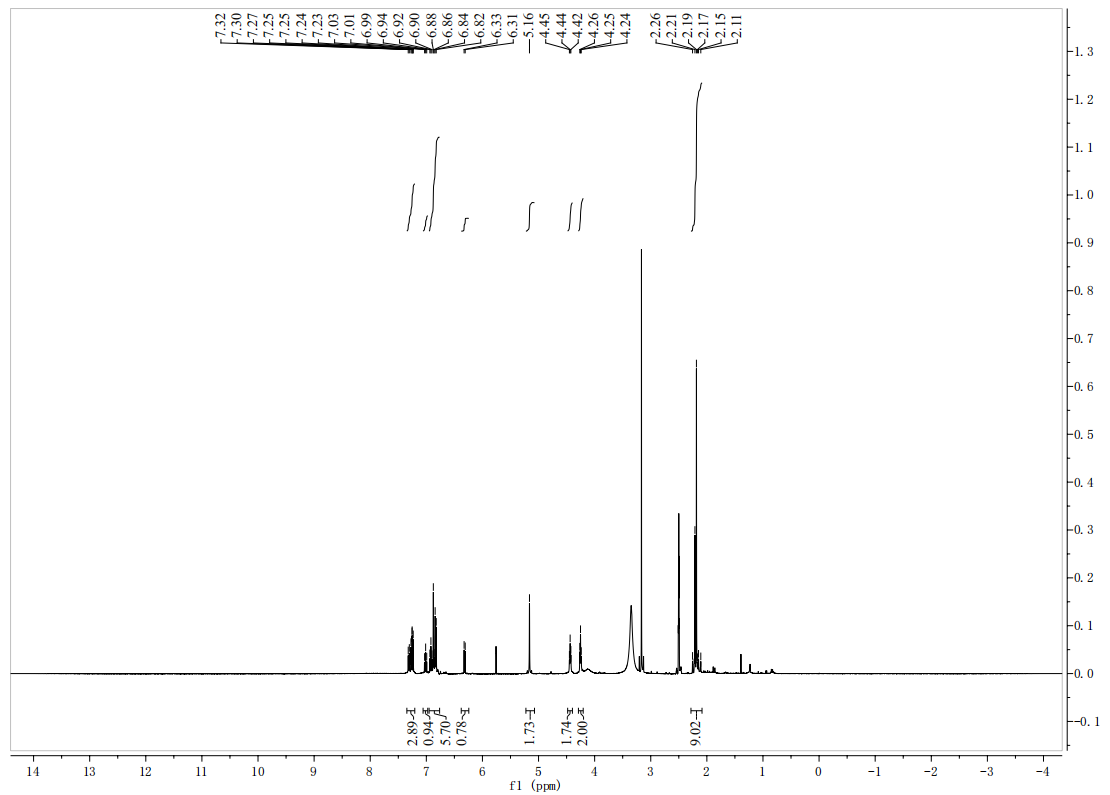

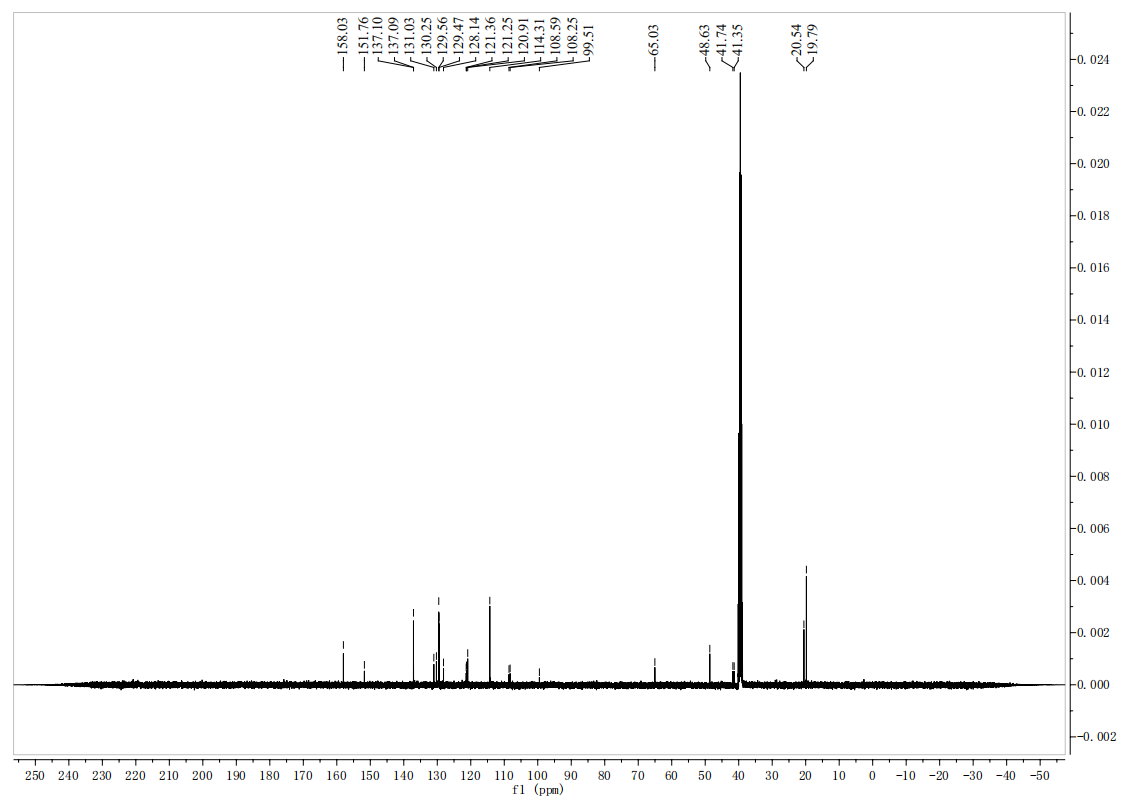


NMR spectra of compound **5d**


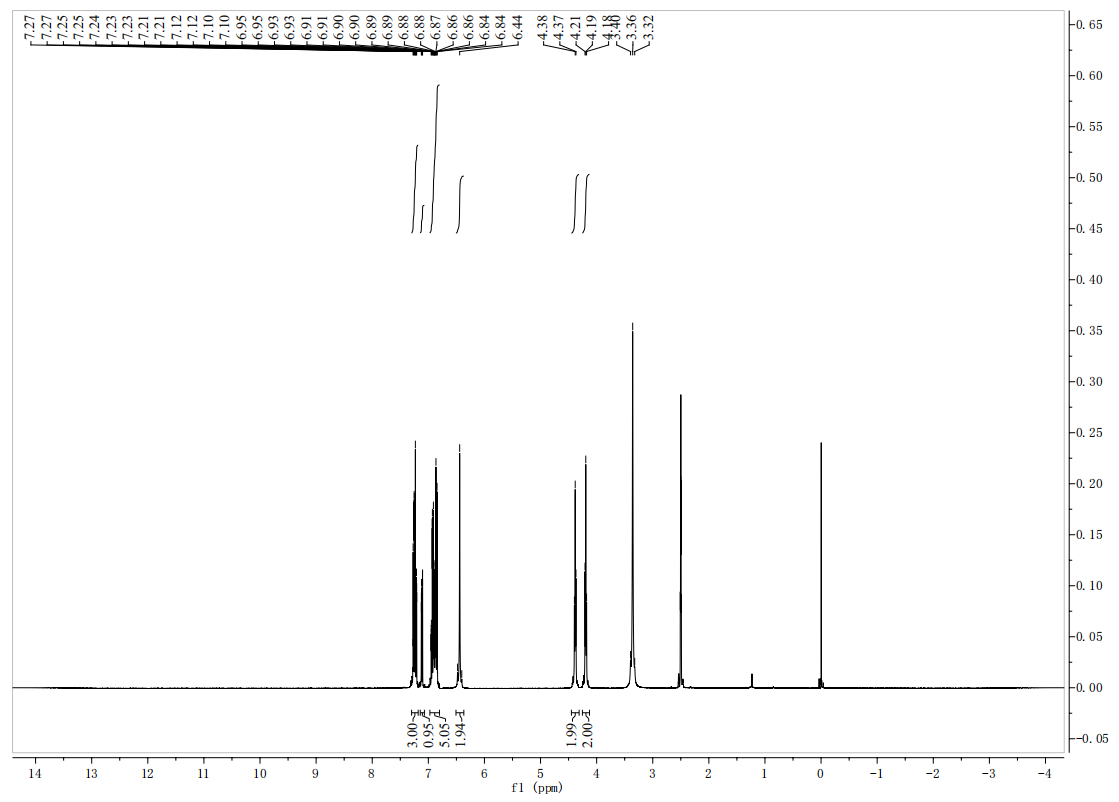

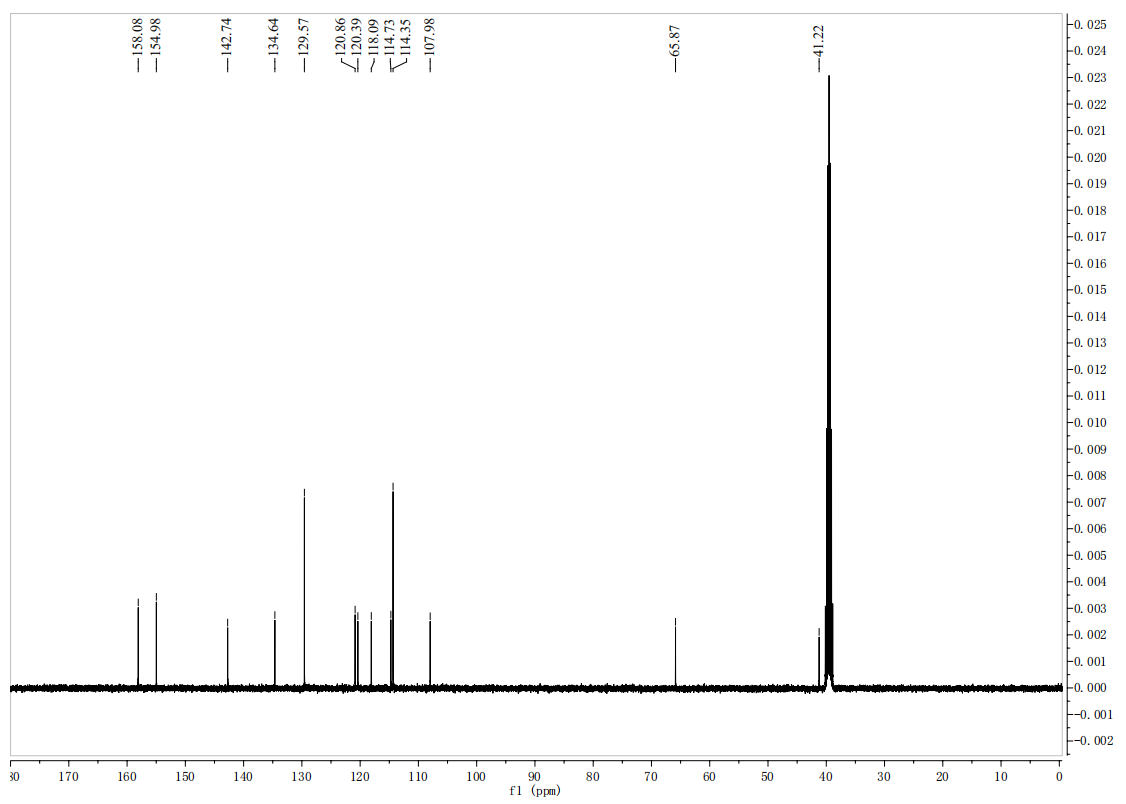


NMR spectra of compound **6a**


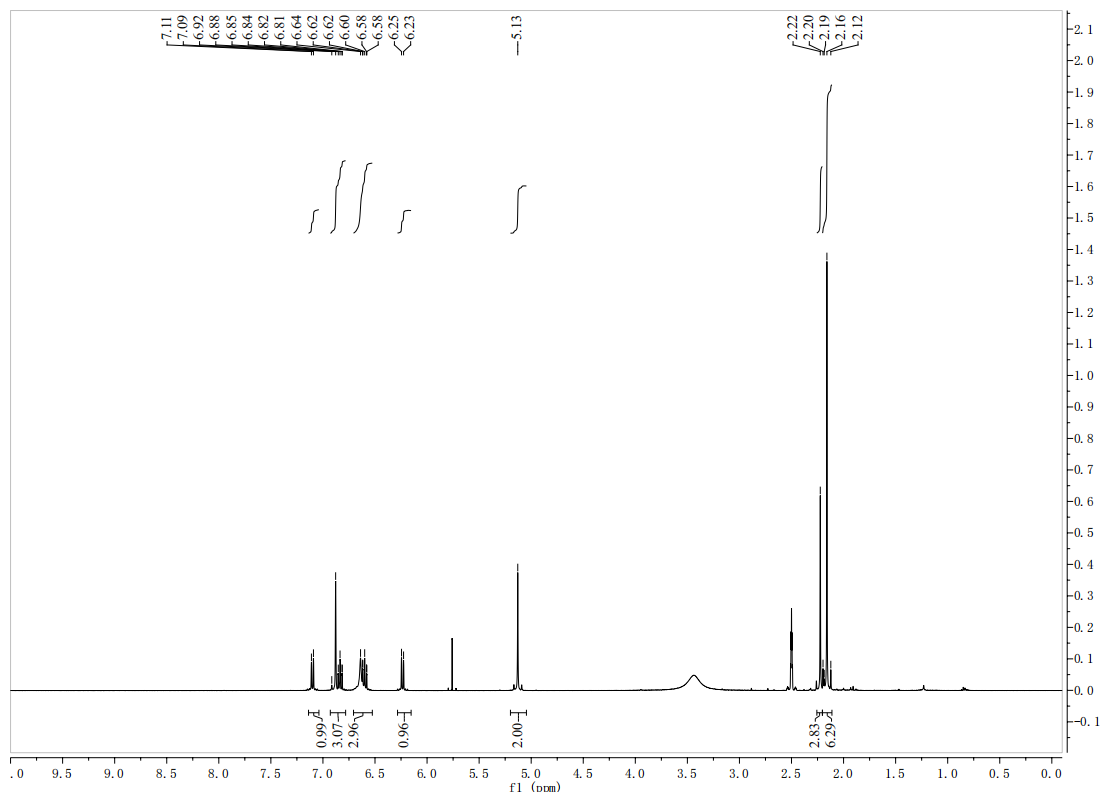

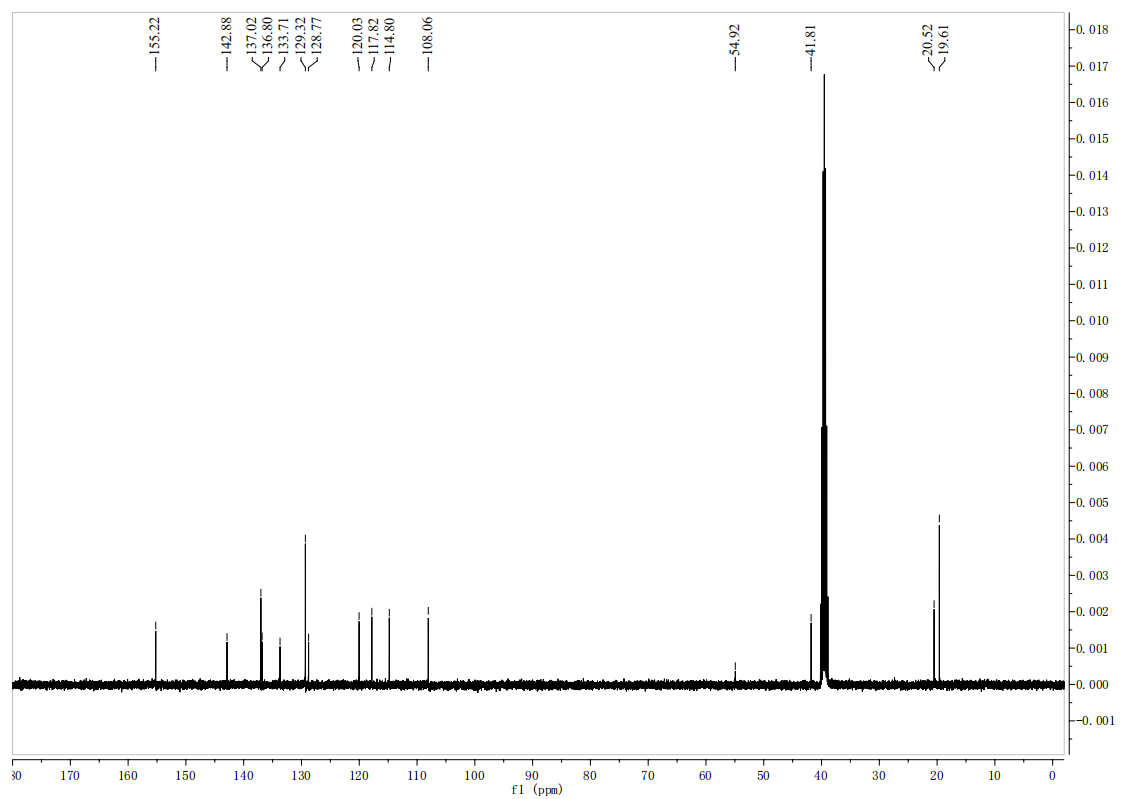


NMR spectra of compound **6b**


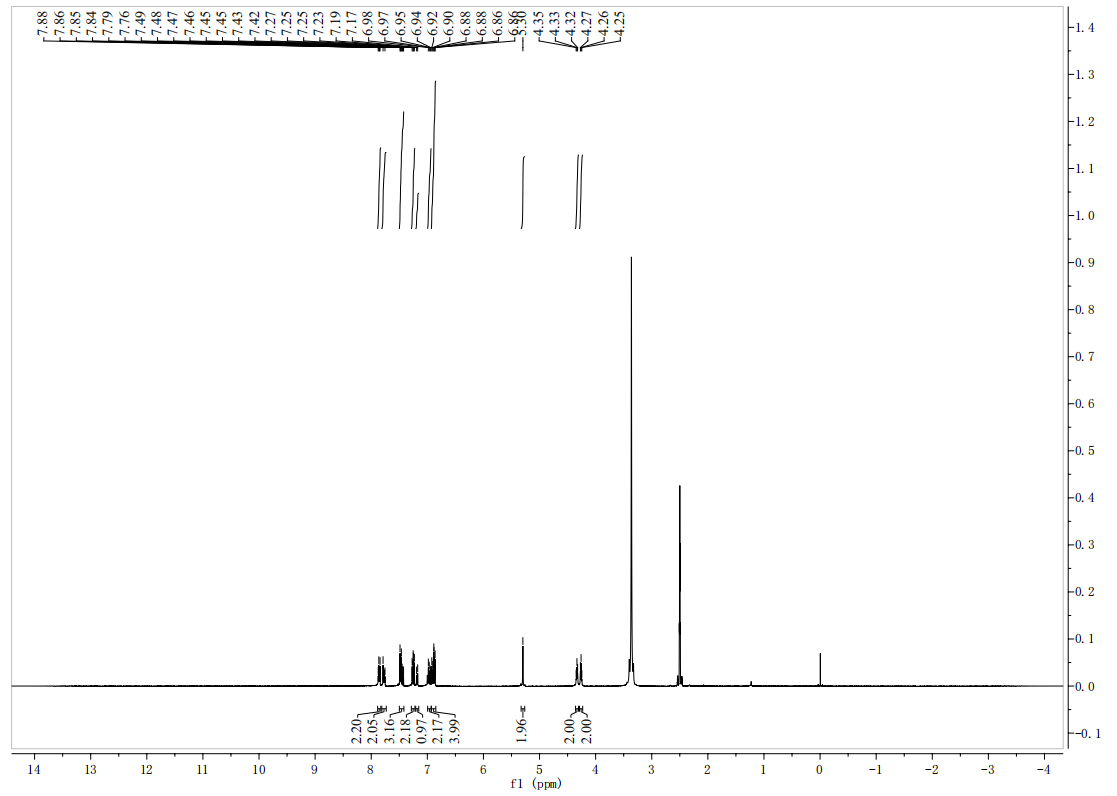


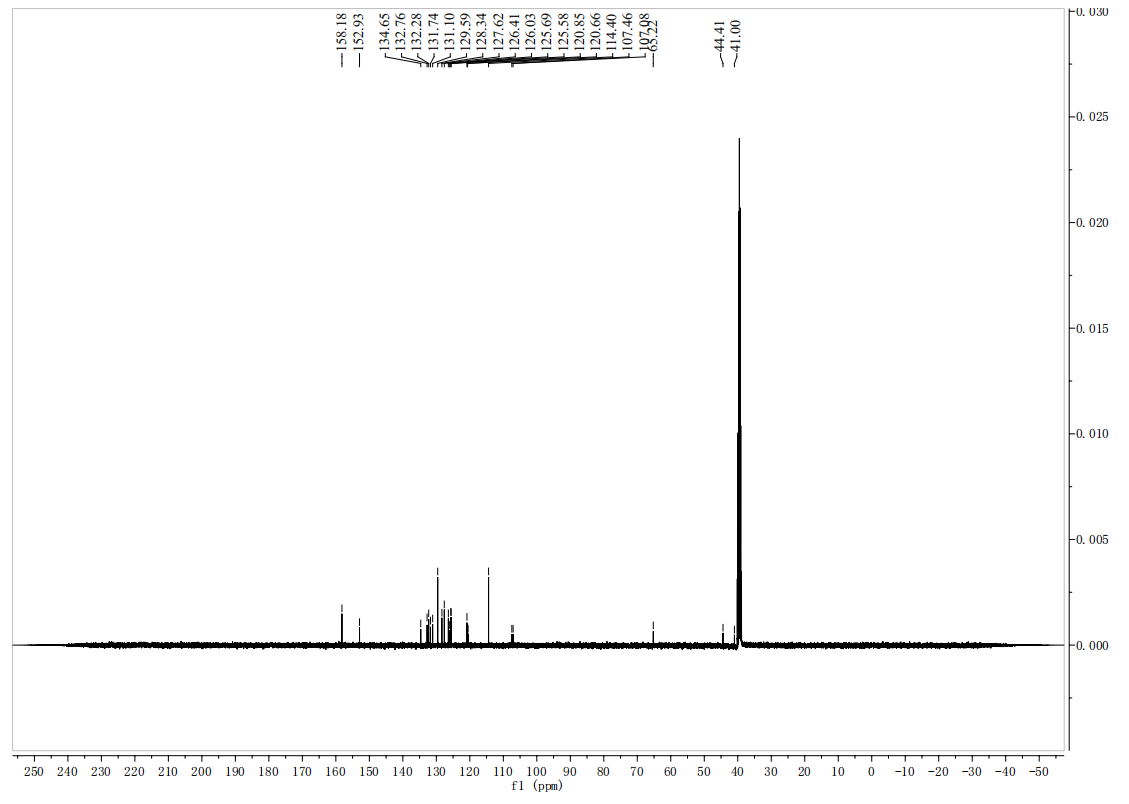


NMR spectra of compound **8a**
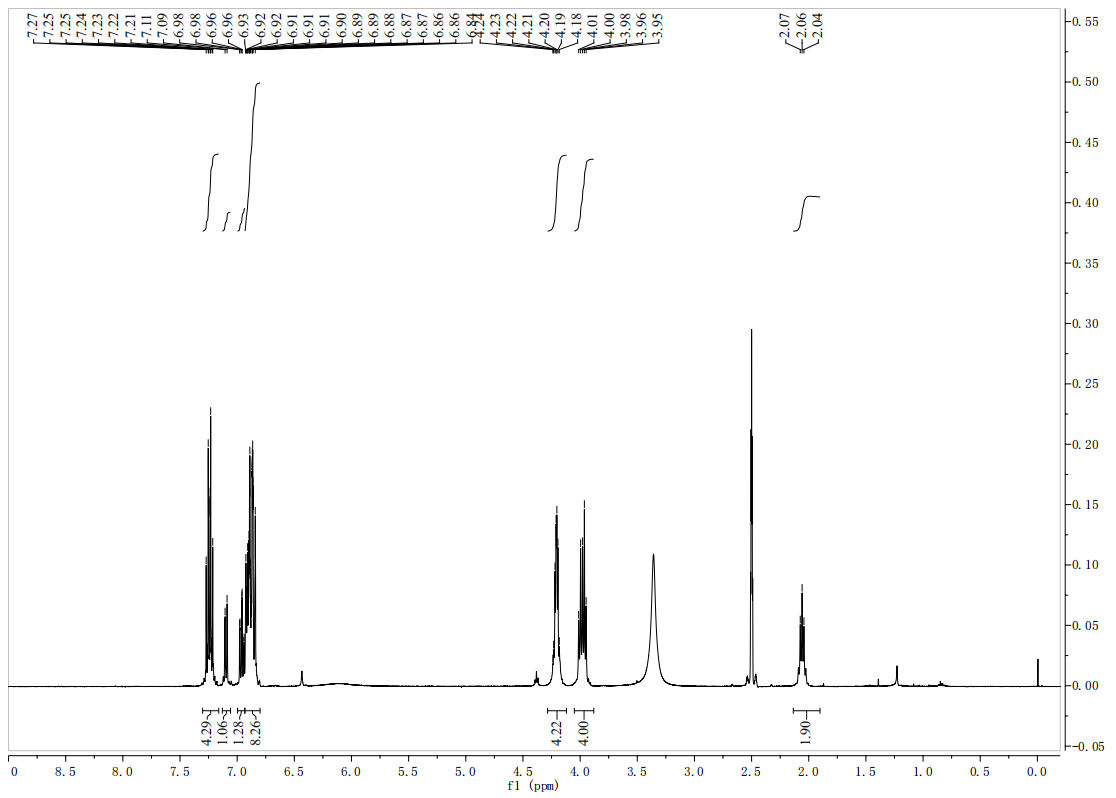

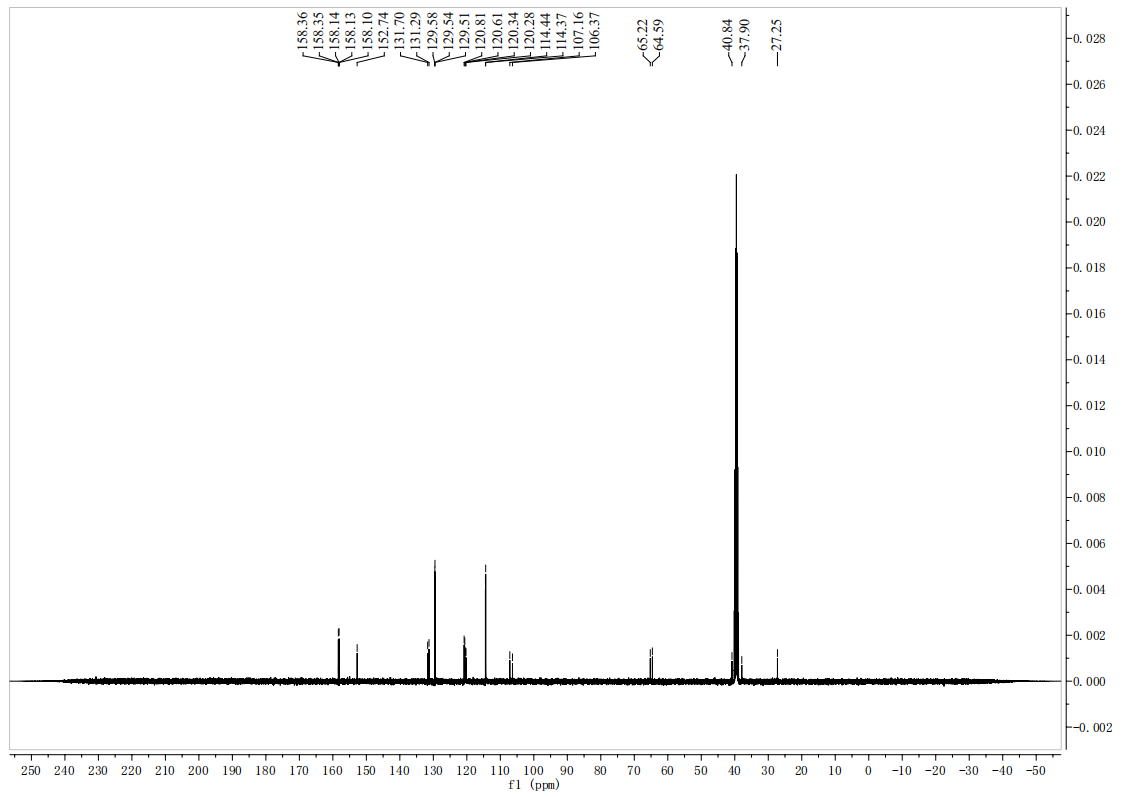


NMR spectra of compound **8b**
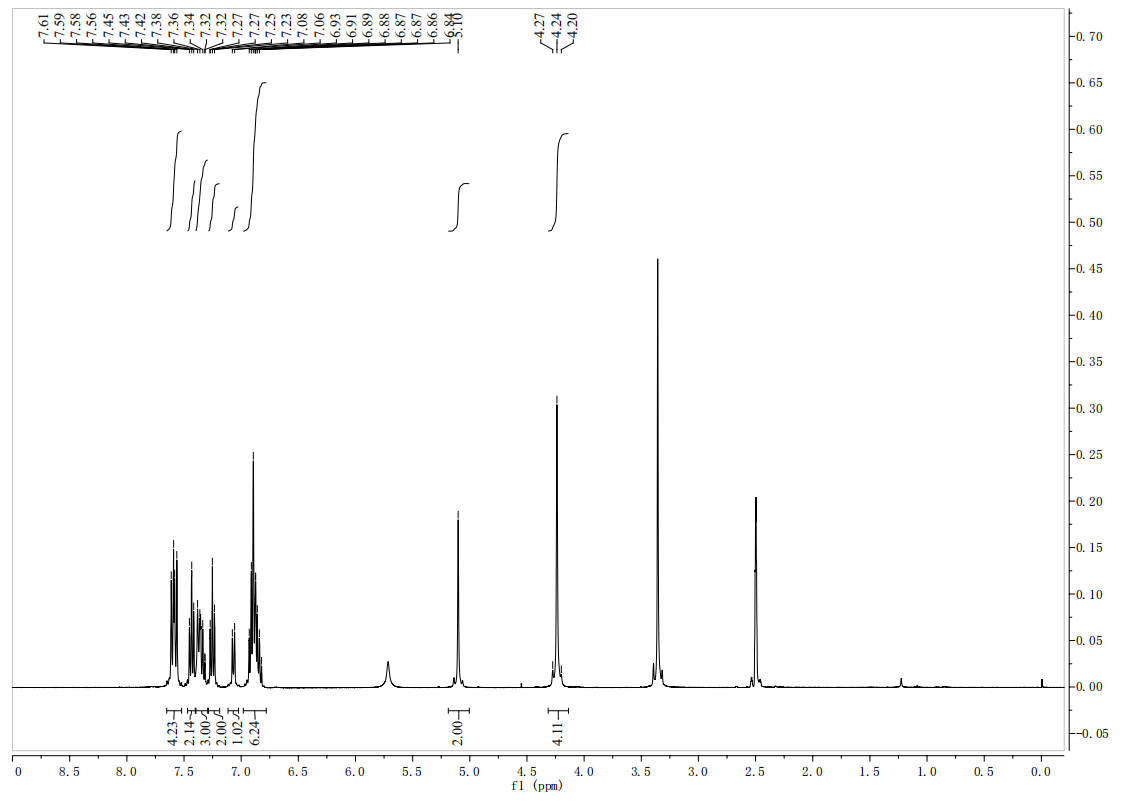

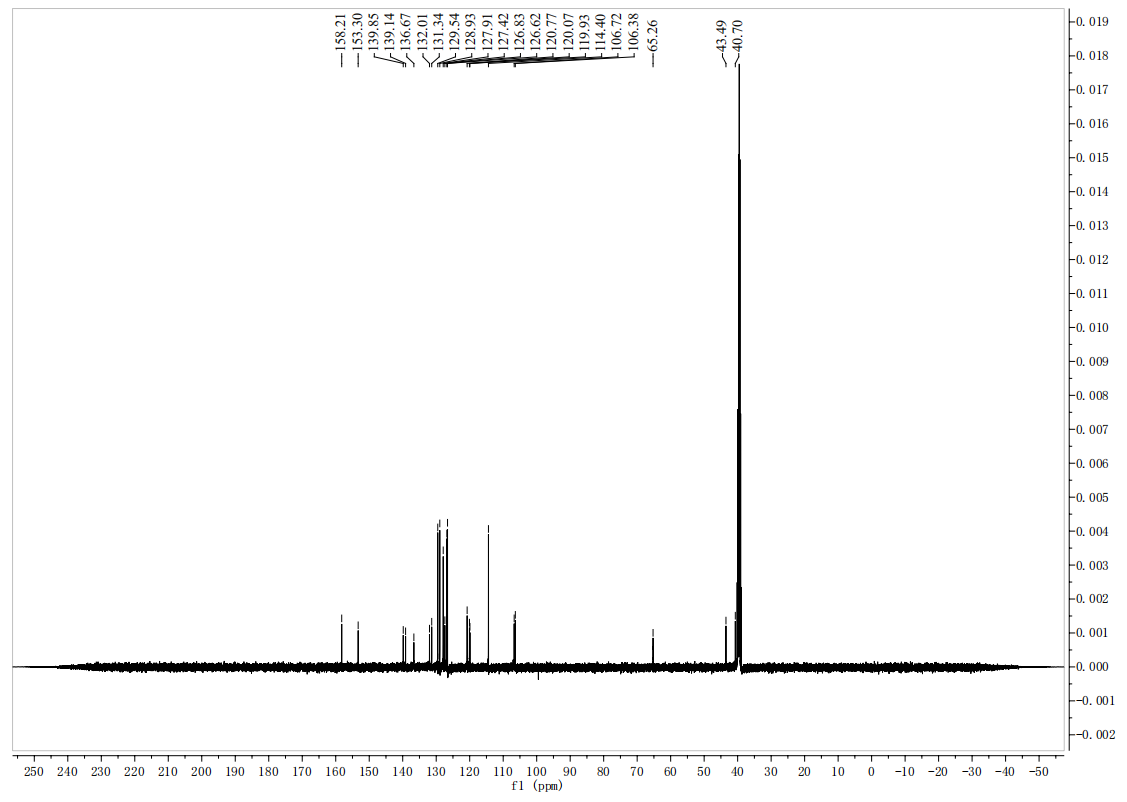


NMR spectra of compound **8c**
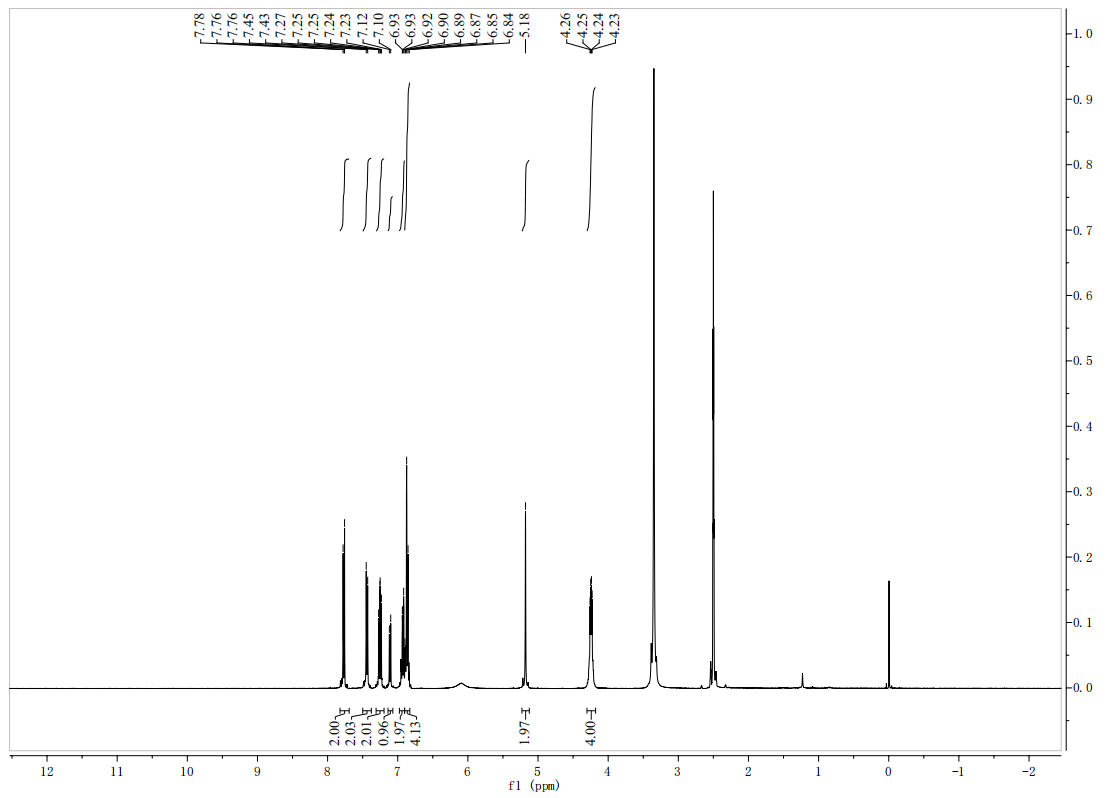

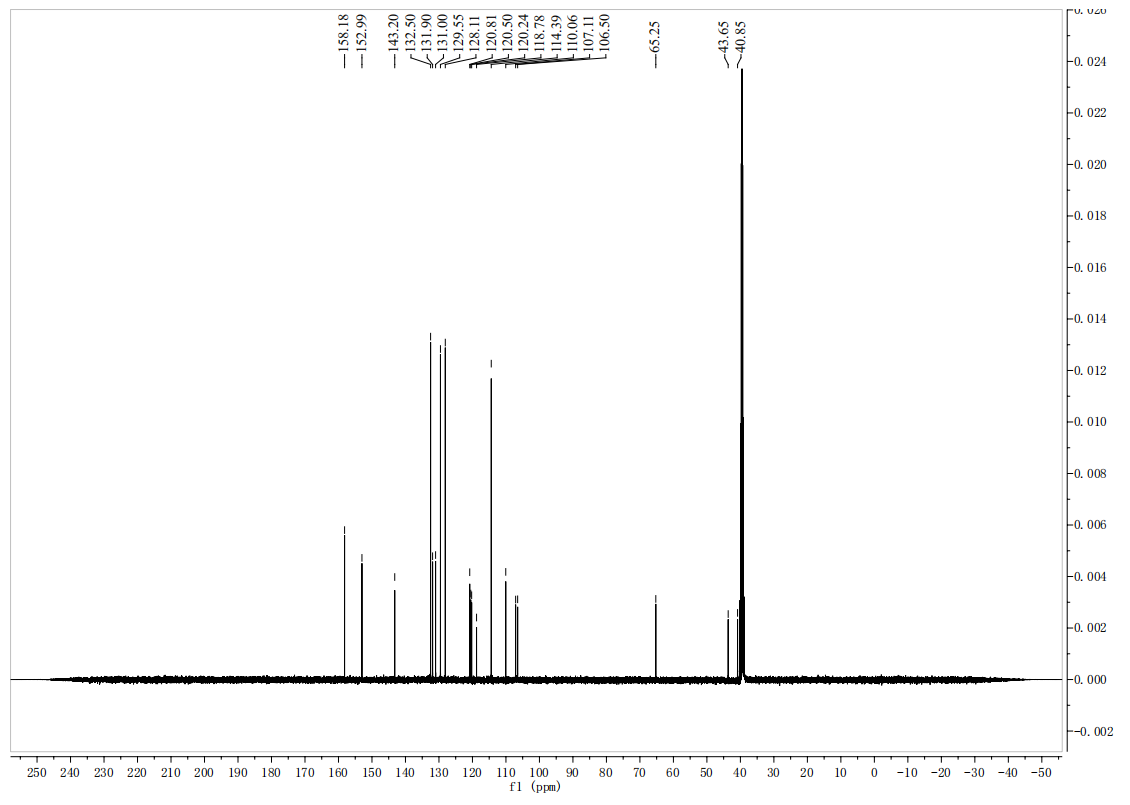


NMR spectra of compound **8d**
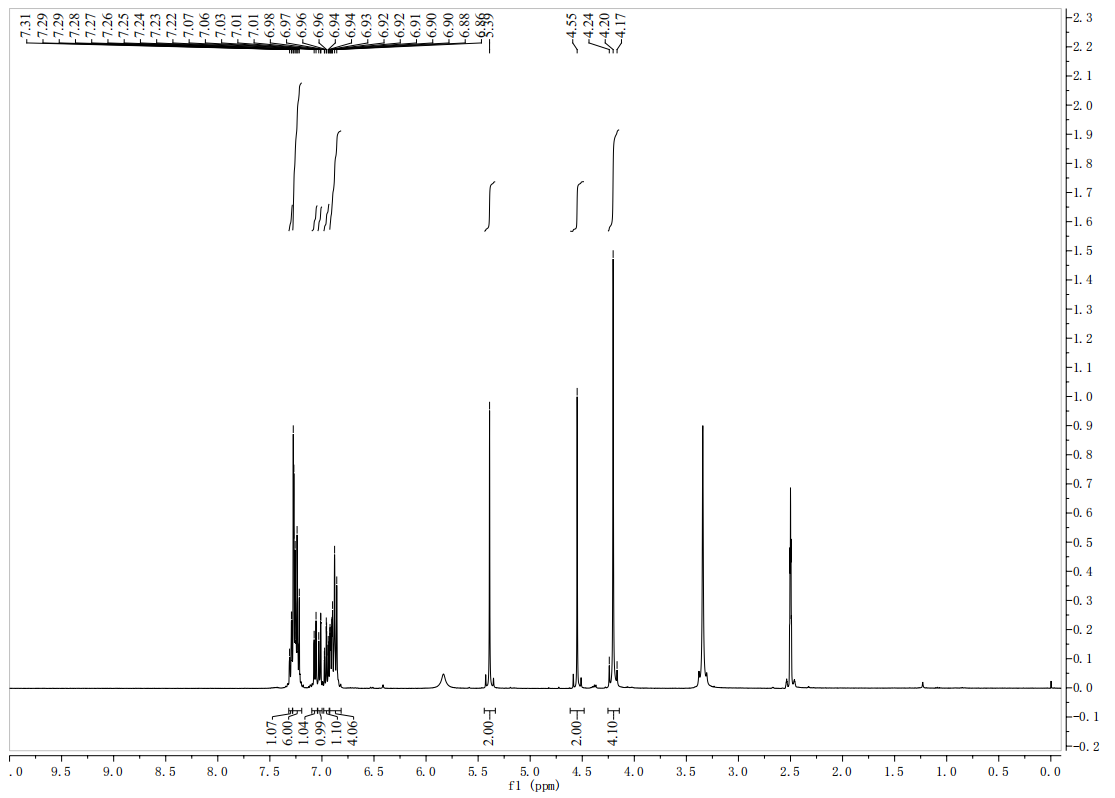

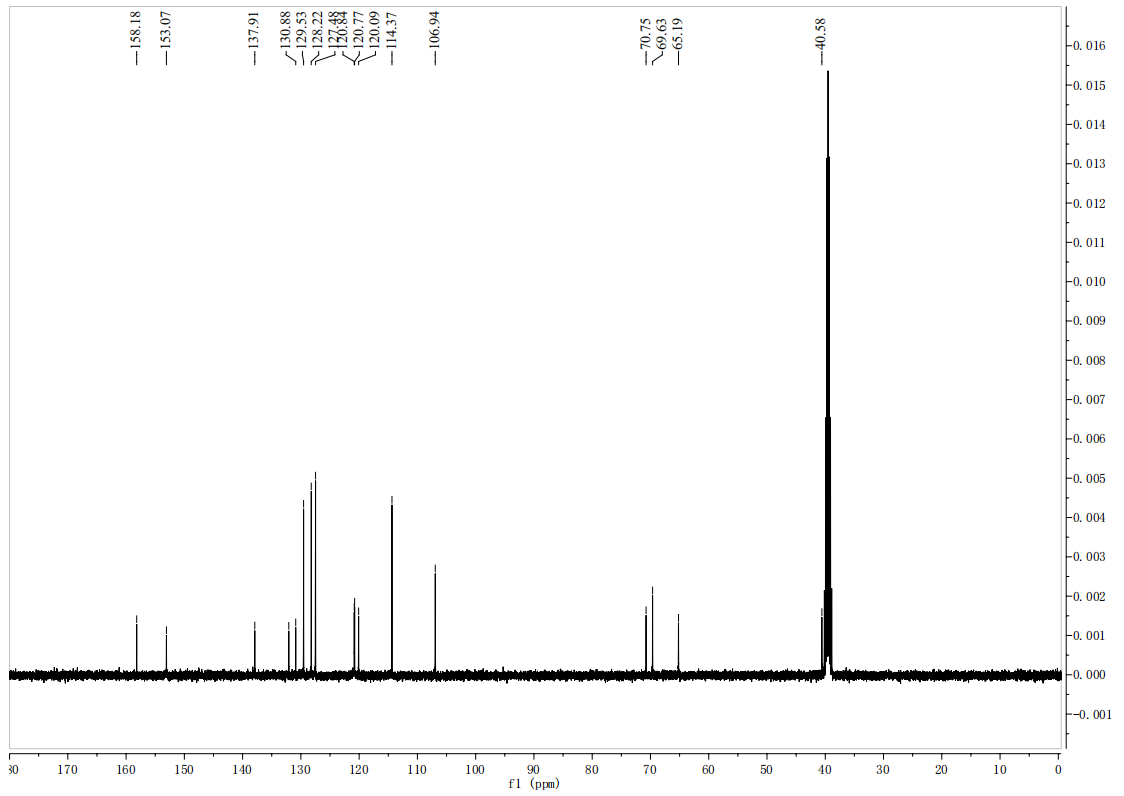


NMR spectra of compound **8e**
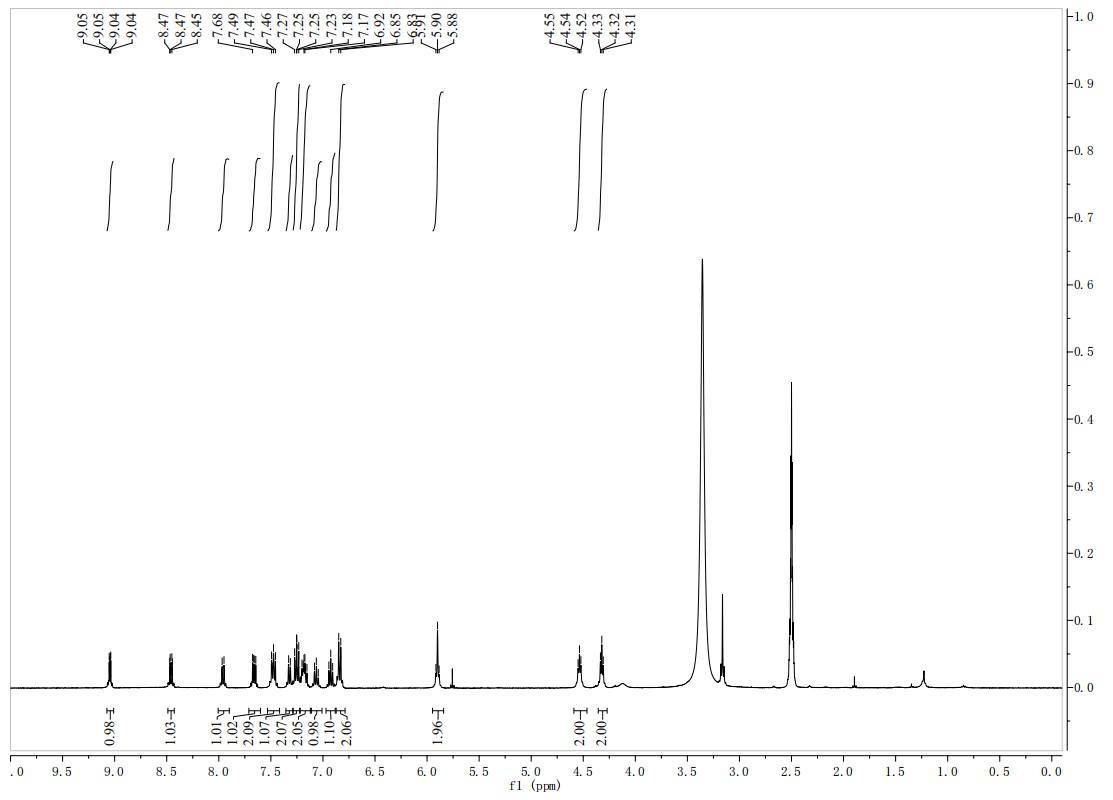

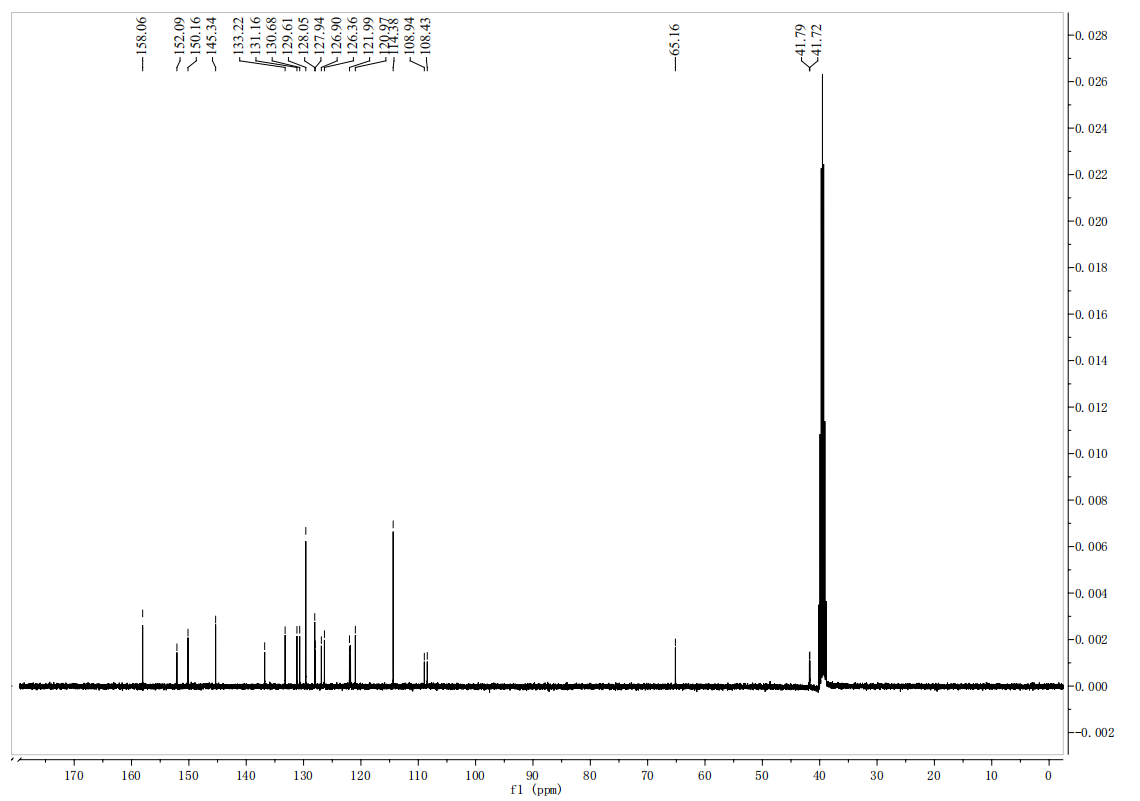


NMR spectra of compound **8f**
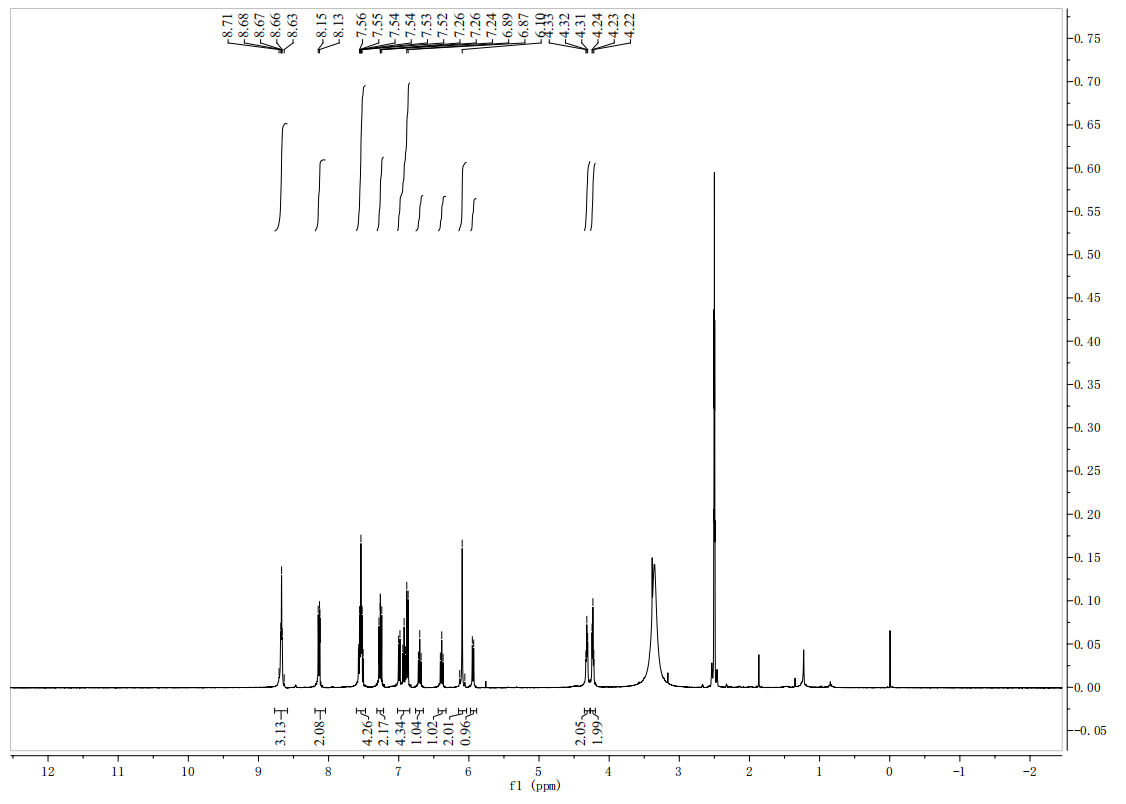

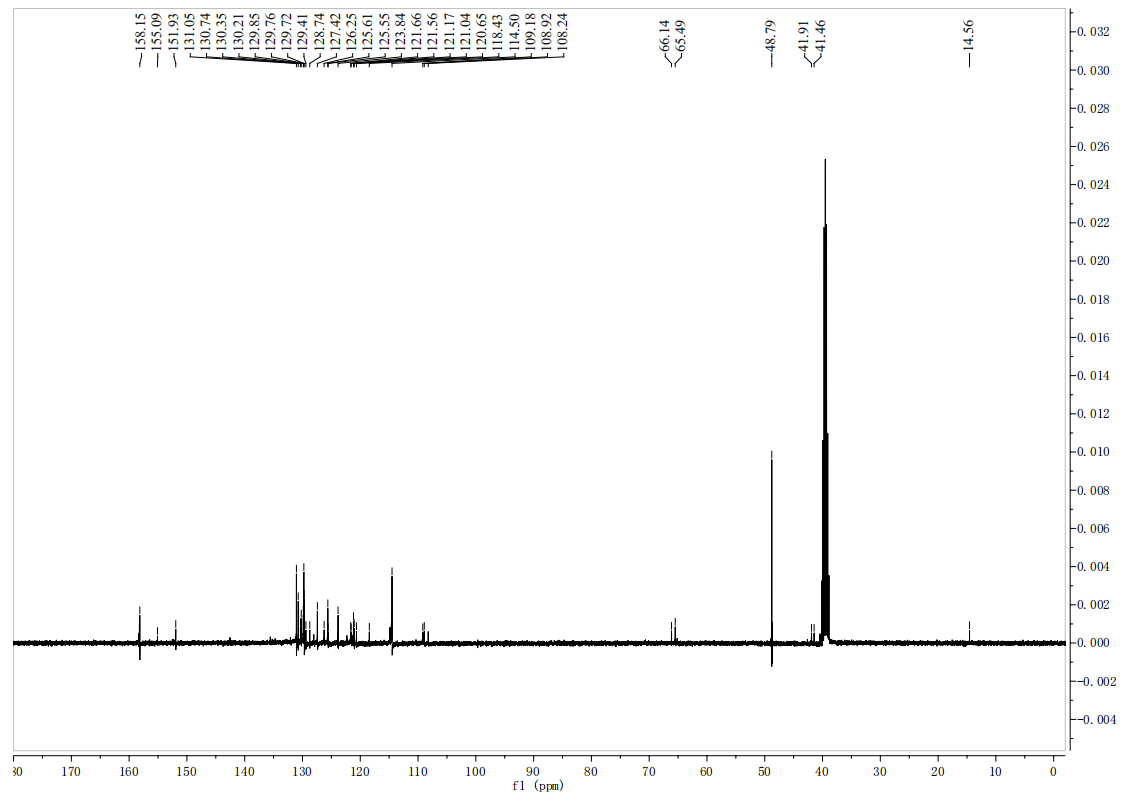


NMR spectra of compound **8g**
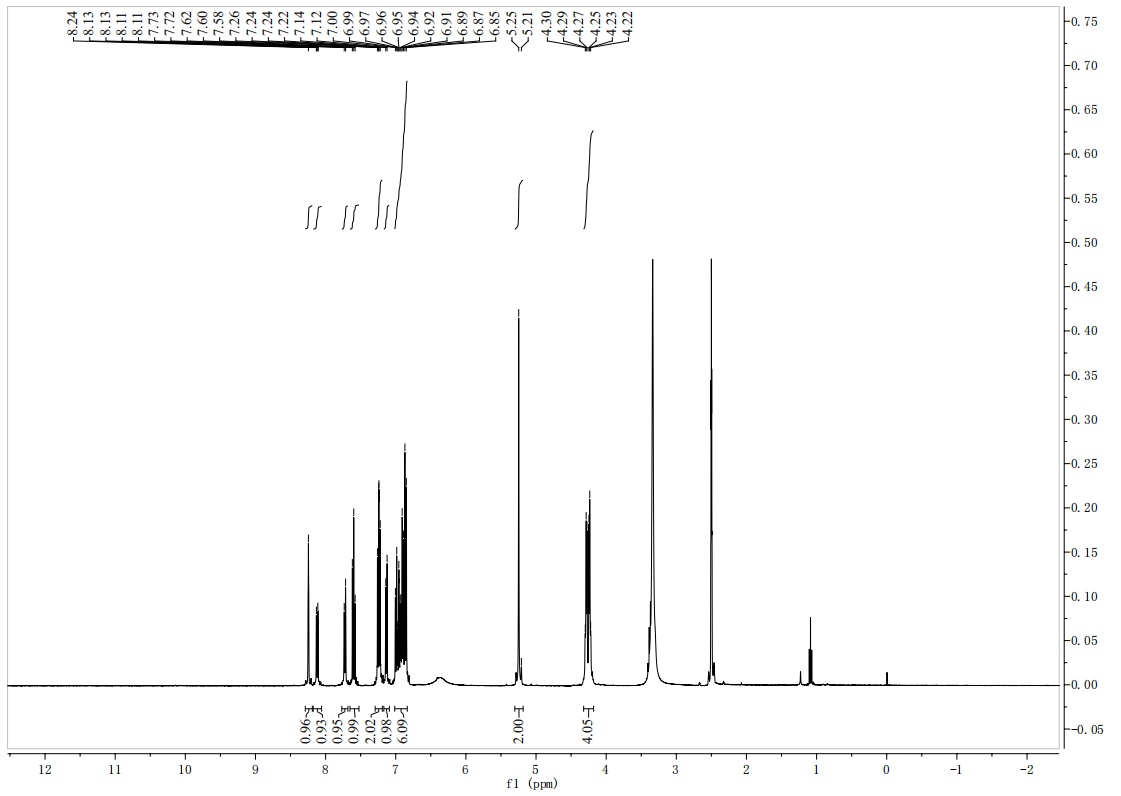

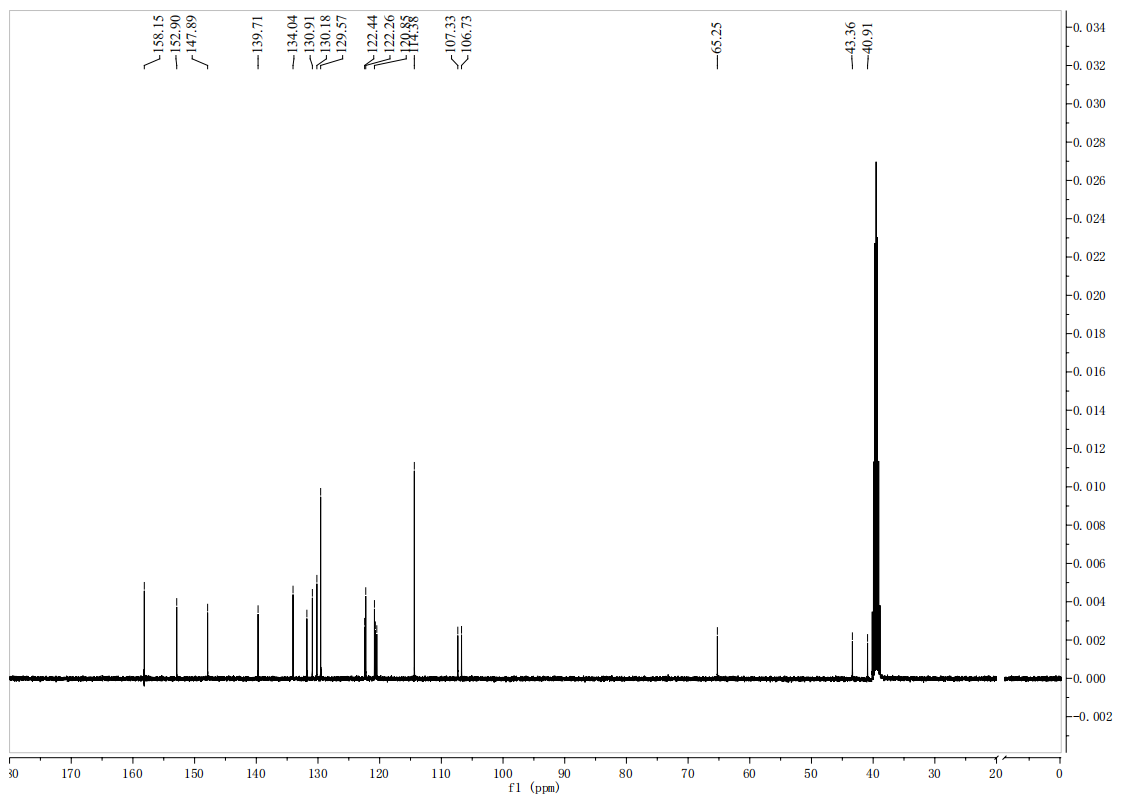


NMR spectra of compound **8h**
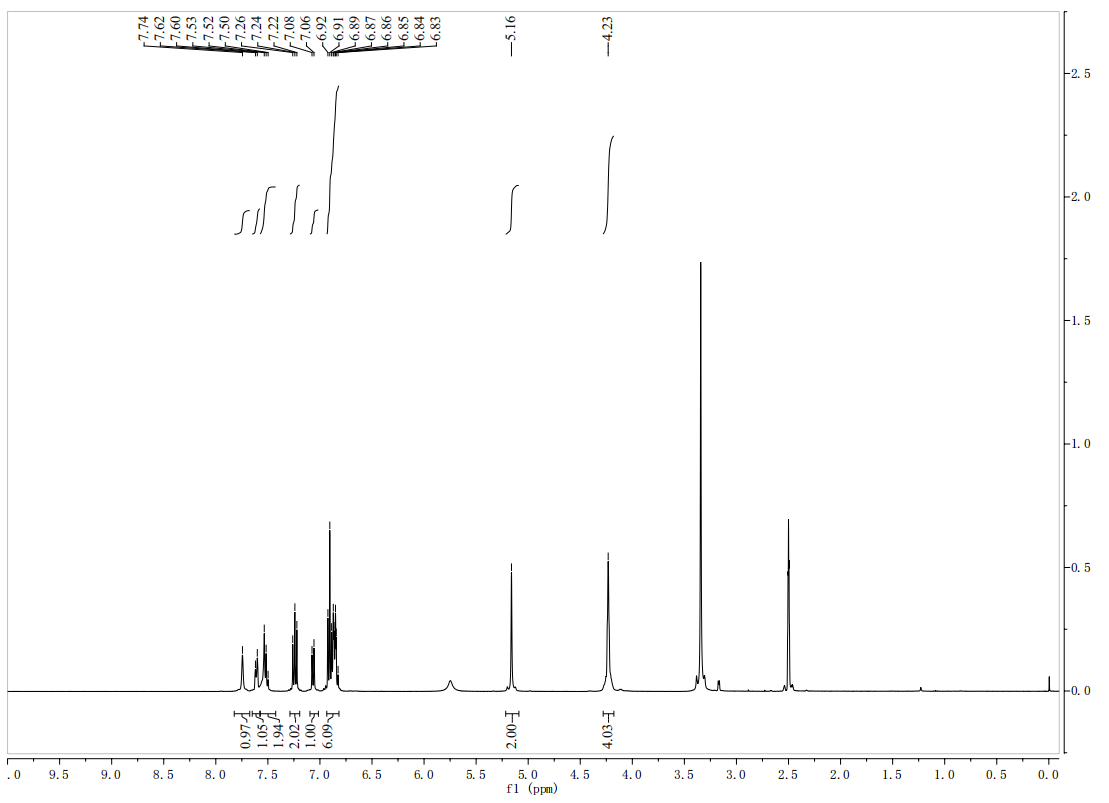

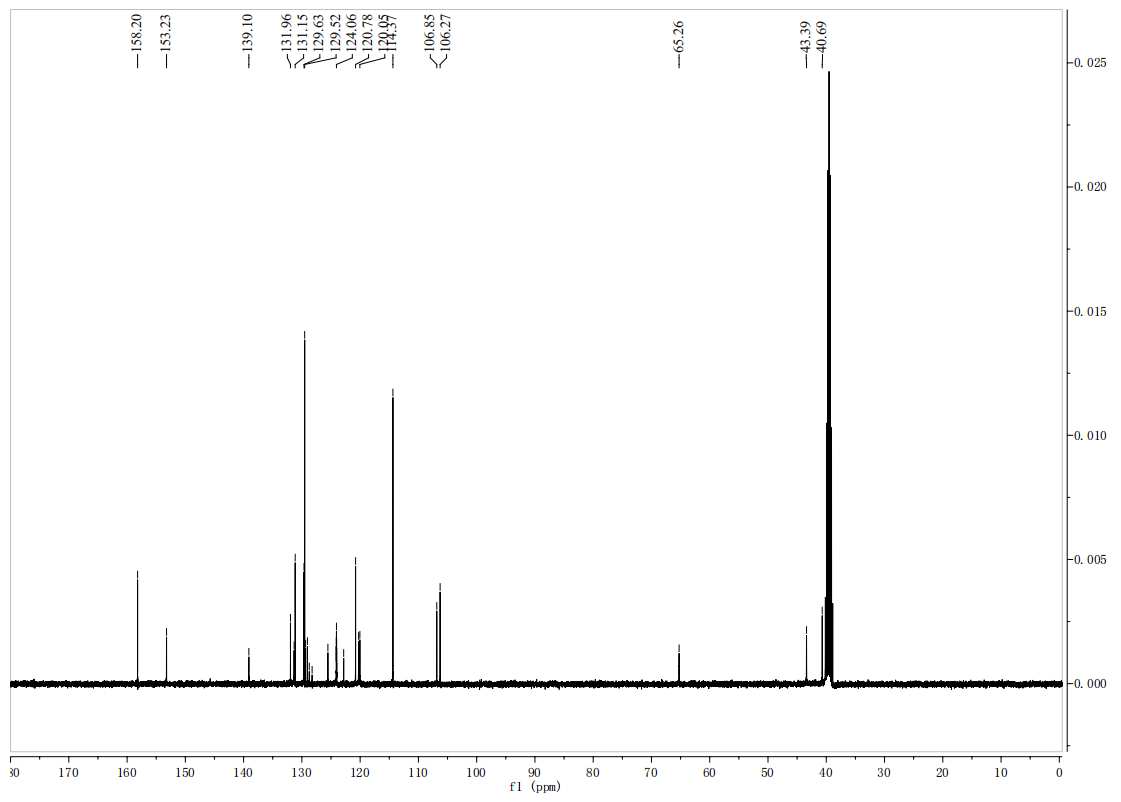


NMR spectra of compound **8i**
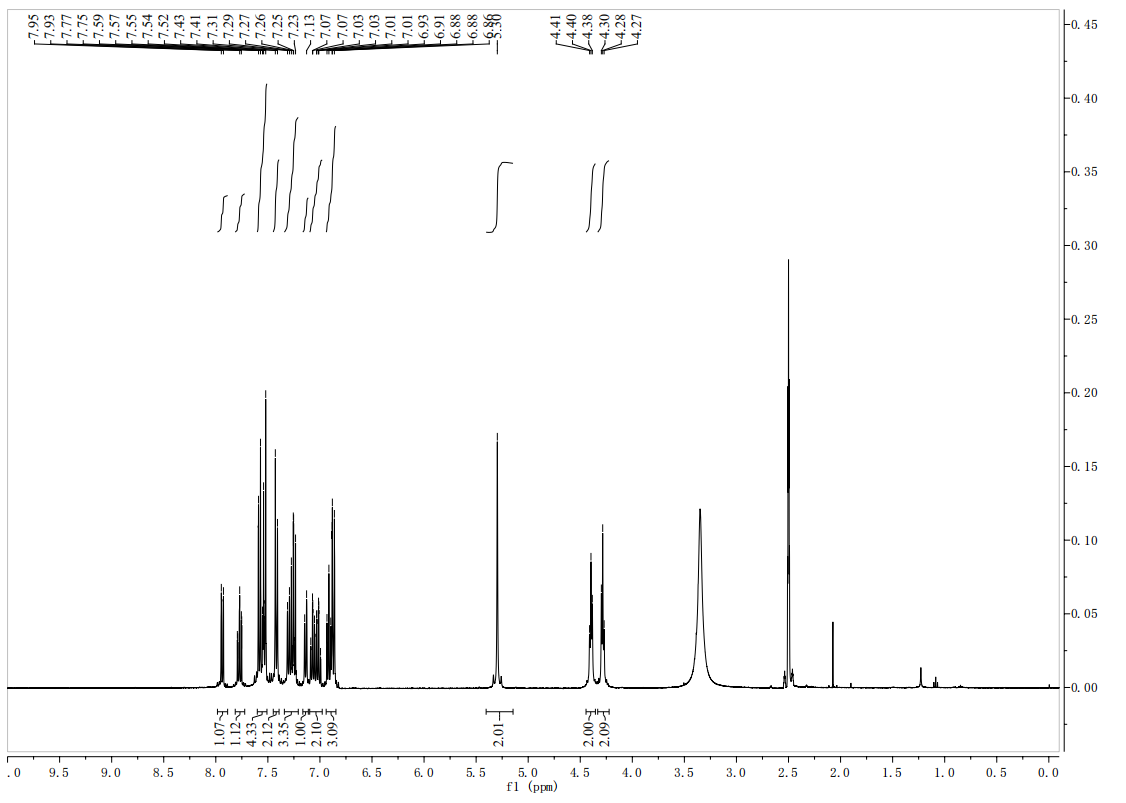

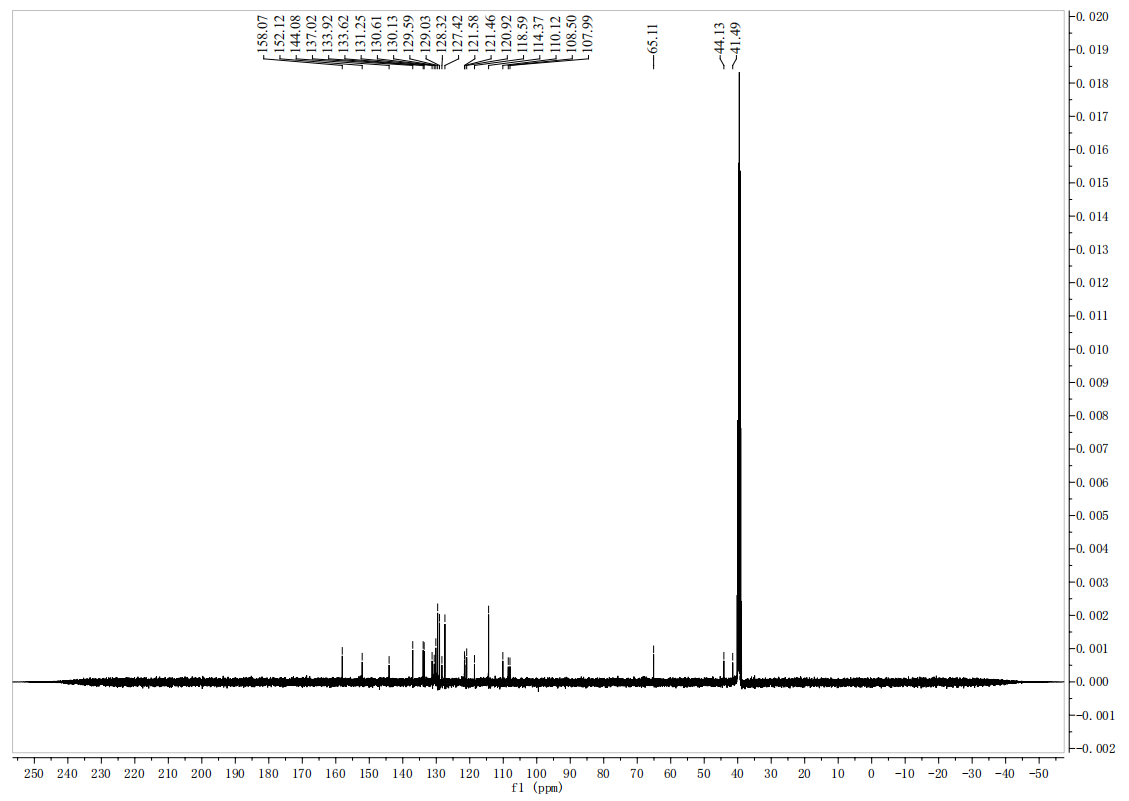


NMR spectra of compound **8j**
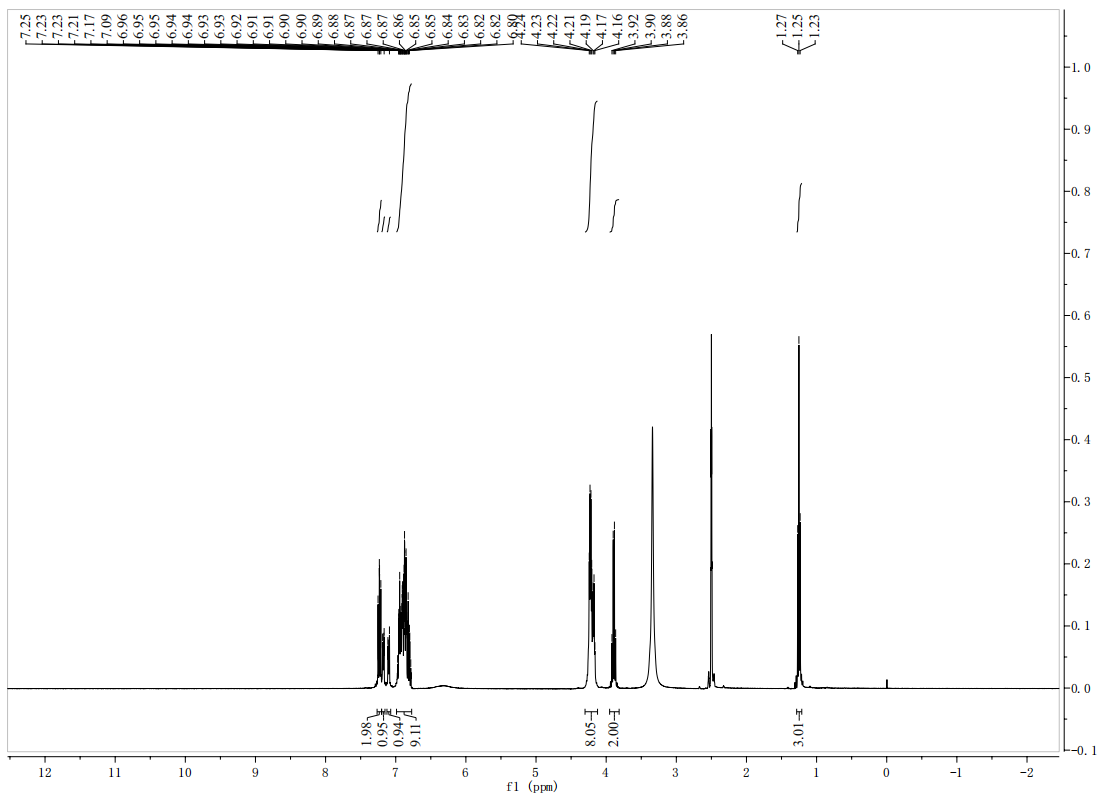

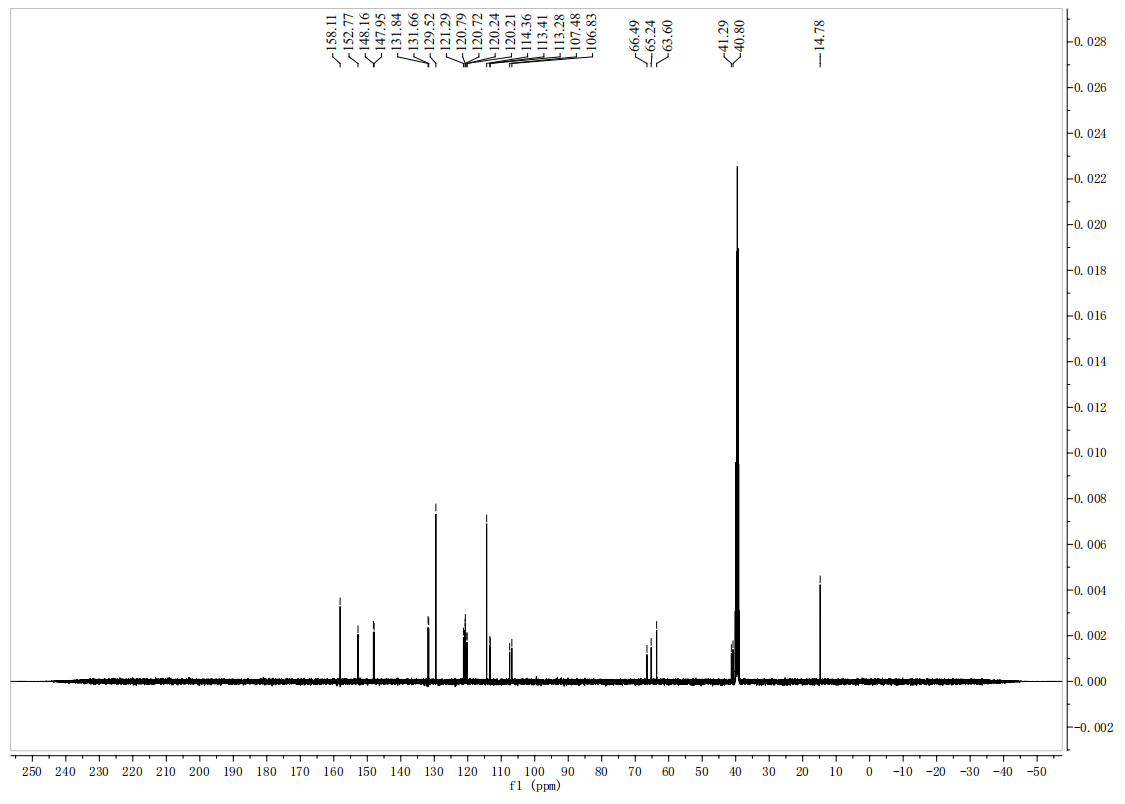


NMR spectra of compound **8k**
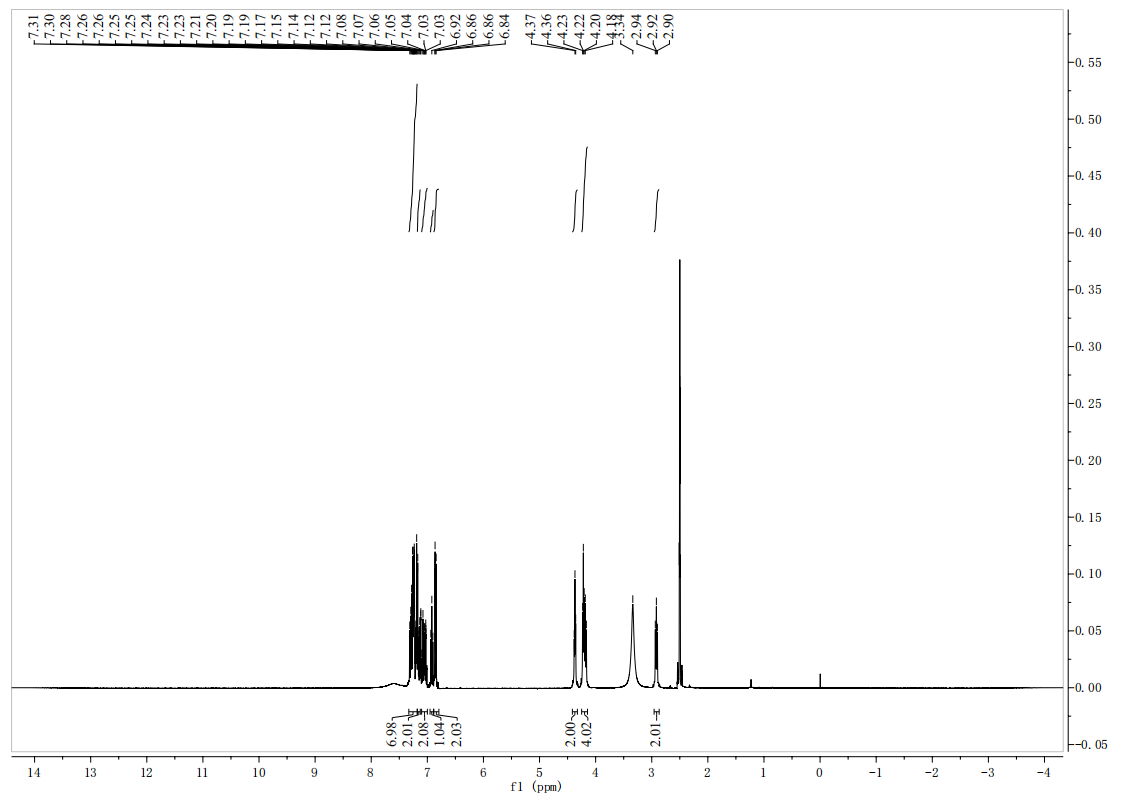

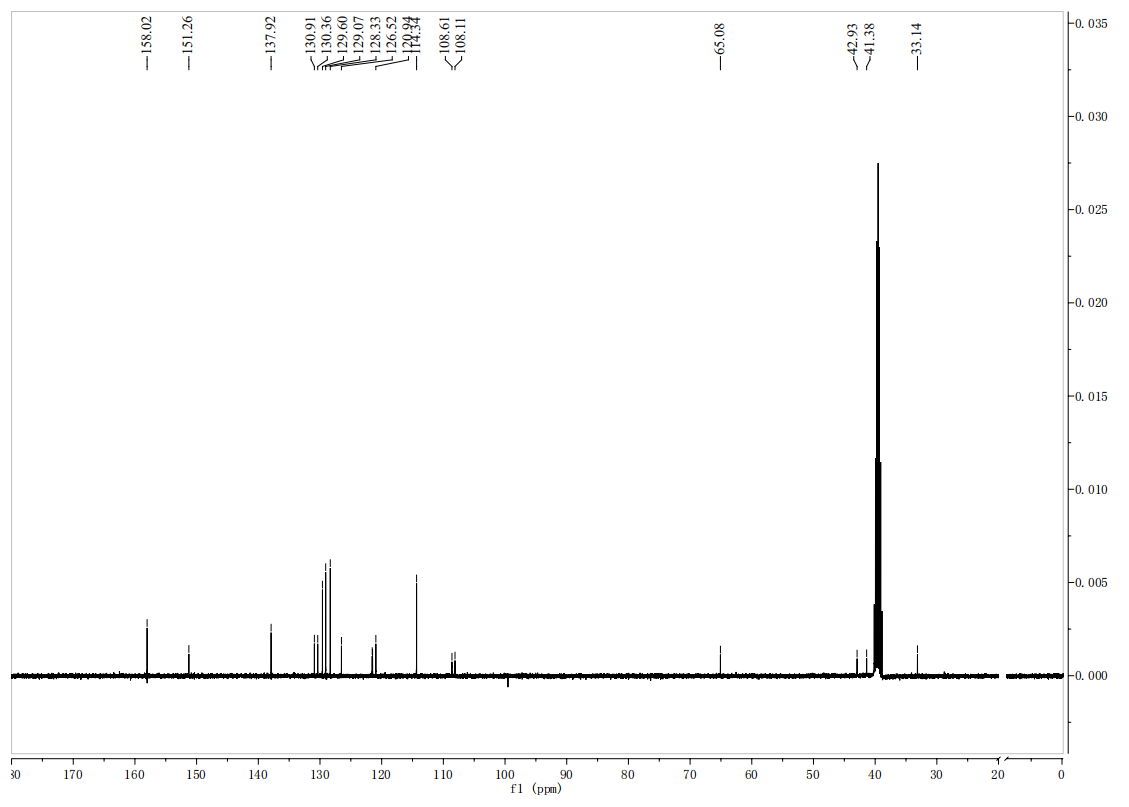


NMR spectra of compound **8l**
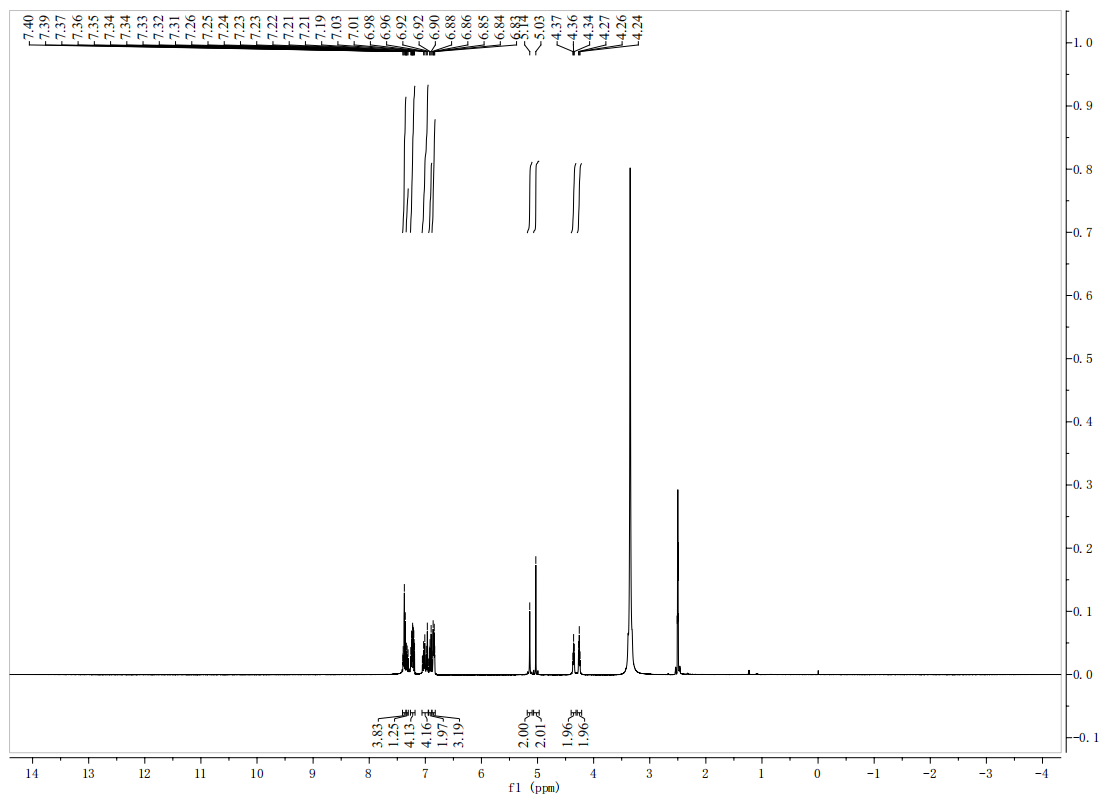

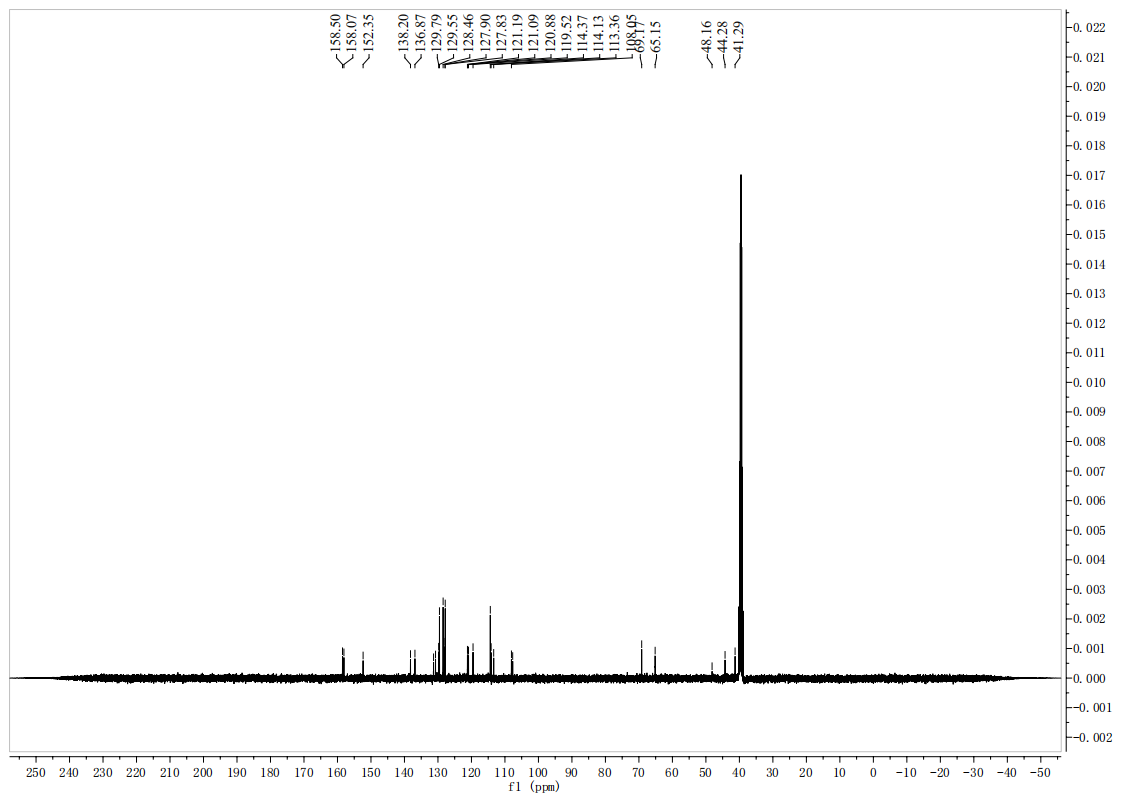


NMR spectra of compound **8m**
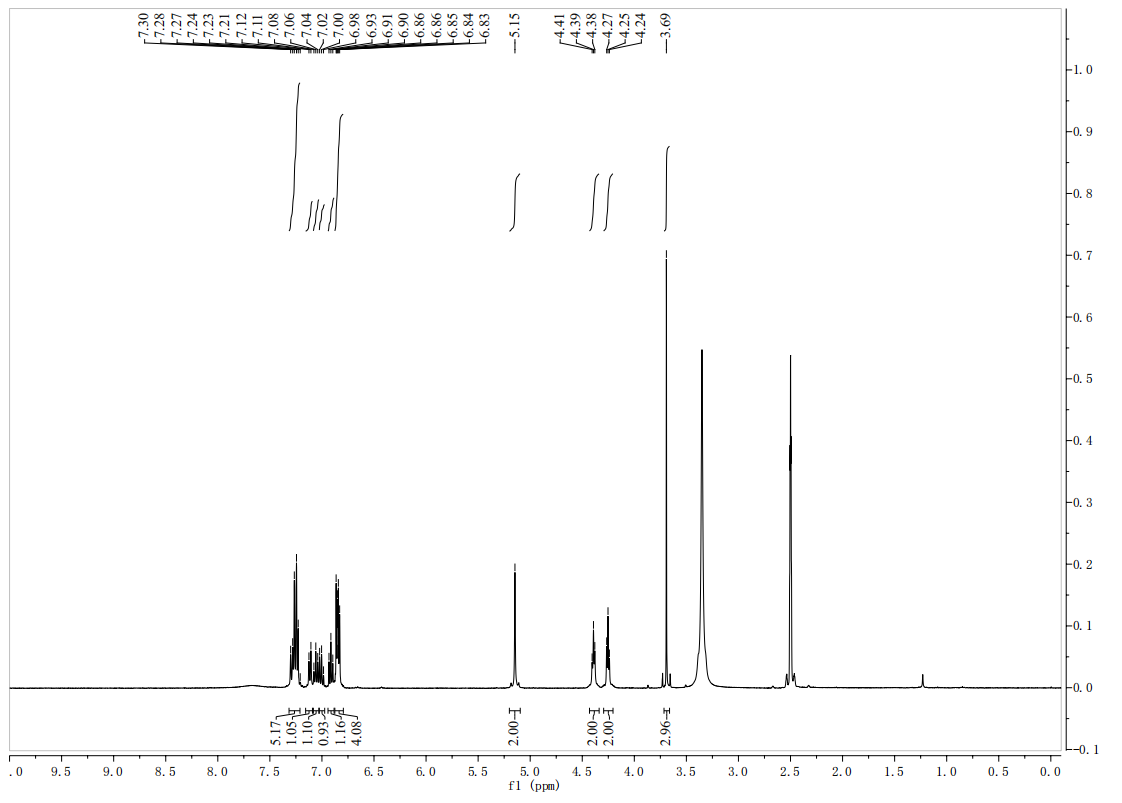

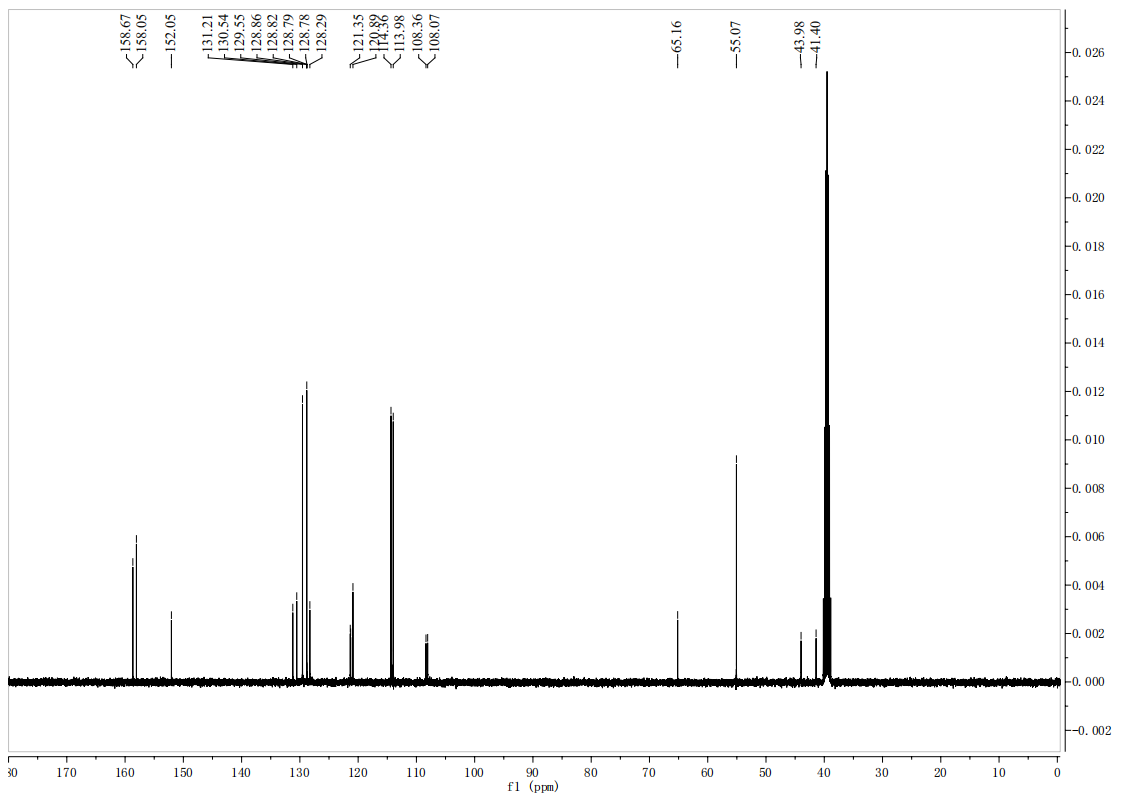


NMR spectra of compound **8n**
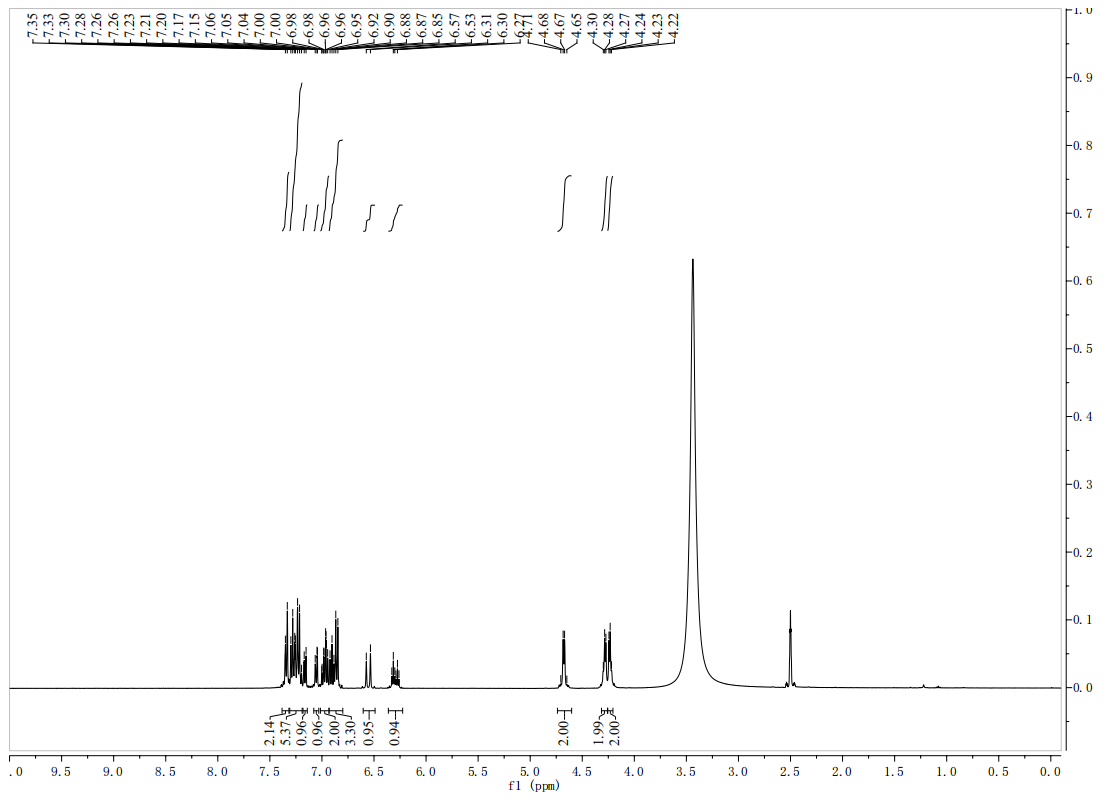

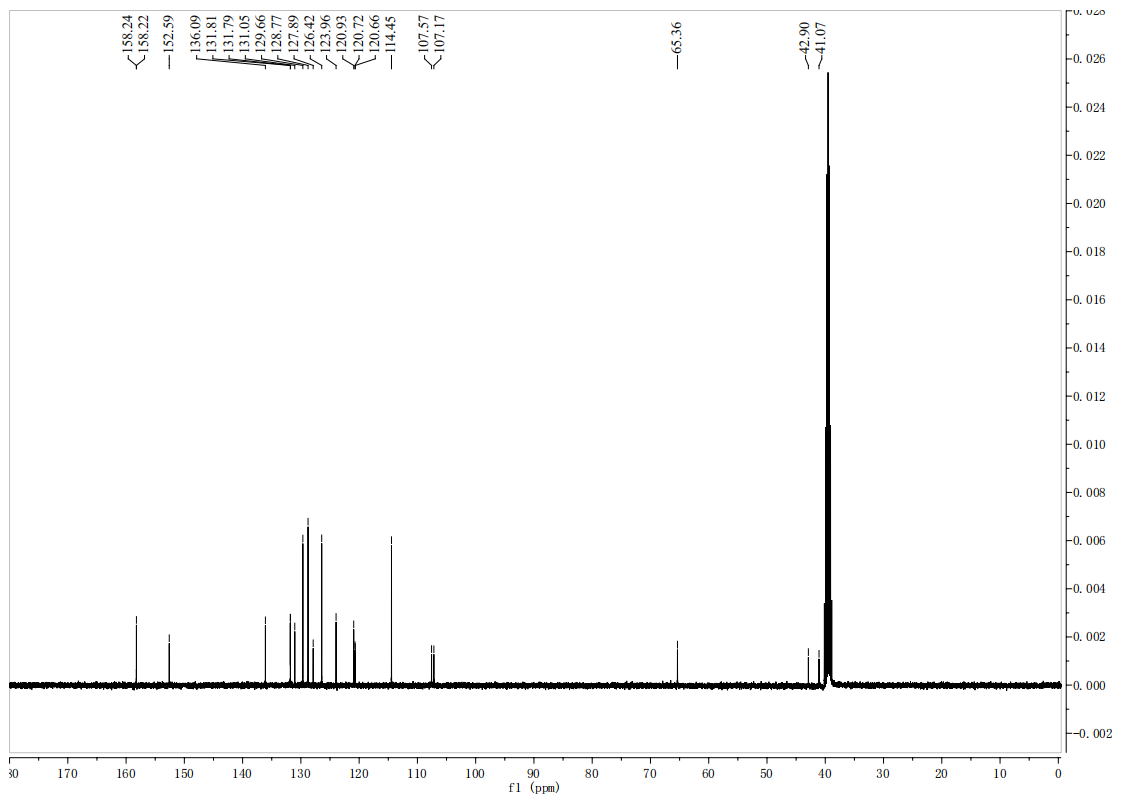


NMR spectra of compound **8o**
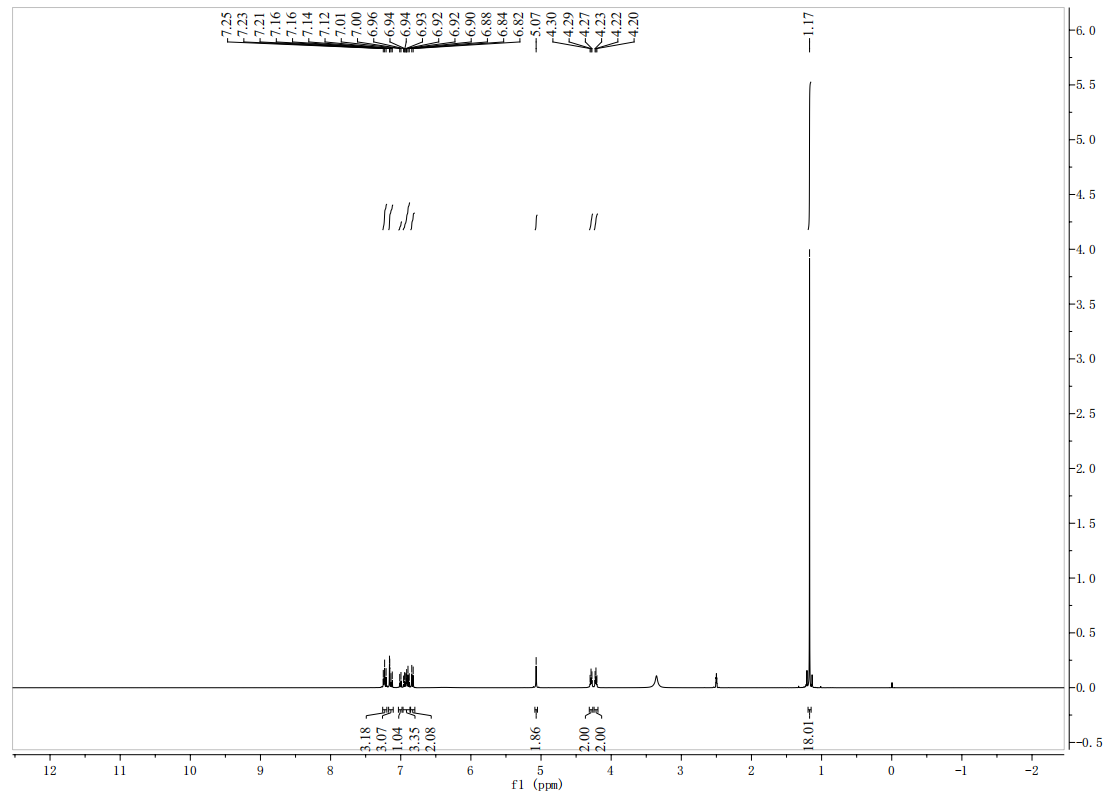

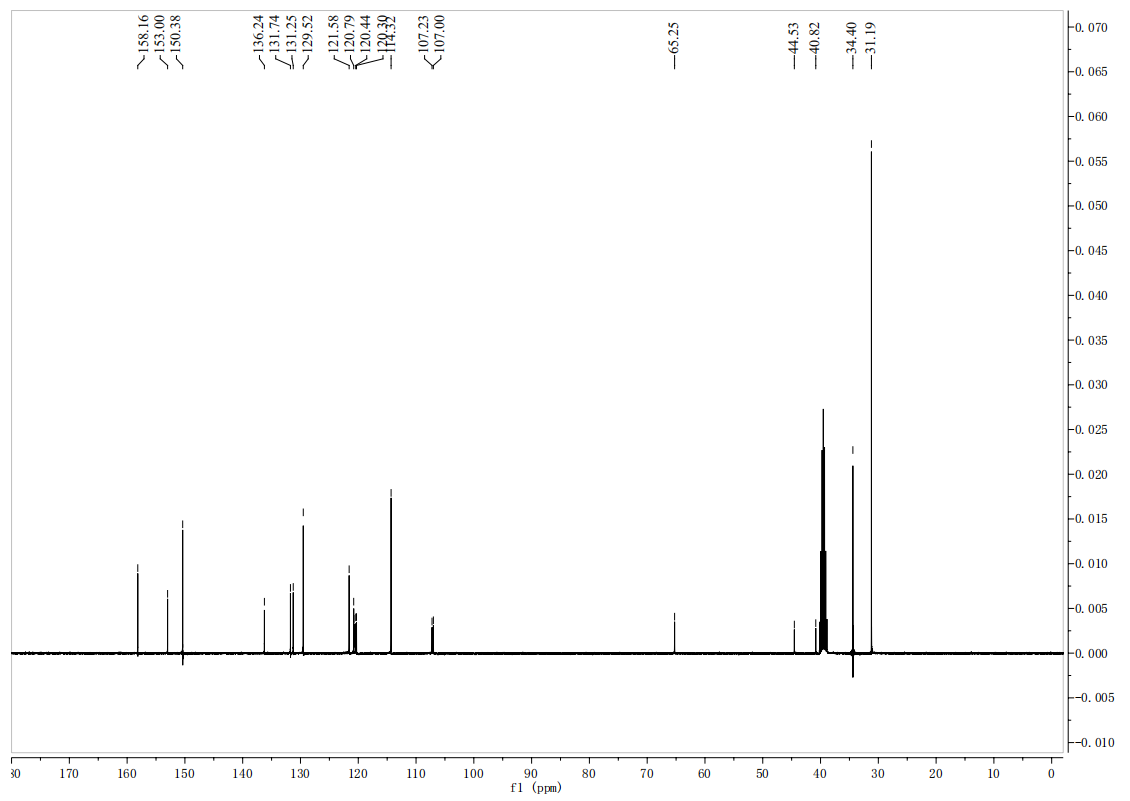


NMR spectra of compound **8p**
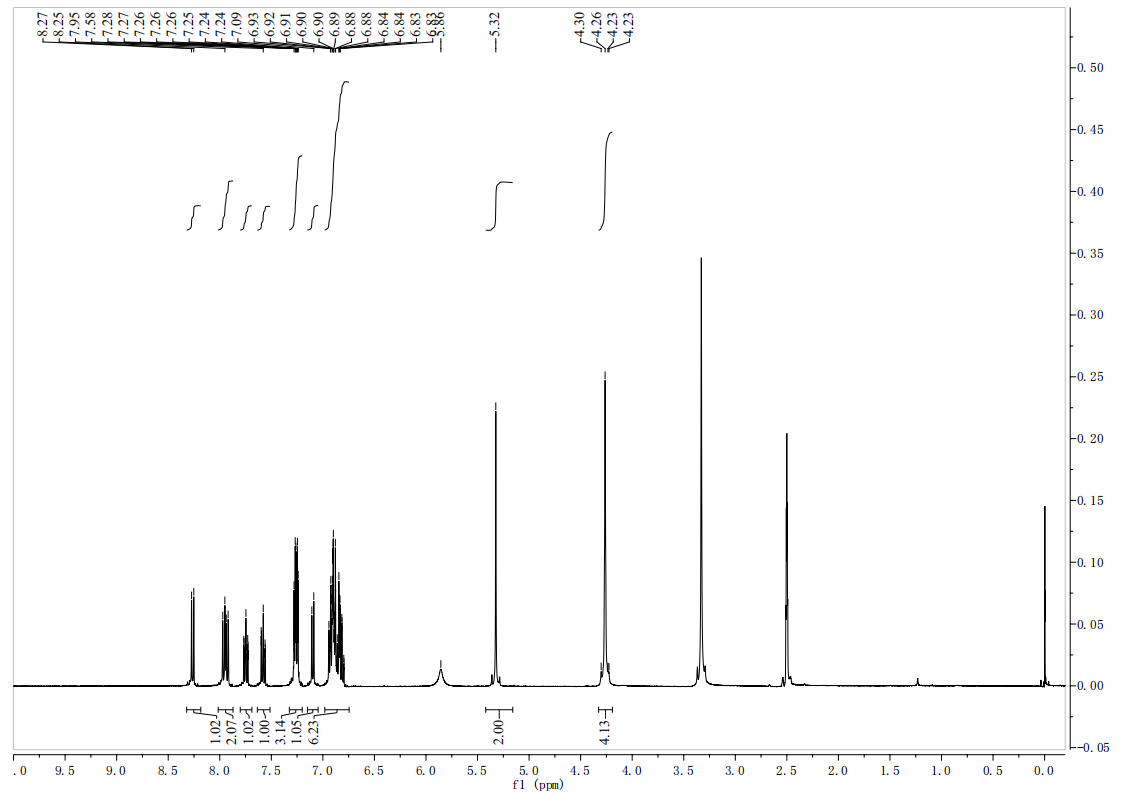

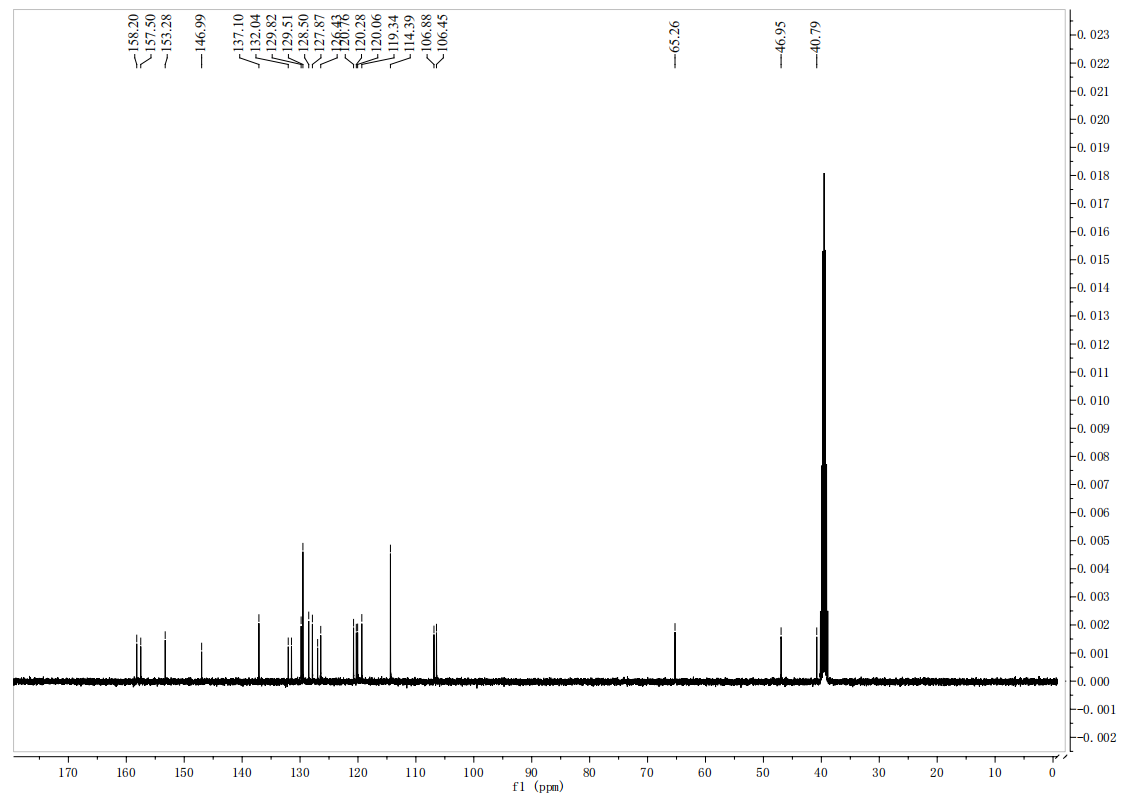


NMR spectra of compound **8q**
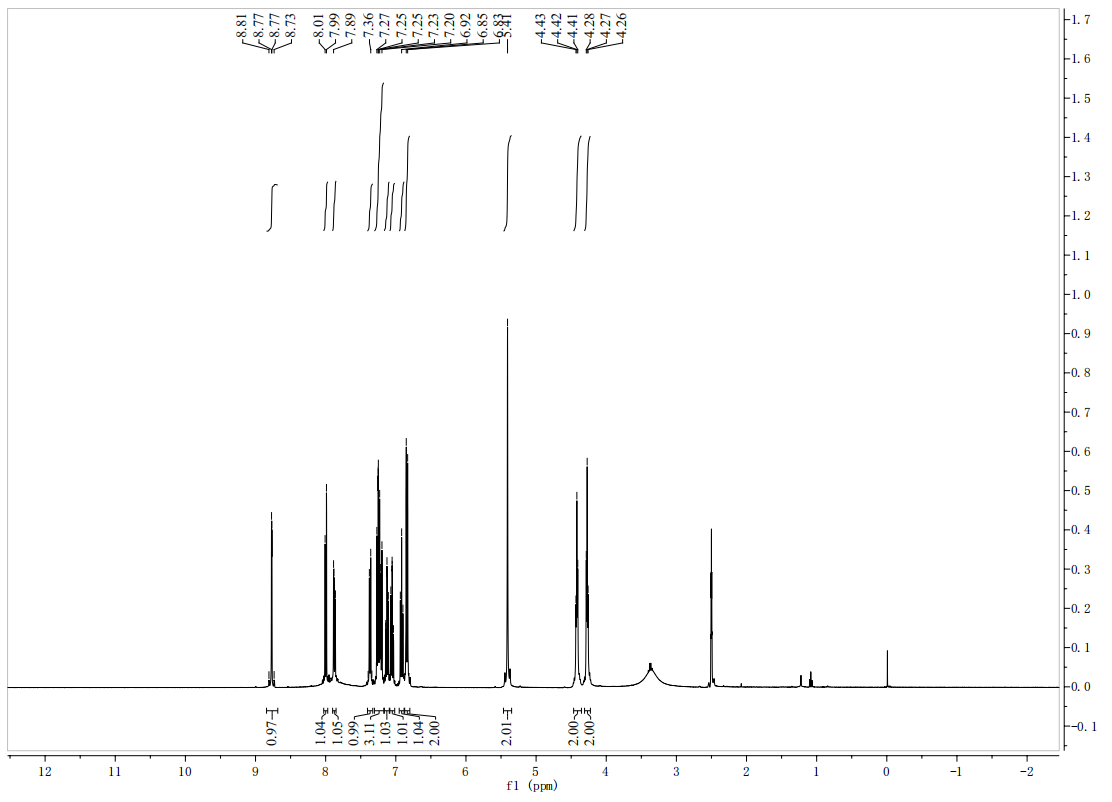

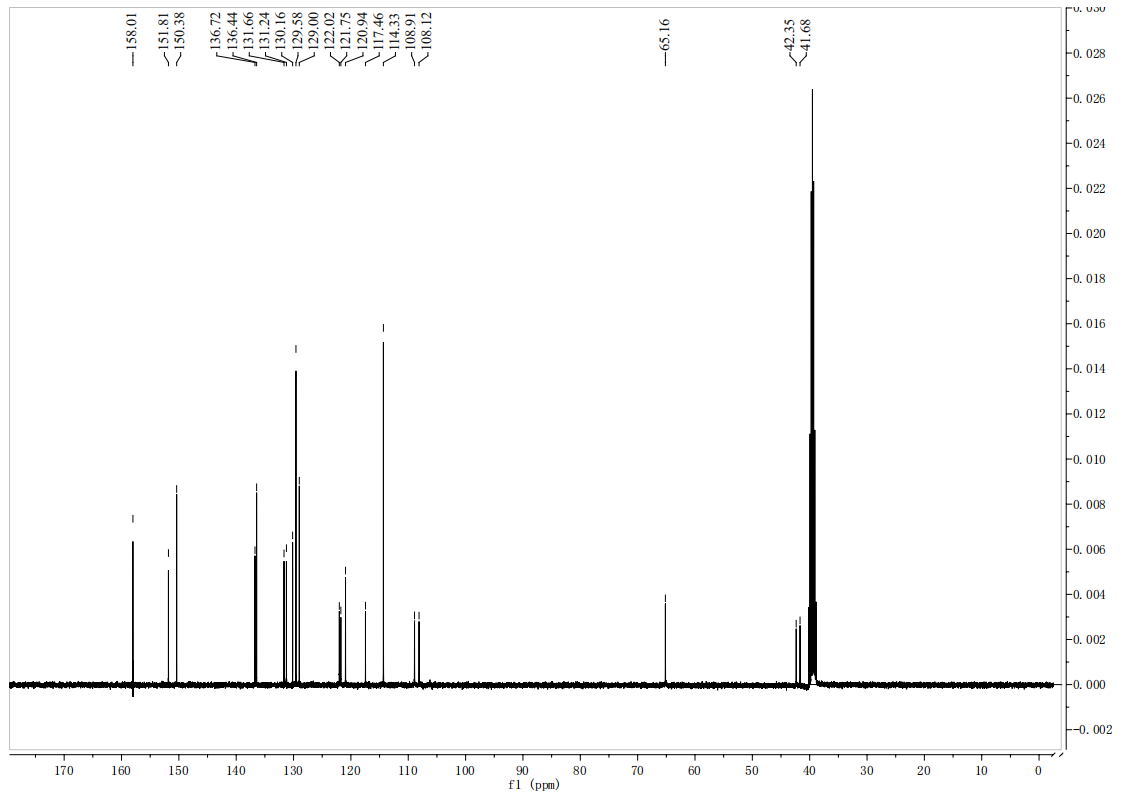


NMR spectra of compound **8r**
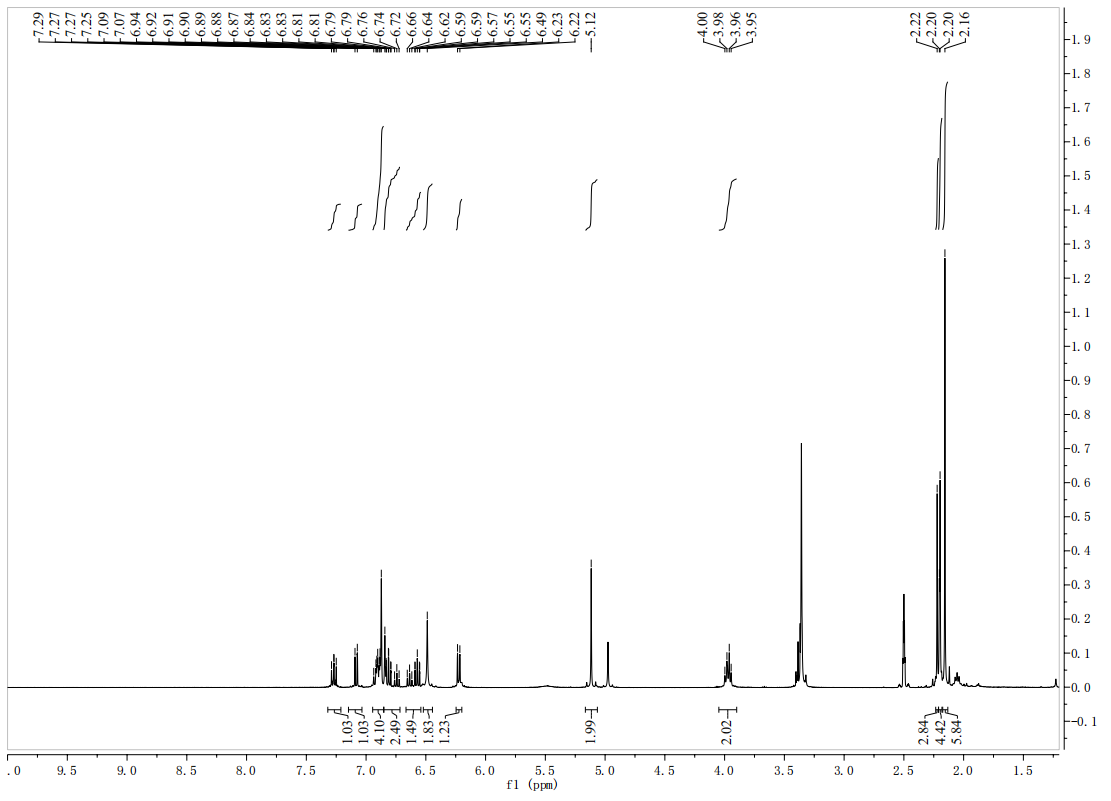

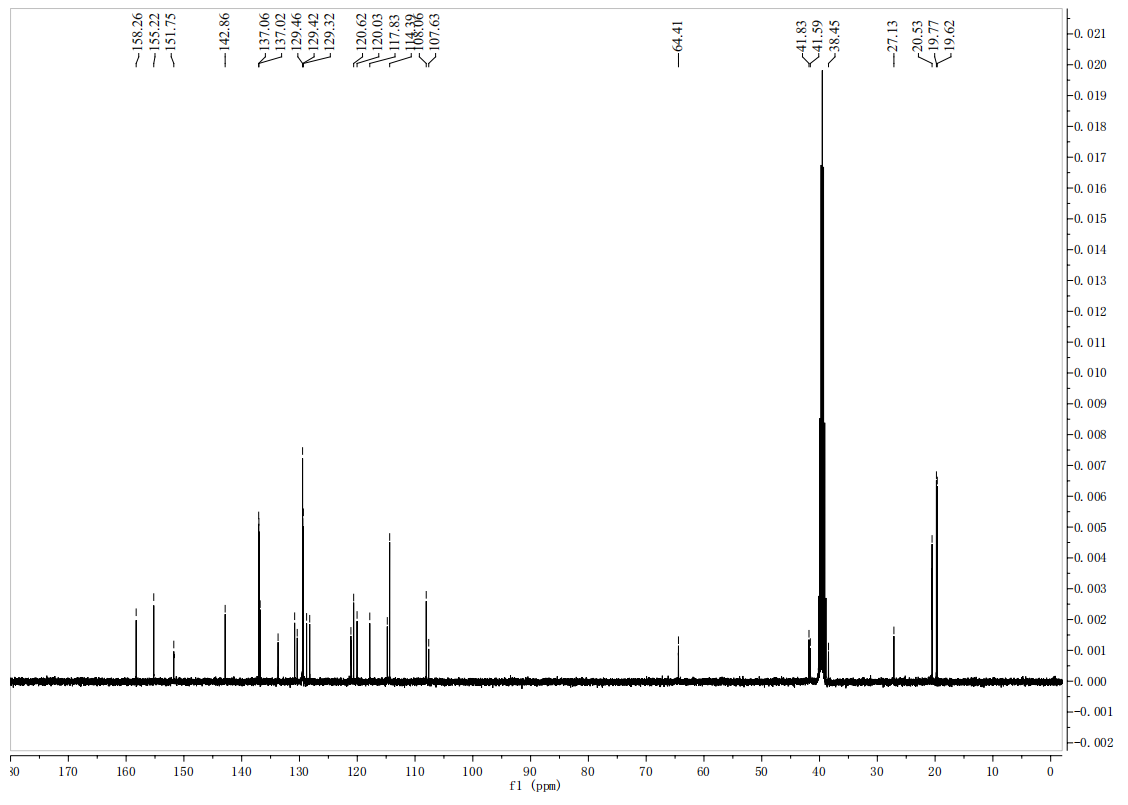


NMR spectra of compound **8s**
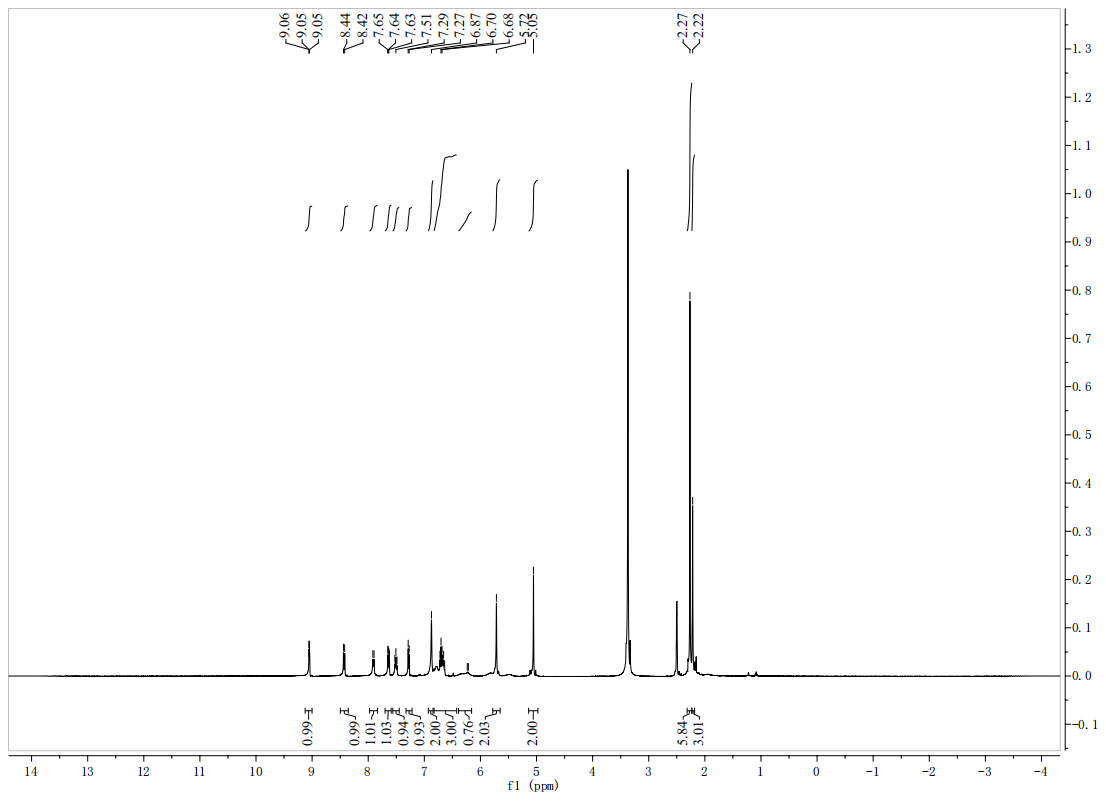

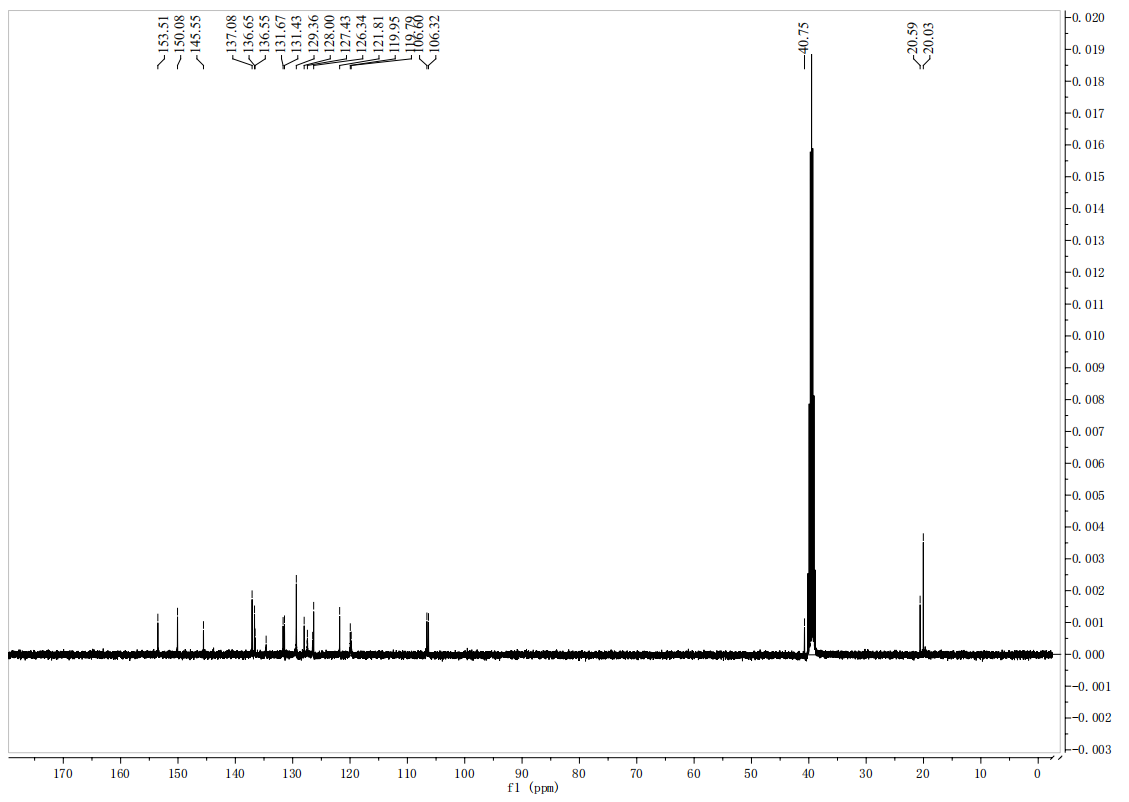


NMR spectra of compound **8t**
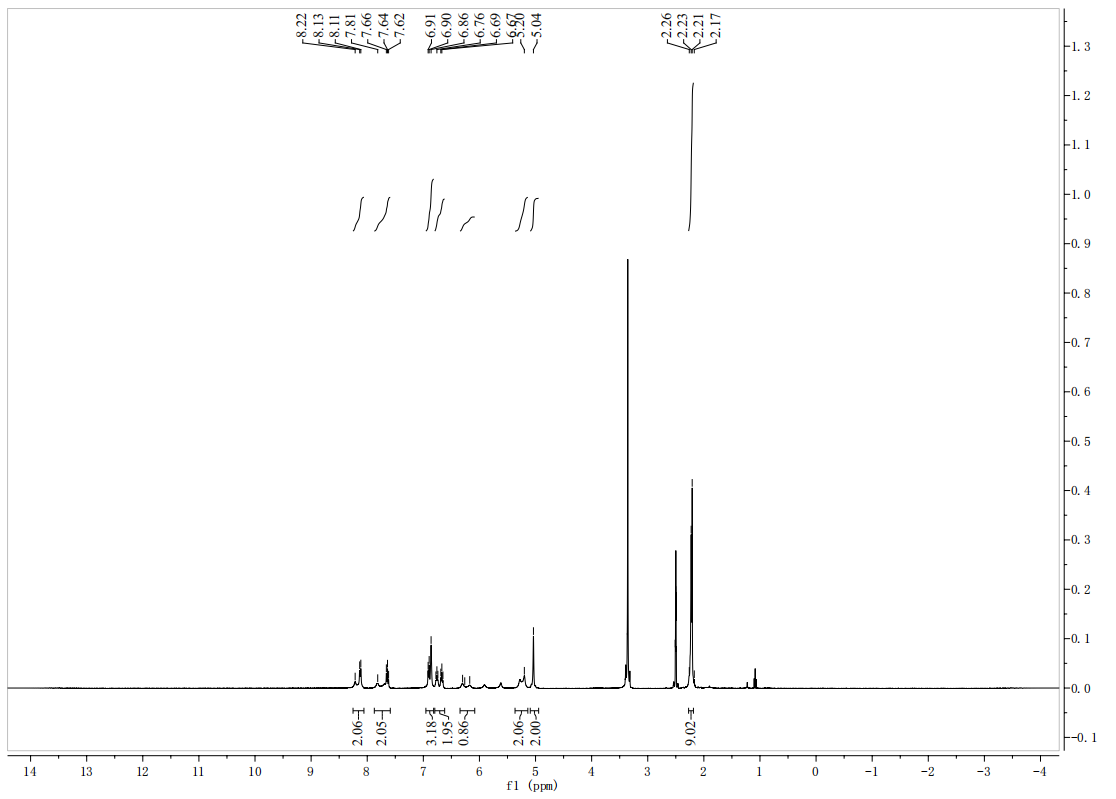

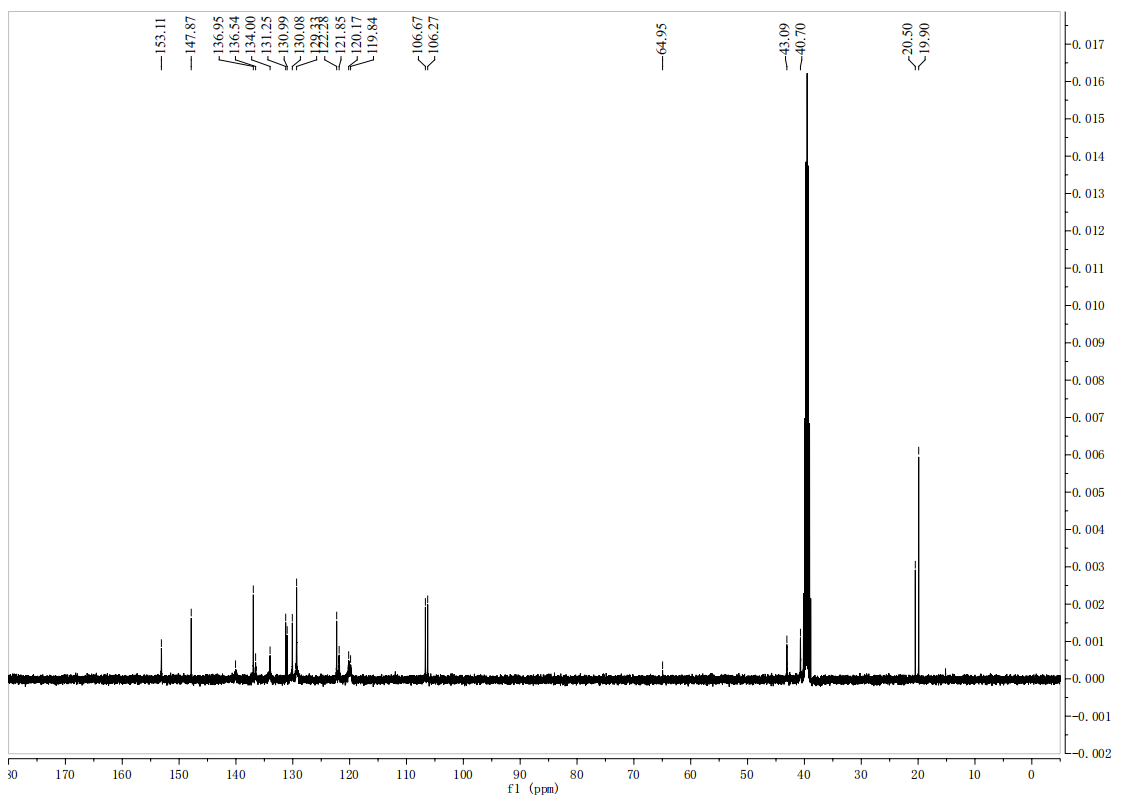


NMR spectra of compound **8u**
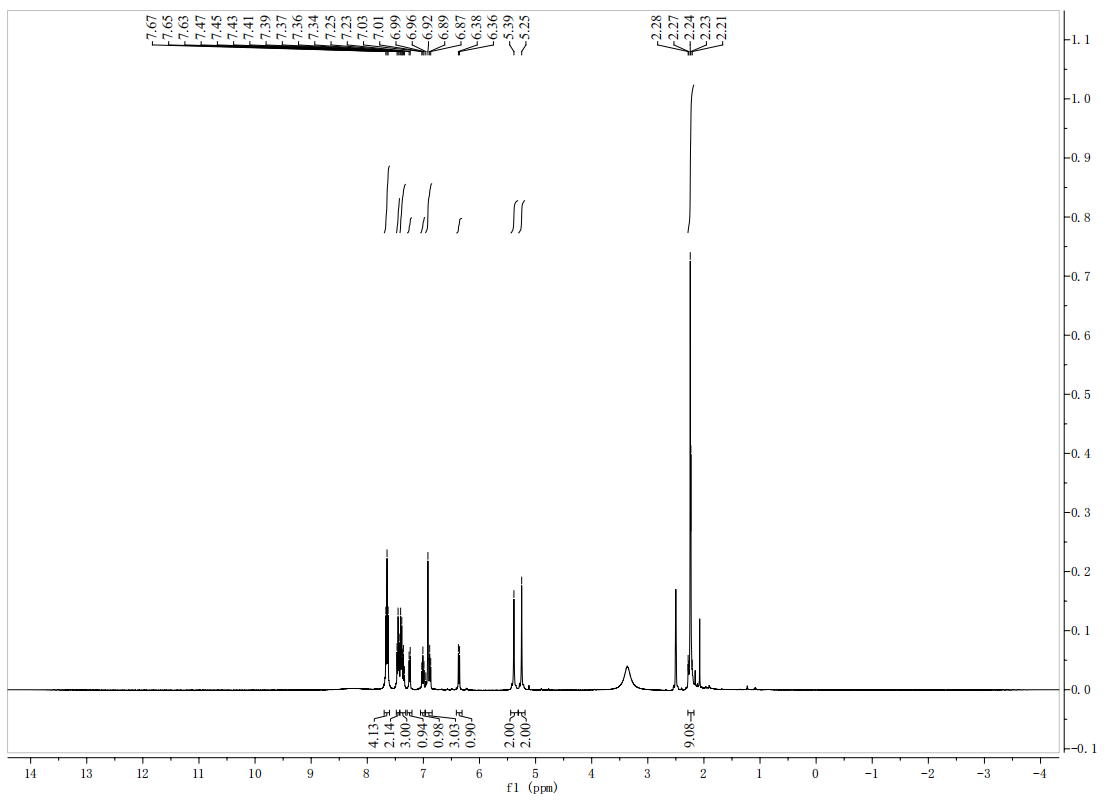

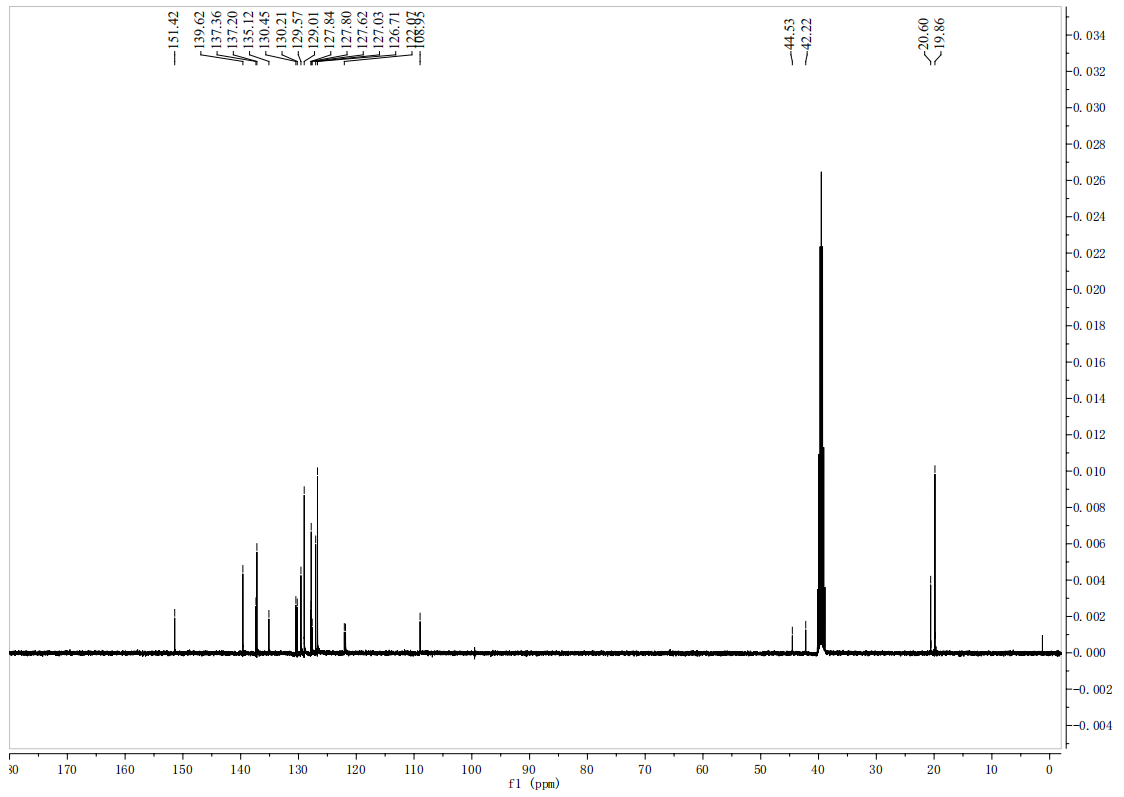


NMR spectra of compound **8v**
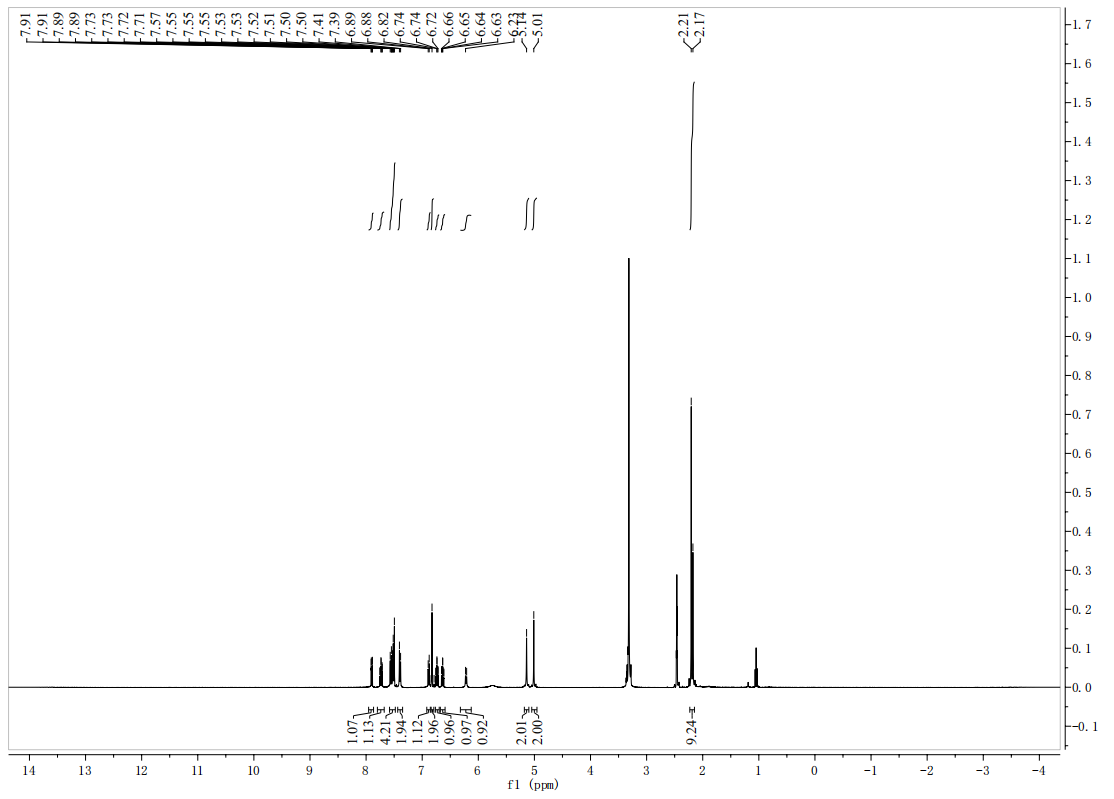

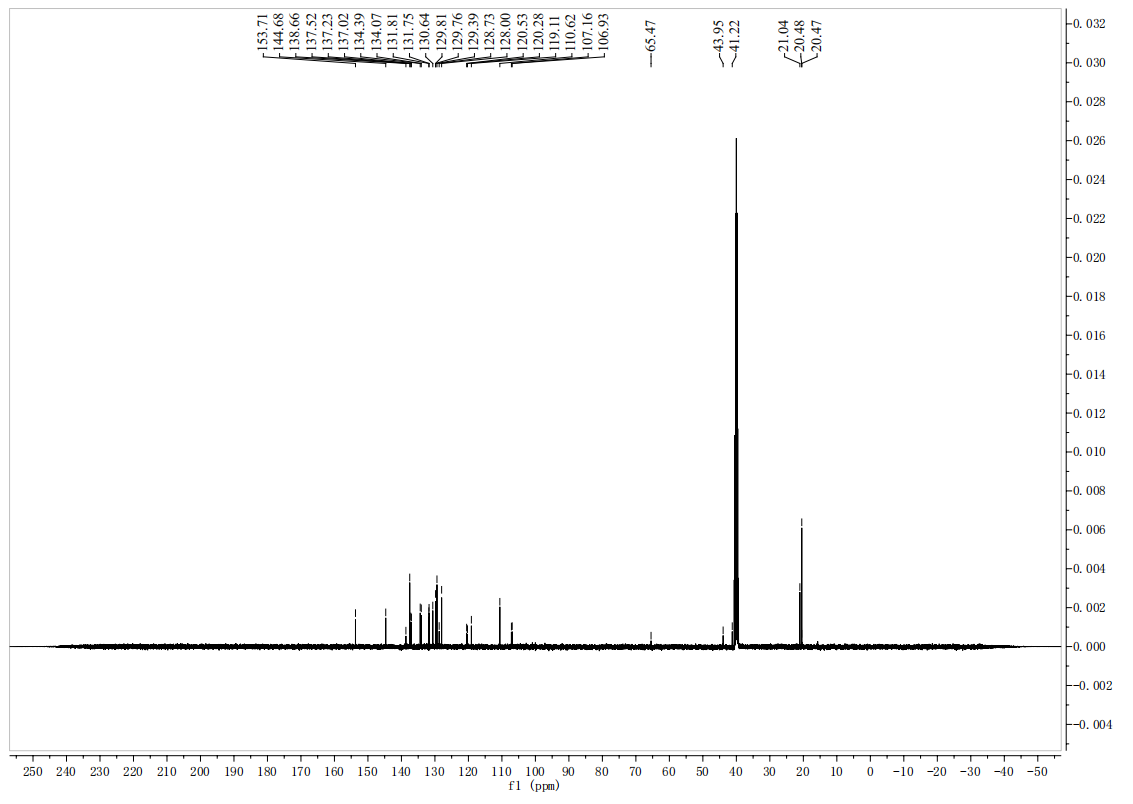


NMR spectra of compound **8w**
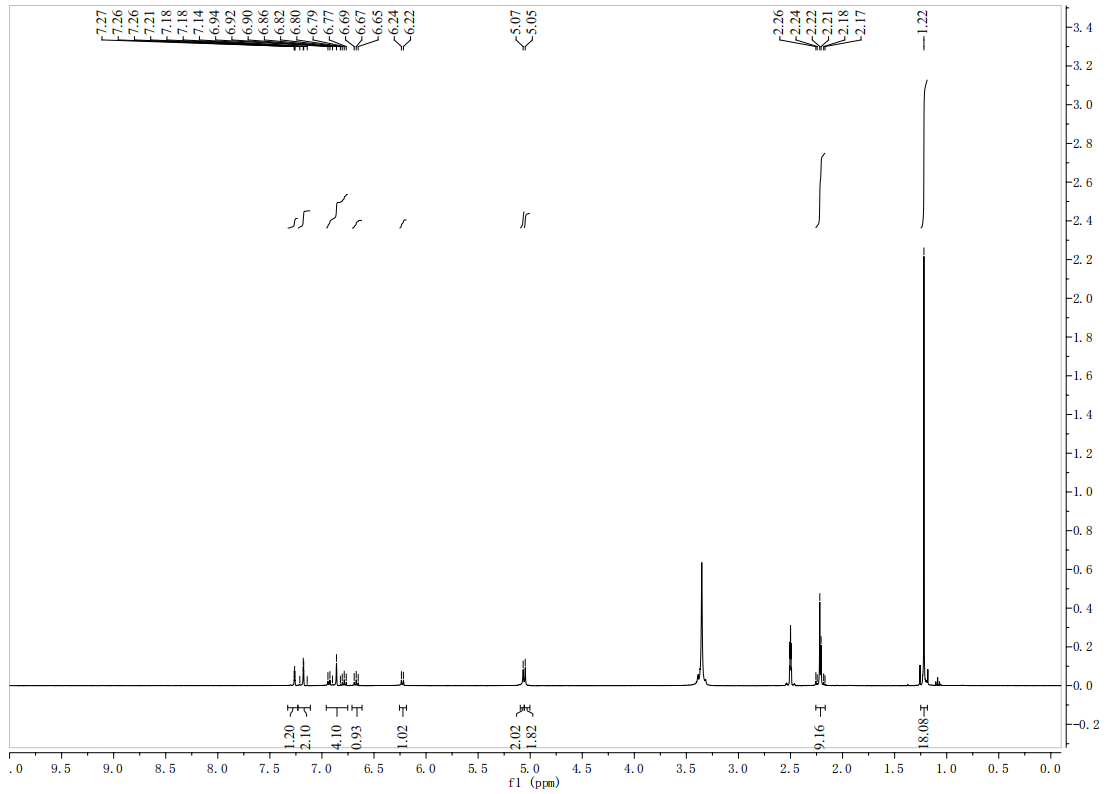

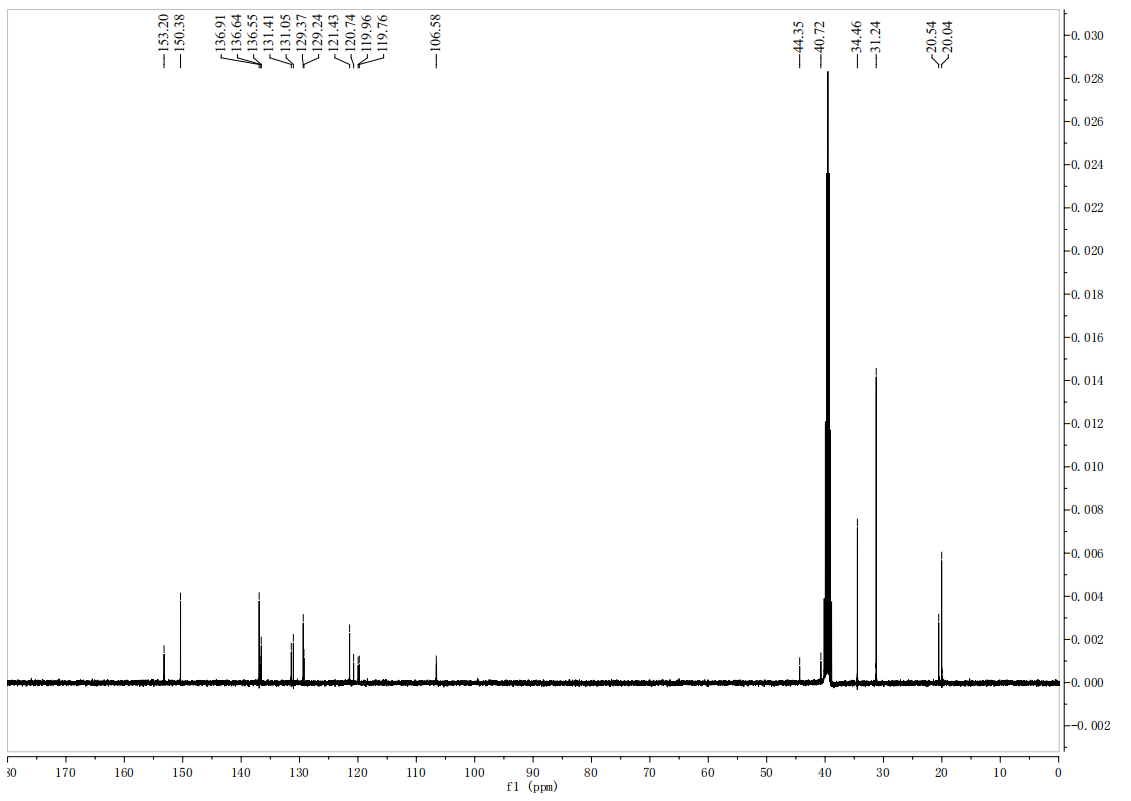


NMR spectra of compound **8x**
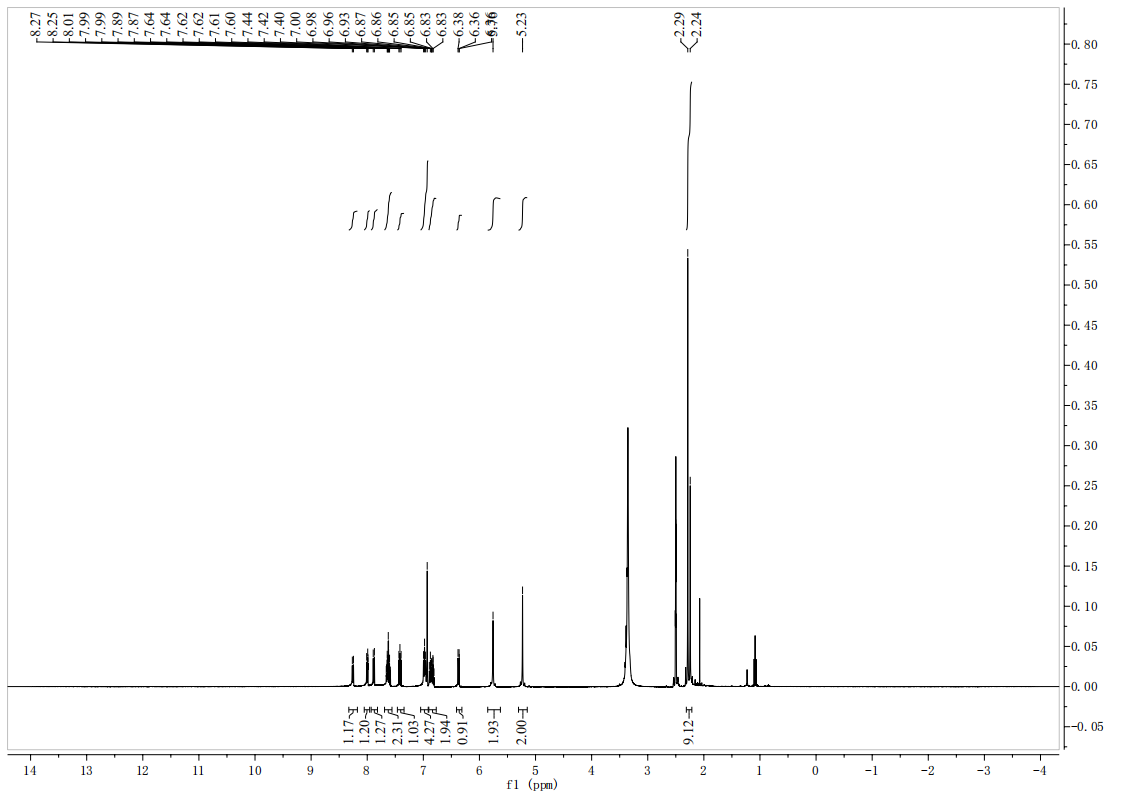

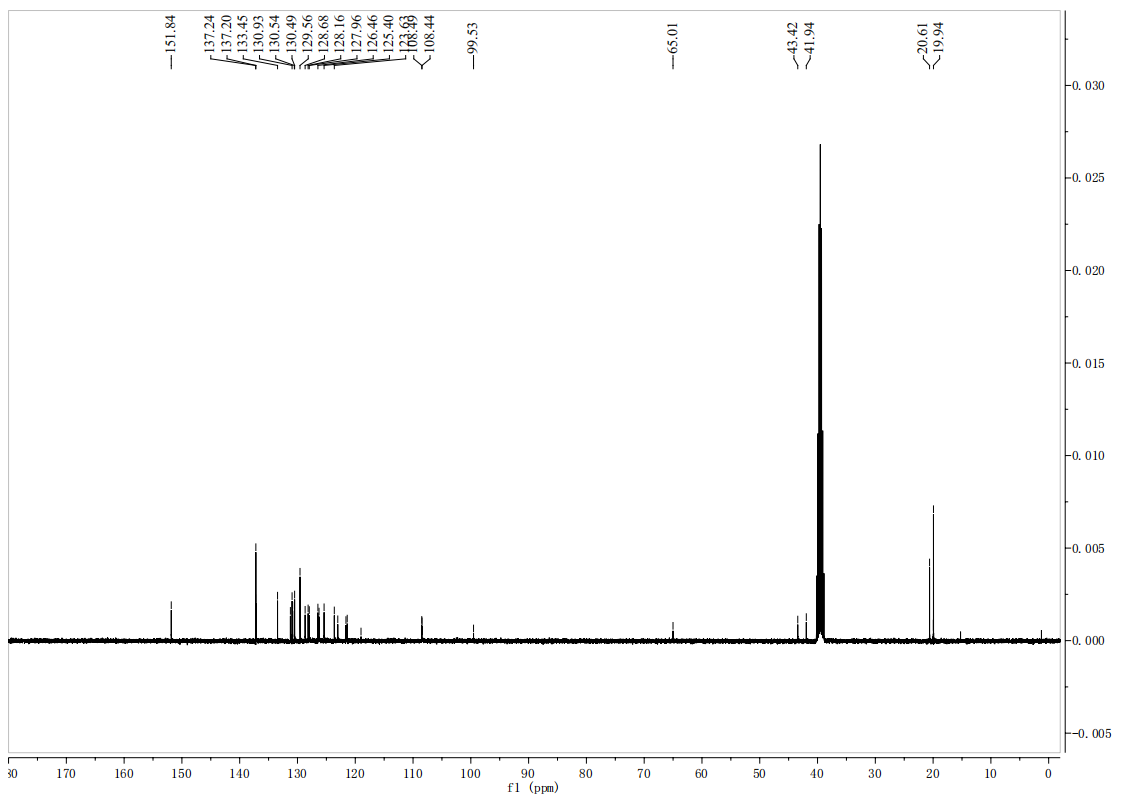


NMR spectra of compound **8y**
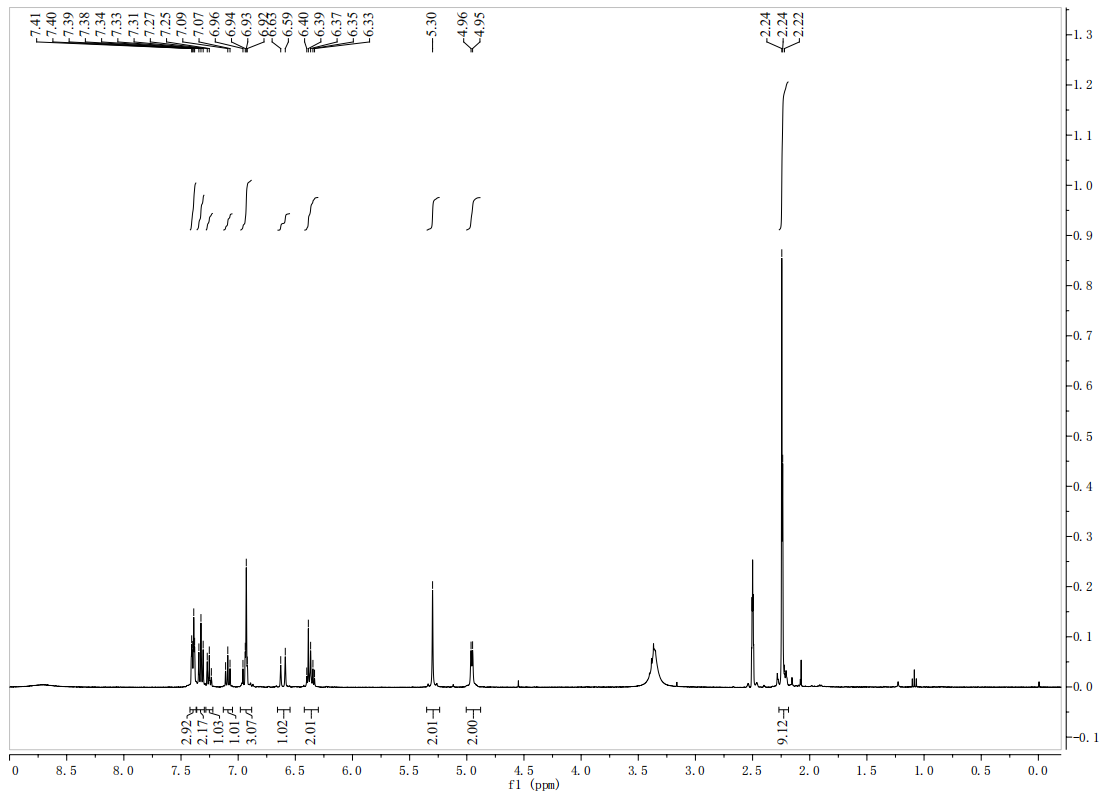

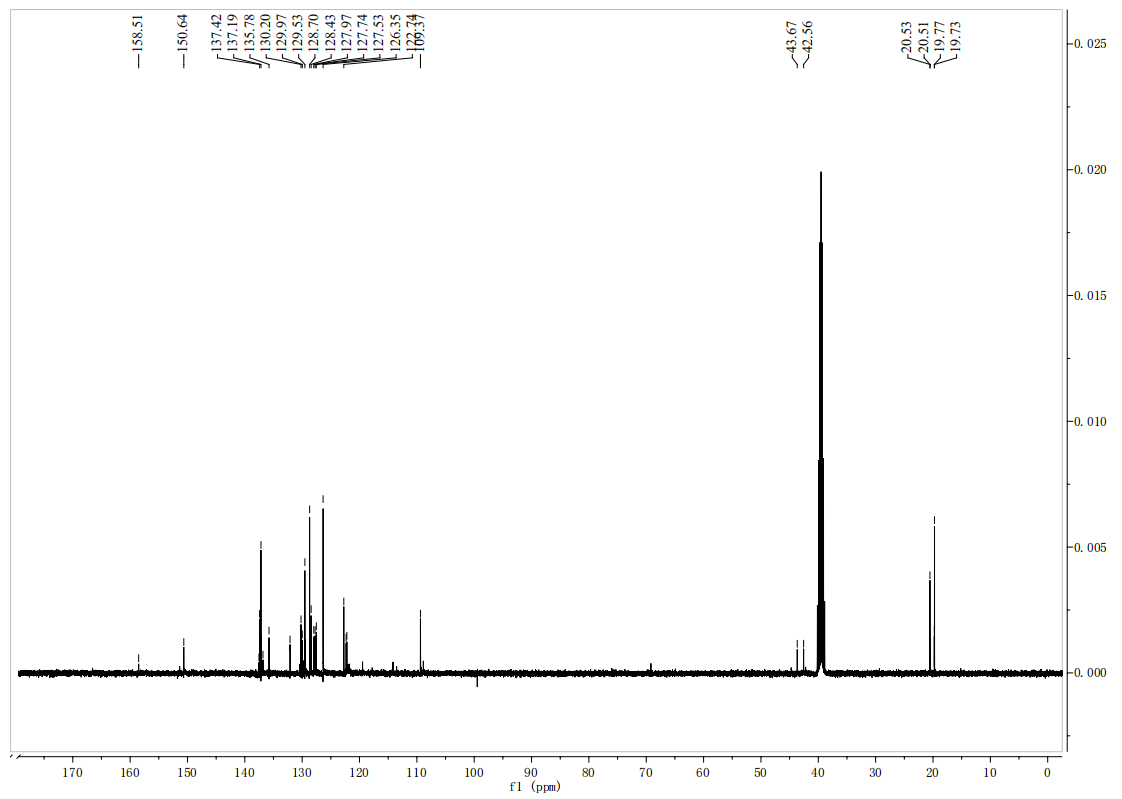


NMR spectra of compound **8z**
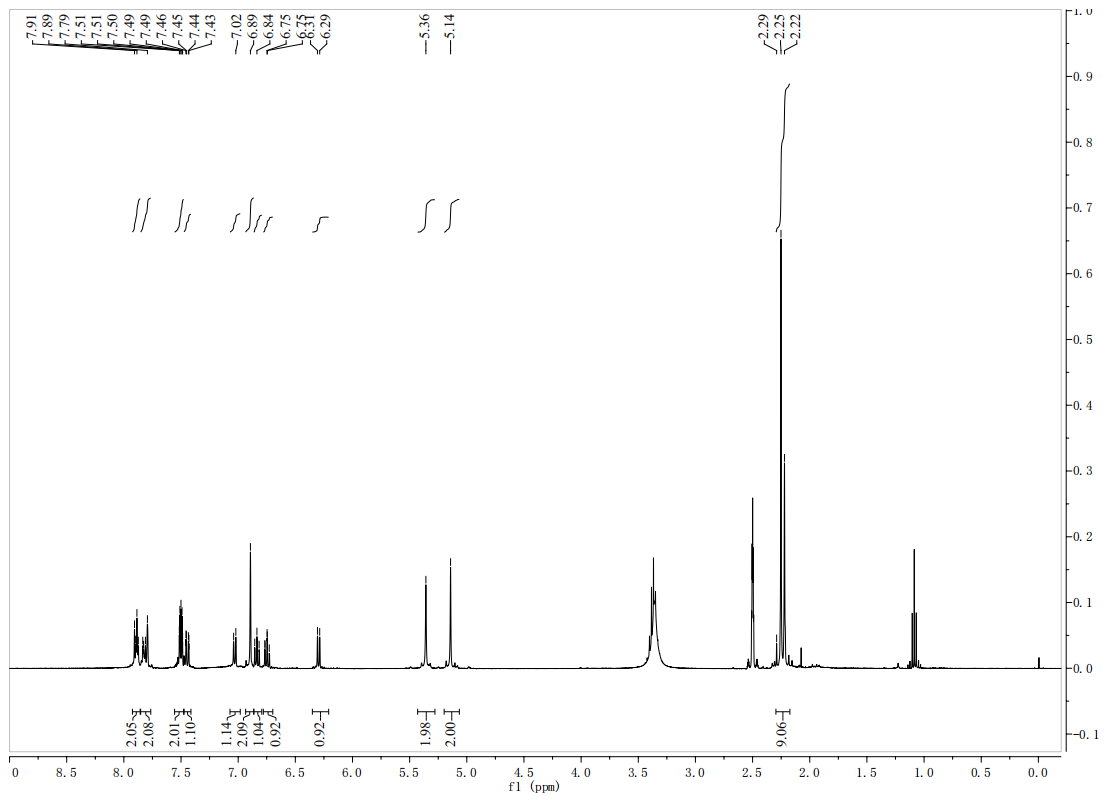

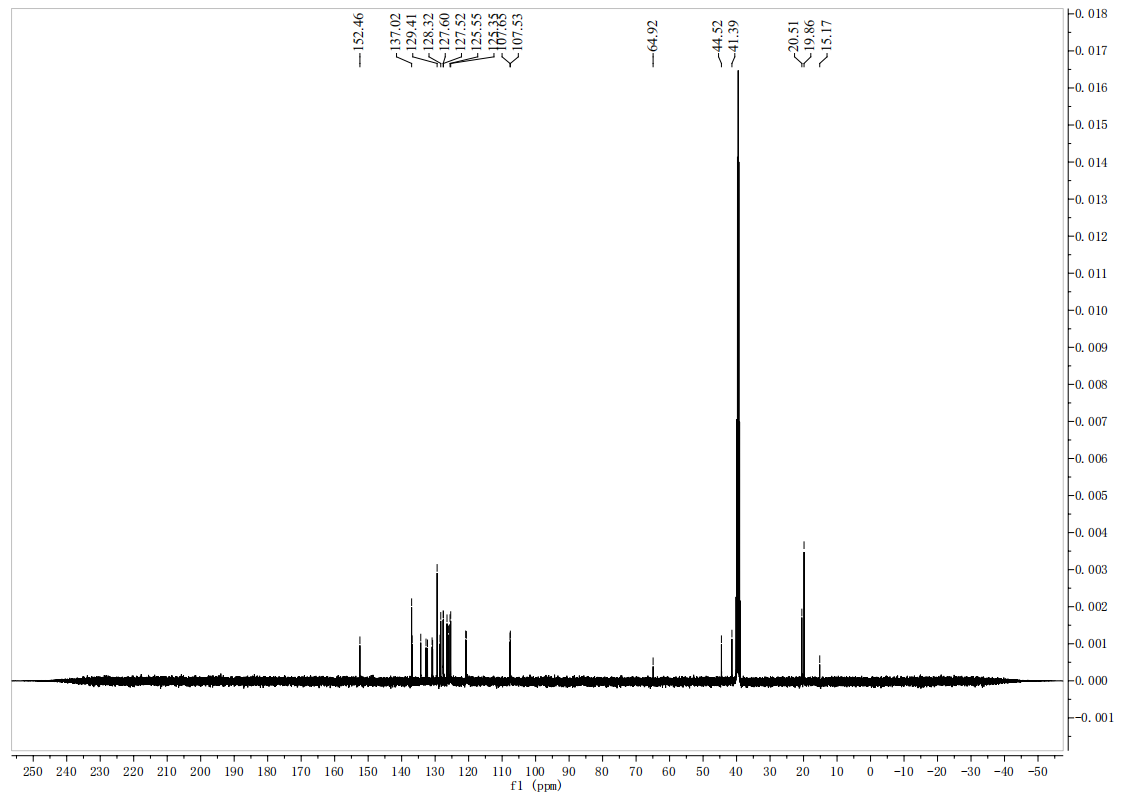


NMR spectra of compound **9a**
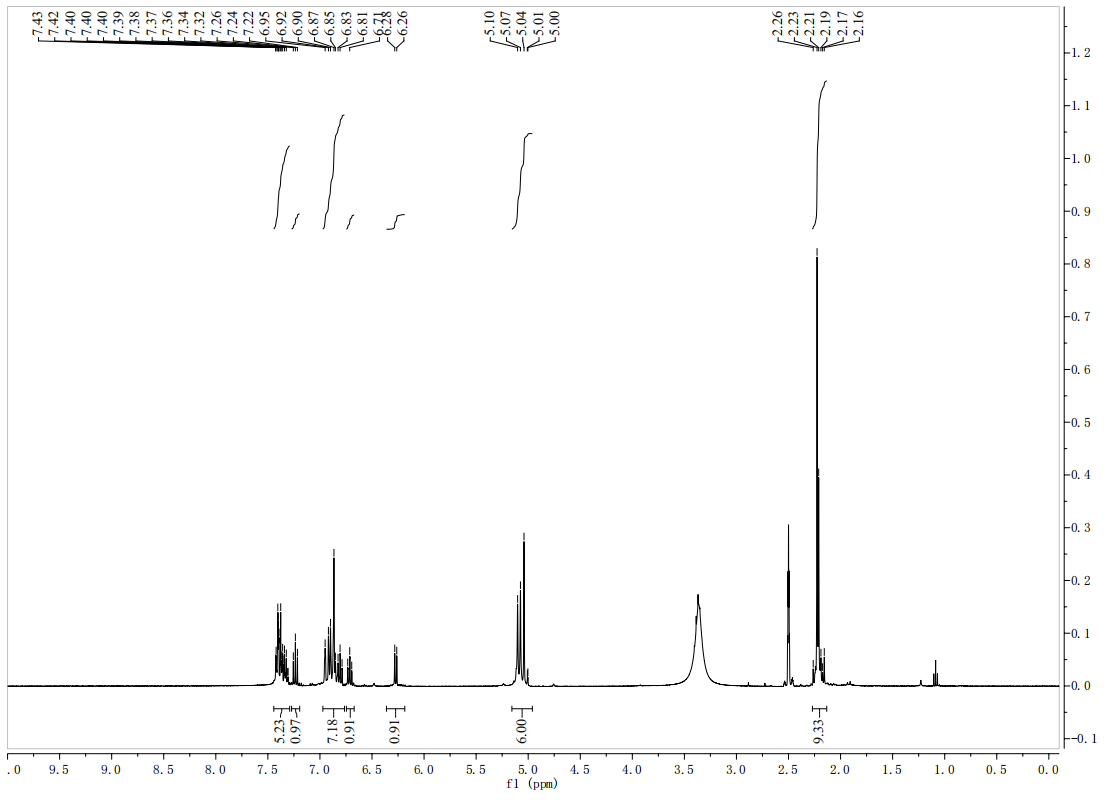

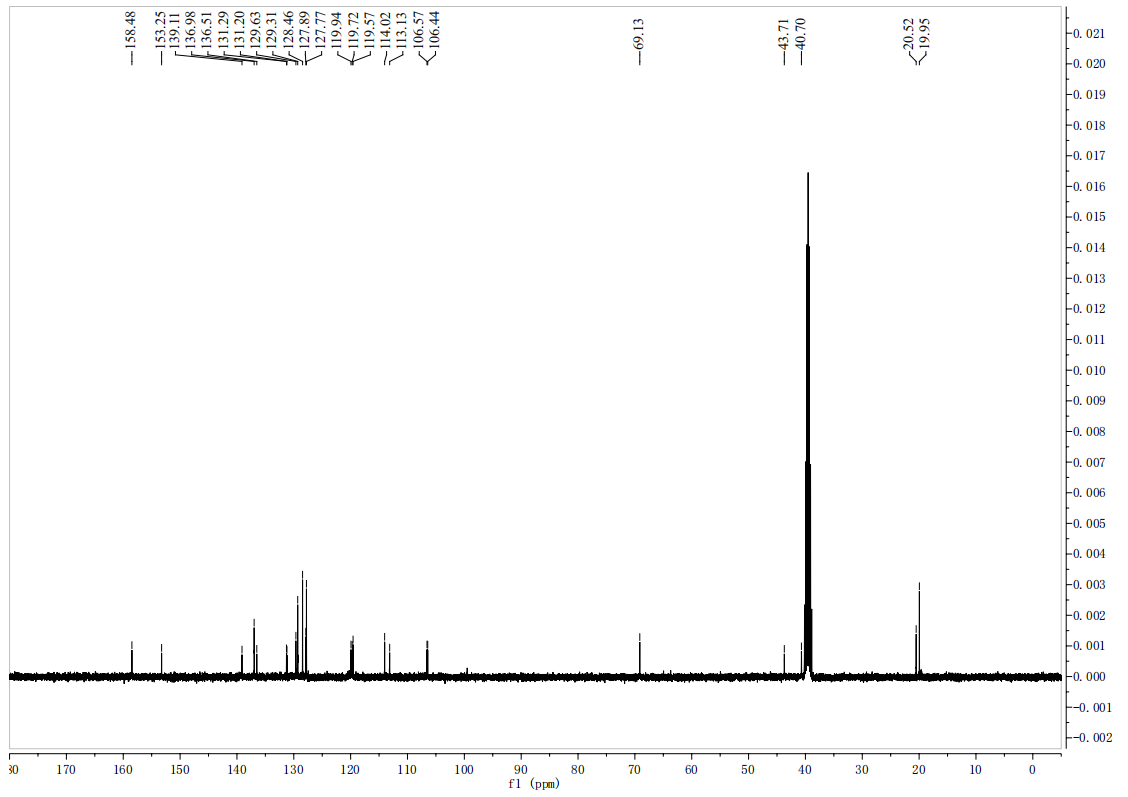


NMR spectra of compound **9b**

**2. ESI-HRMS (TOF) of final compounds**


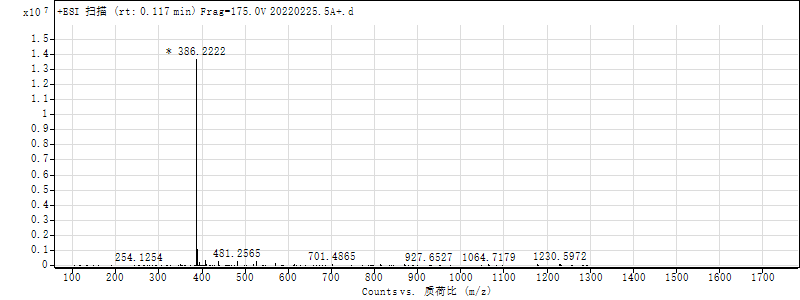


**Figure S35.** ESI-HRMS (TOF) of compound **5a**


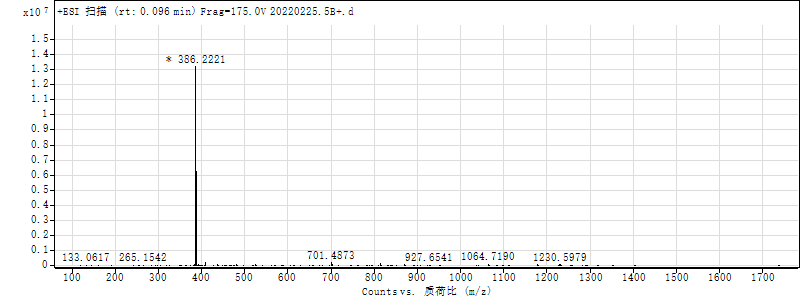


ESI-HRMS (TOF) of compound **5b**

**
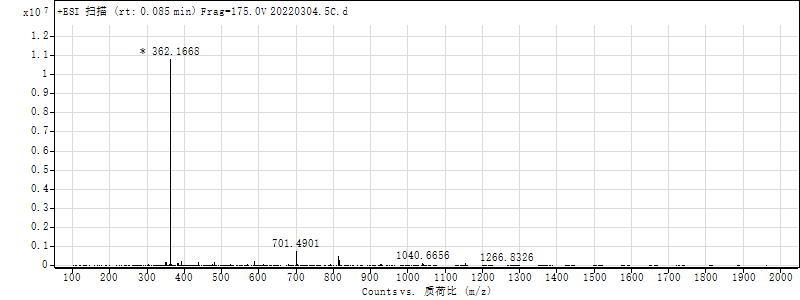
**

ESI-HRMS (TOF) of compound **5c**


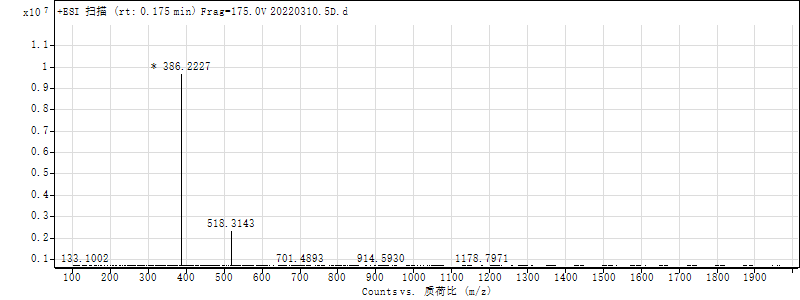


ESI-HRMS (TOF) of compound **5d**


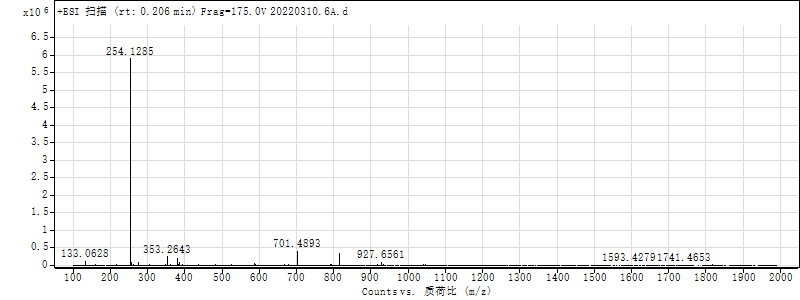


ESI-HRMS (TOF) of compound **6a**


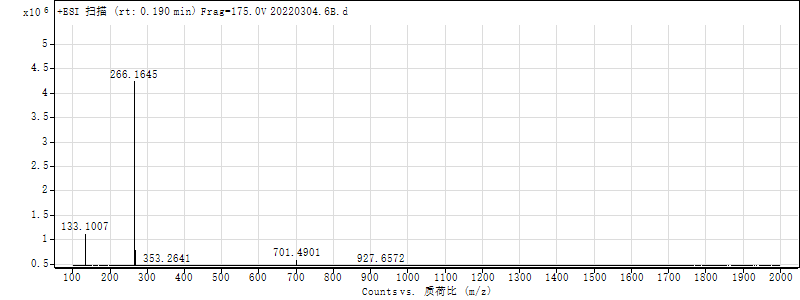


ESI-HRMS (TOF) of compound **6b**


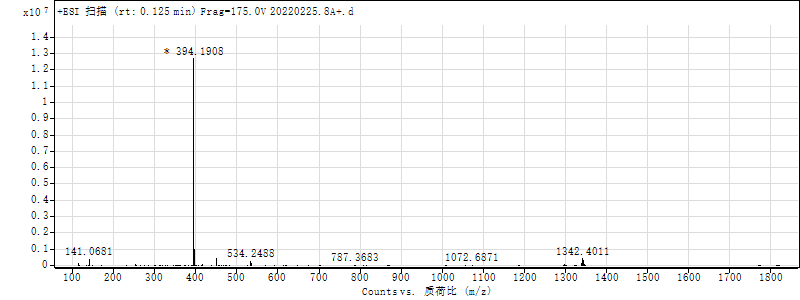


ESI-HRMS (TOF) of compound **8a**


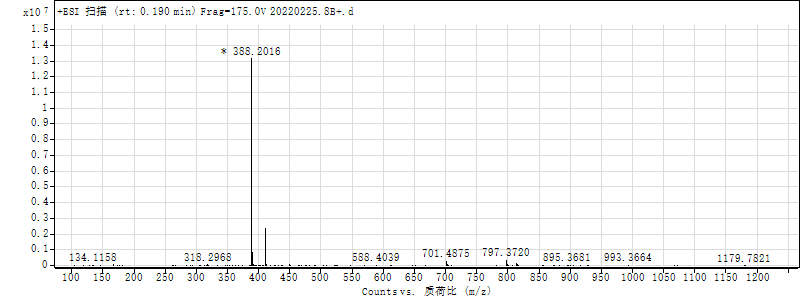


ESI-HRMS (TOF) of compound **8b**


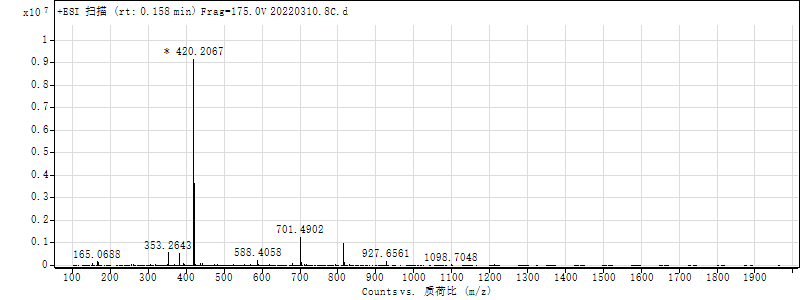


ESI-HRMS (TOF) of compound **8c**


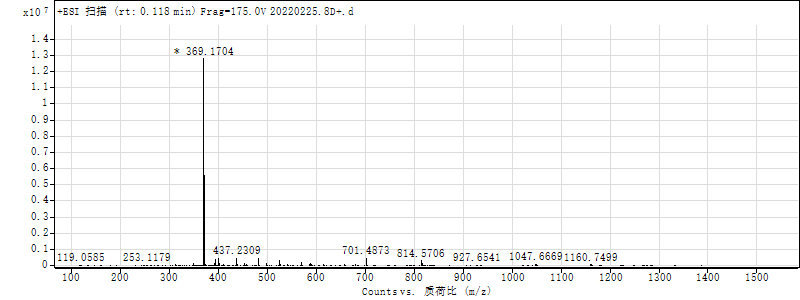


ESI-HRMS (TOF) of compound **8d**


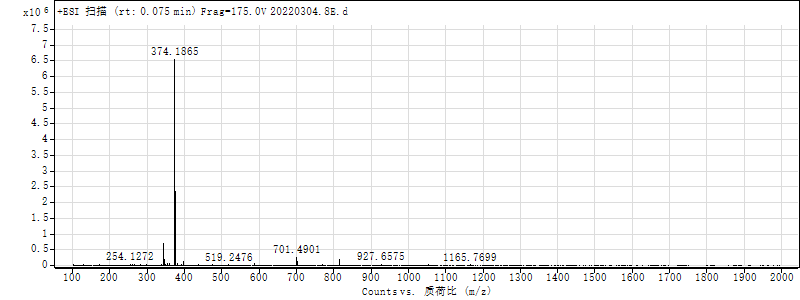


ESI-HRMS (TOF) of compound **8e**


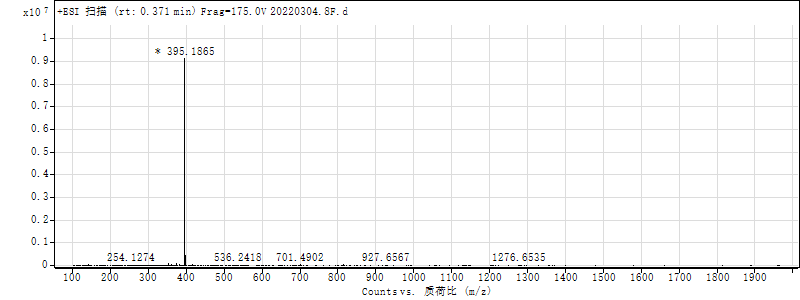


ESI-HRMS (TOF) of compound **8f**


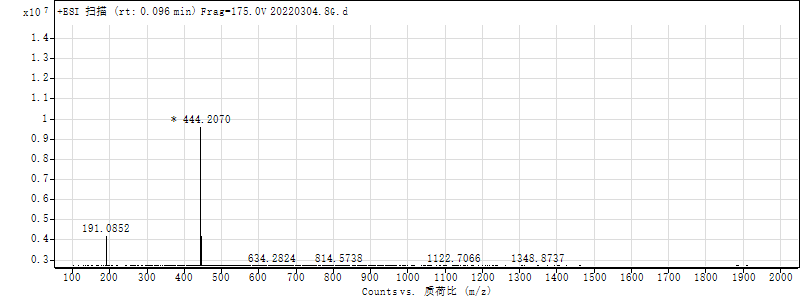


ESI-HRMS (TOF) of compound **8g**


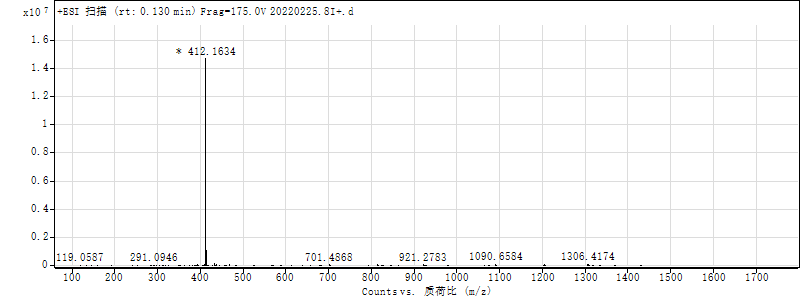


ESI-HRMS (TOF) of compound **8h**


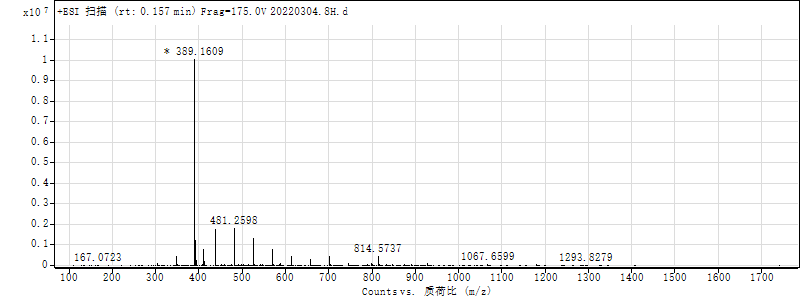


ESI-HRMS (TOF) of compound **8i**


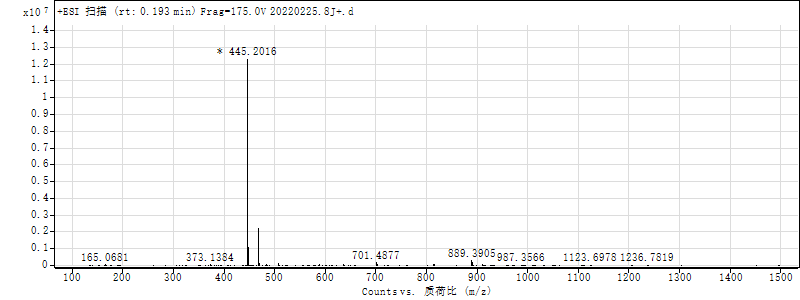


ESI-HRMS (TOF) of compound **8j**


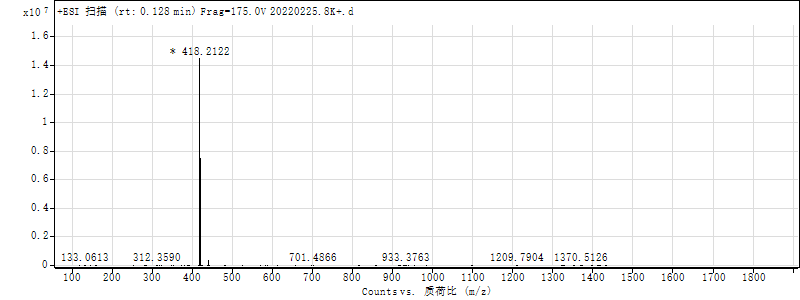


ESI-HRMS (TOF) of compound **8k**


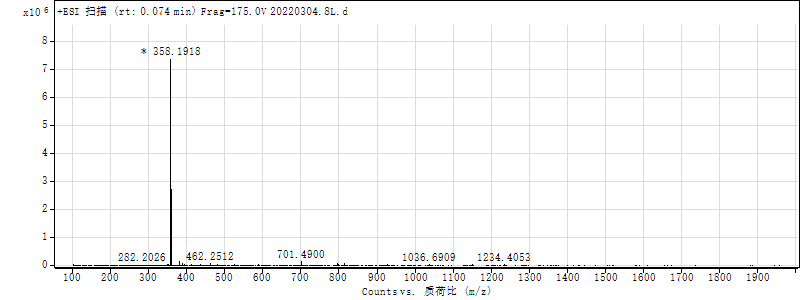


ESI-HRMS (TOF) of compound **8l**


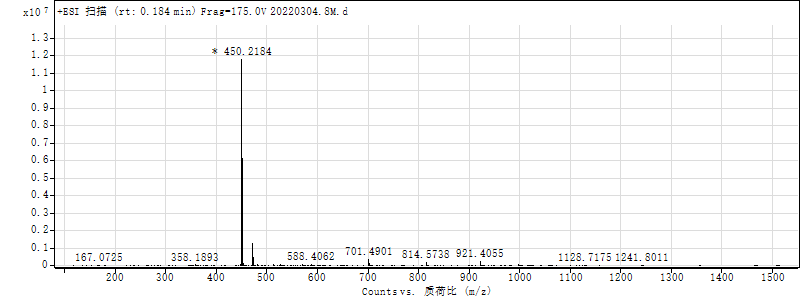


ESI-HRMS (TOF) of compound **8m**


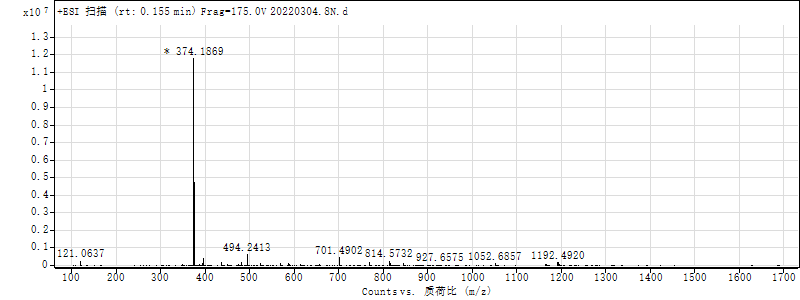


ESI-HRMS (TOF) of compound **8n**

**
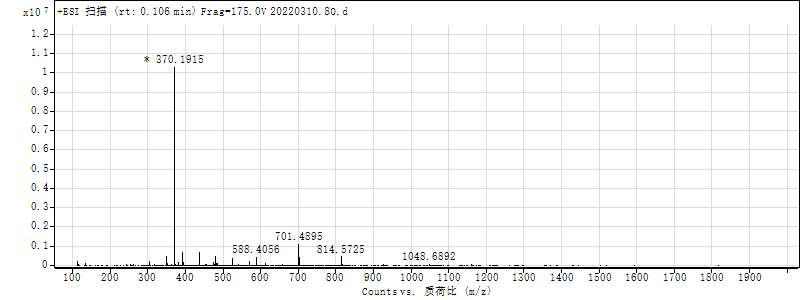
**

ESI-HRMS (TOF) of compound **8o**

**
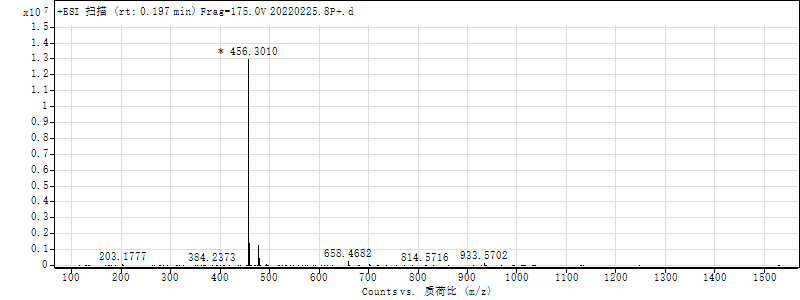
**

ESI-HRMS (TOF) of compound **8p**

**
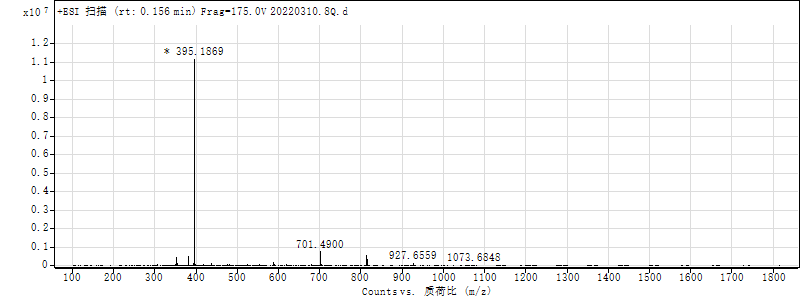
**

ESI-HRMS (TOF) of compound **8q**

**
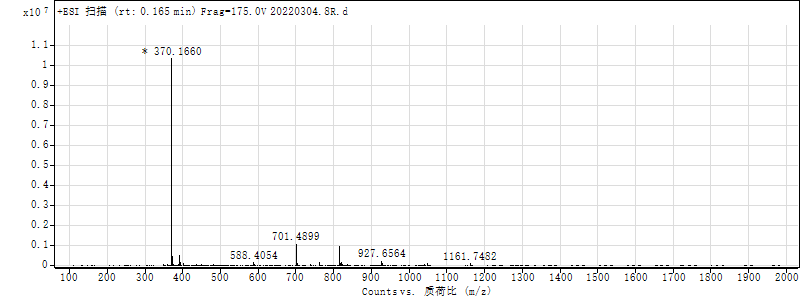
**

ESI-HRMS (TOF) of compound **8r**

**
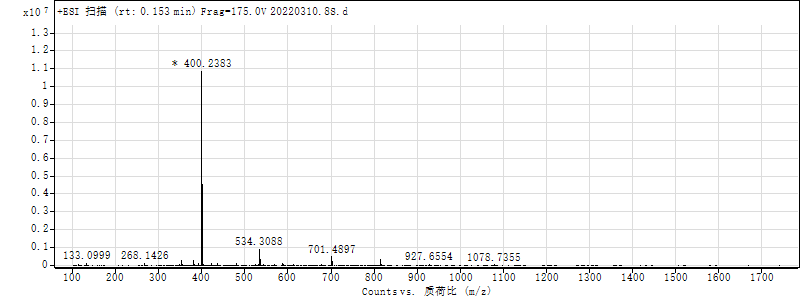
**

ESI-HRMS (TOF) of compound **8s**

**
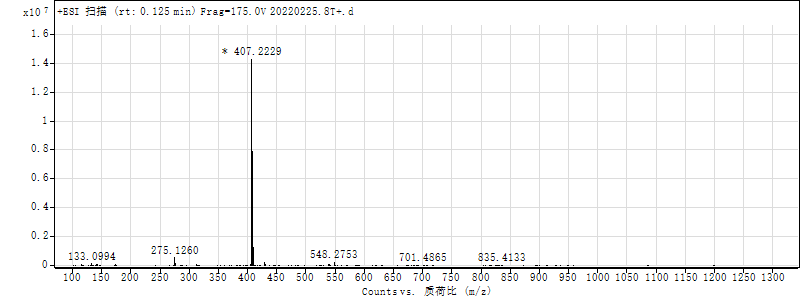
**

ESI-HRMS (TOF) of compound **8t**

**
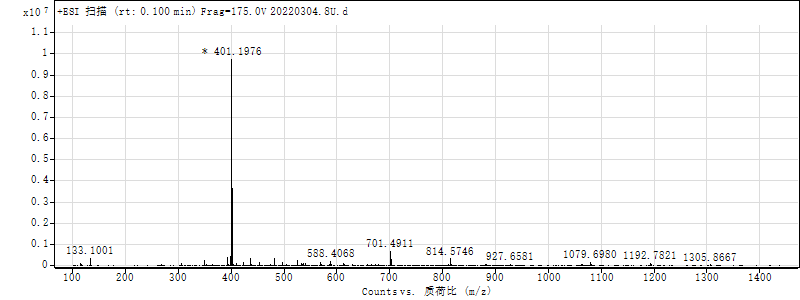
**

ESI-HRMS (TOF) of compound **8u**

**
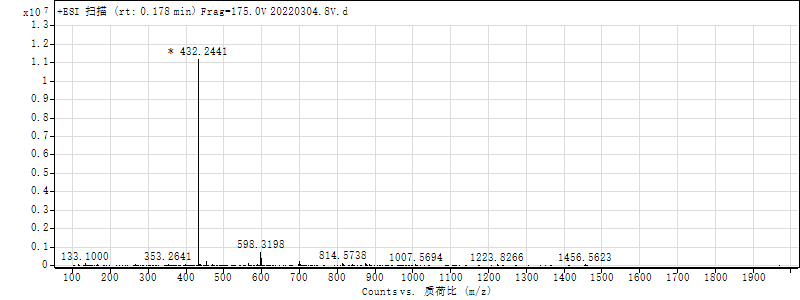
**

ESI-HRMS (TOF) of compound **8v**

**
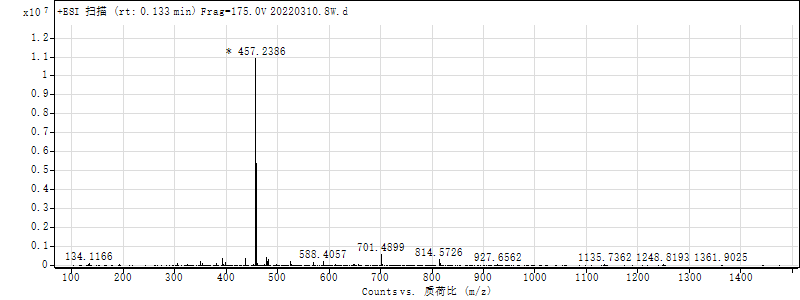
**

ESI-HRMS (TOF) of compound **8w**

**
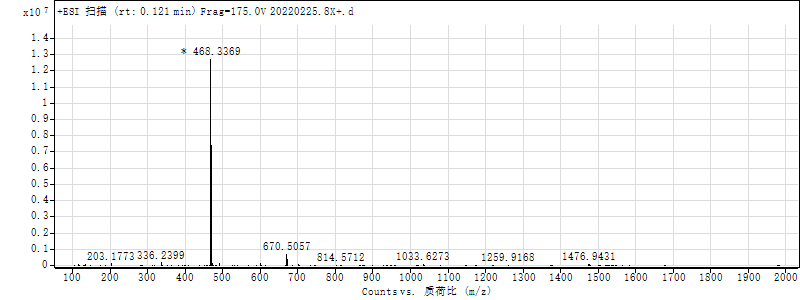
**

ESI-HRMS (TOF) of compound **8x**

**
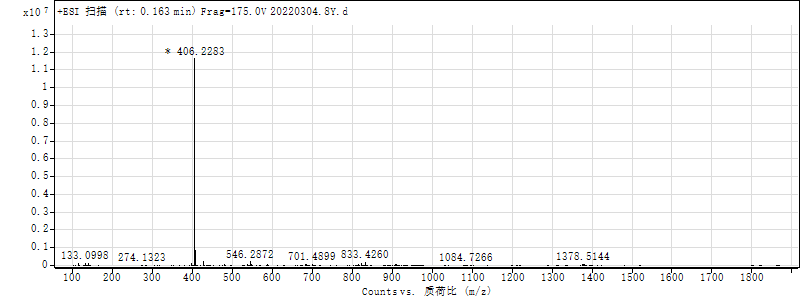
**

ESI-HRMS (TOF) of compound **8y**

ESI-HRMS (TOF) of compound **8z**

ESI-HRMS (TOF) of compound **9a**

ESI-HRMS (TOF) of compound **9b**
